# Diversity Modification and Structure-Activity Relationships of Two Natural Products 1β-hydroxy Alantolactone and Ivangustin as Potent Cytotoxic Agents

**DOI:** 10.1038/s41598-018-20192-9

**Published:** 2018-01-29

**Authors:** Jiang-Jiang Tang, Qiu-Rui He, Shuai Dong, Xin Guo, Yu-Gong Wang, Bei-Lei Lei, Jun-Mian Tian, Jin-Ming Gao

**Affiliations:** 1 Shaanxi Key Laboratory of Natural Products & Chemical Biology, College of Chemistry & Pharmacy, Northwest A&F University, Yangling, 712100 China; 2 College of Life Sciences, Northwest A&F University, Yangling, 712100 China

## Abstract

Sesquiterpene lactones (STLs) are a class of plant secondary metabolites widely found in nature with potent antitumor activities. In this work, two isolated STLs 1β-hydroxy alantolactone (**1**) and ivangustin (**2**) were derivatized through diversity-oriented strategy, and *in vitro* cytotoxic activity assessments were conducted against six cell lines including HeLa, PC-3, HEp-2, HepG2, CHO and HUVEC. The cytotoxic structure-activity relationship showed that the double bond between C5 and C6 was beneficial to improve activity; C1-OH oxidized derivatives showed a slight stronger activity, comparable to the positive drug etoposide (VP-16). Yet, C1-OH esterified derivatives decreased the potency which were different from those of 1-*O*-acetylbritannilactone (ABL) reported previously by us, and C13-methylene reductive and spiro derivatives resulted in almost complete ablation of cytotoxic activity. Mechanistic basis of cytotoxicity of the representative compound **1i** was assayed to relate with apoptosis and cell cycle arrest. Furthermore, **1i** inhibited TNF-α-induced canonical NF-κB signaling in PC-3 cells. Molecular modeling studies exhibited additional hydrogen bond interaction between **1i** and the residue Lys37 of p65, indicating that **1i** could form covalent protein adducts with Cys38 on p65.

## Introduction

**Figure 1:**
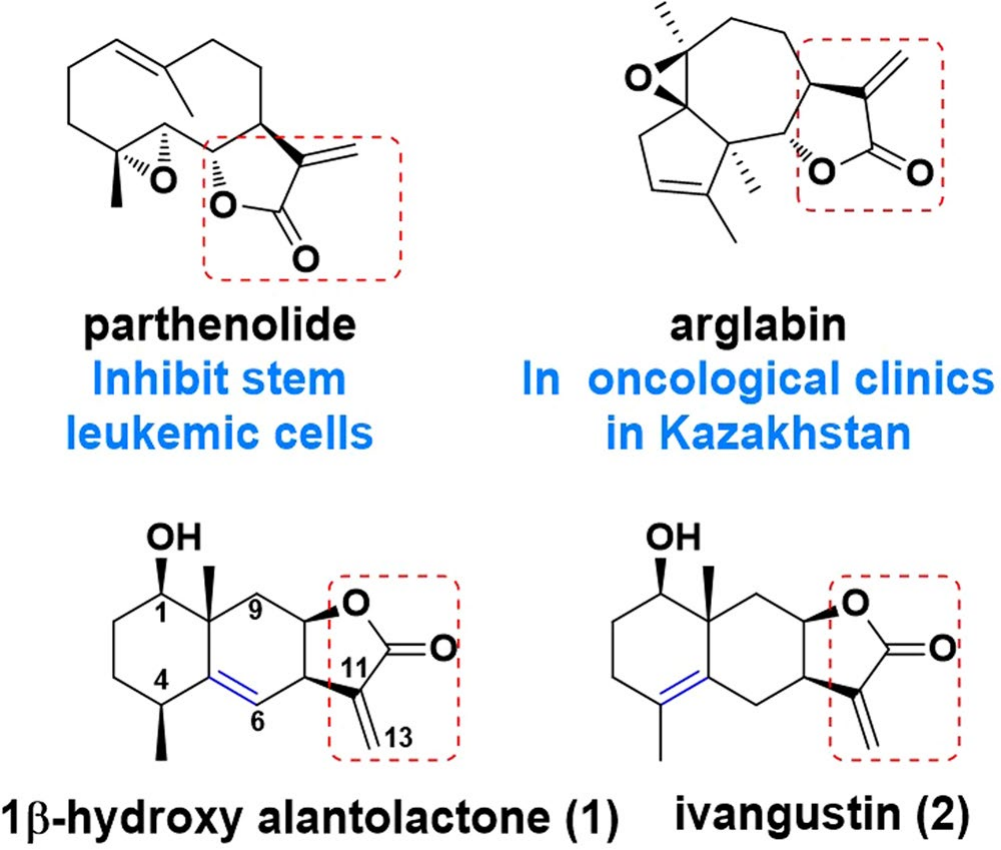
The structures of two clinical STLs parthenolide (from *Tanacetum parthenium*) and arglabin (from *Artemisia myriantha*), 1β-hydroxy alantolactone (**1**) and ivangustin (**2**) from *Inula britannica*.

The use of natural products as scaffolds for the generation of chemically diverse screening libraries is one of the effective methods for drugs screening^1^. It has been statistically reported that almost 50% clinically used anticancer agents since 1940 are either natural products or their direct derivatives^2^. Sesquiterpene lactones (STLs) are main plant-derived bioactive composites used in traditional medicines against inflammation and cancer^3–7^. During the recent four decades, STLs with α-methylene-γ-lactone moiety have attracted a lot of attention because of their broad spectrum of biological effects such as multi-target agents interfering with several processes involved in cancer development and progression^8^. Two renowned examples of STLs are parthenolide and arglabin: parthenolide exhibits distinct inhibition potency against human acute myelogenous leukemia stem/progenitor cells by induced-apoptosis^9,10^; arglabin has been already used as a drug in oncological clinics in Kazakhstan^11,12^ (Fig. 1). However, STLs still need to be optimized to possess more effective potency because of not undergoing evolutionary selection for human therapeutics. Recently, extensive research has been carried out to optimize active STLs by synthesizing diverse derivatives and to characterize their molecular mechanisms of action^13–19^.

1β-hydroxy alantolactone (1) and ivangustin (2), the most common 6/6/5-tricyclic eudesmane STLs, have been isolated from *Inula* genus^3,20,21^ (*Inula japonica*, *Inula britannica* and *Inula helenium*) plants (Fig. 1). The two compounds containing α-methylene-γ-lactone moiety have several biological effects, including anti-tumor and anti-inflammation activity^3,22^. From the point of view of chemical skeleton, the difference of **1** and **2** is just the location of a double bond in one of the six-membered rings, the double bond at C5 and C6 position for **1** and at C4 and C5 position for **2**. Previous anti-proliferative activity showed that **1** presented slightly strong cytotoxic activities compared with **2**, implying that the double bond at the C5-C6 position may be more efficient for the activity^22^. Besides, α-methylene-γ-lactone is recognized active site, where the electrophilic part can alkylate thiols of proteins or residues to induce the DNA-fragmentation and apoptosis through cells glutathione depletion^23–26^. However, to our knowledge, detailed anti-proliferative structure-activity relationship (SAR) for **1** and **2** have been not reported up to now and the mechanism of action remains unknown.

Previously reported synthetic modifications to STLs resulted in variation of anticancer activity, including esterification and oxidation of hydroxyl group (–OH), reduction, amination and coupling reaction of α-methylene-γ-lactone motif and cyclopropanation of double bond^19,27–32^. We have found that 1-*O*-acetyl-6-*O*-lauroylbritannilactone (ABL-L), an esterified derivative of 1-*O*-acetylbritannilactone (ABL), showed 4–10 fold stronger suppression against cancer cells by inducing apoptosis via a p53-dependent pathway^19,33^. 1β-Hydroxy alantolactone (**1**) retaining C1-OH exhibited stronger anti-inflammatory effect in RAW 264.7 cells^34^. In our ongoing endeavor on enriching chemical diversity of the STLs molecular framework to discover pharmacologically interesting compounds, in this study, we prepared a series of new derivatives of **1** and **2** through esterification, oxidation, reduction and [3 + 2] reactions at their C1 or C13 positions. Especially, the C13-methylene was converted to spirobislactone and spiro[lactone-isoxazol] derivatives by [3 + 2] reactions including radical [3 + 2] cyclization and 1,3-dipolar cycloaddition, which enriched the diversity of these natural products with α-methylene lactone motif. The *in vitro* anticancer activities were tested against four human cancer cell lines and two normal cell lines, and the preliminary anti-proliferative SAR was discussed. Furthermore, the most active compound **1i** was selected for further experiments and to determine its mechanistic basis of action in the PC-3 cell line.

## Results and Discussion

### Chemistry

**Figure 2:**
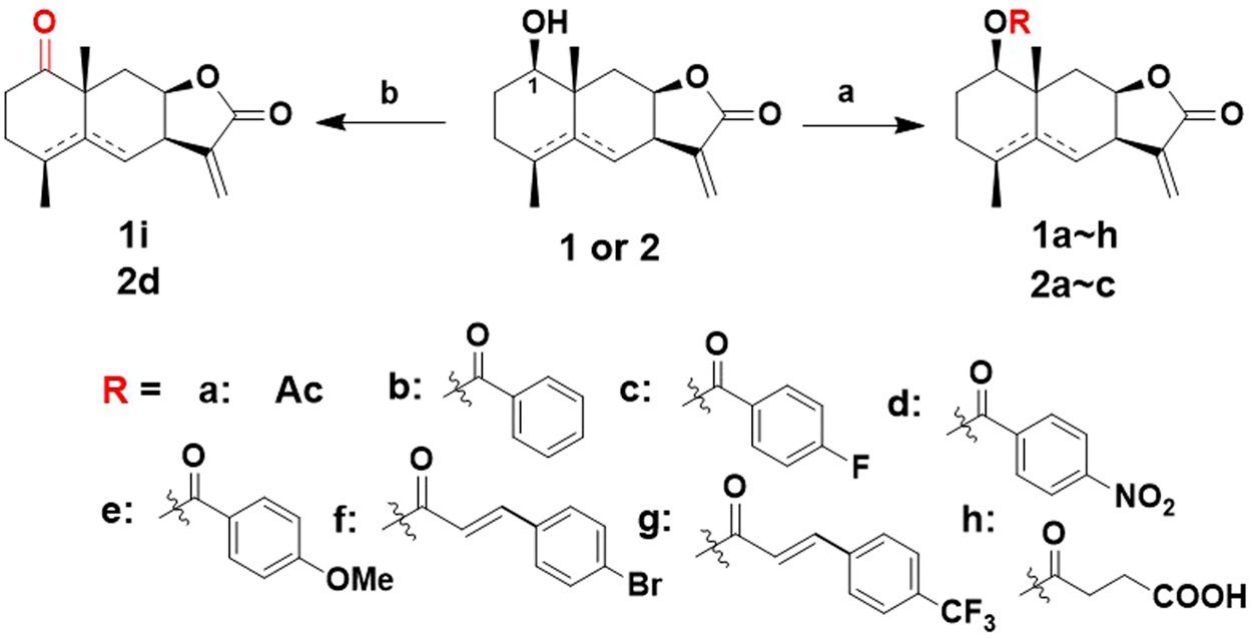
Synthesis of C1-OH modified derivatives of 1β-hydroxy alantolactone (**1**) and ivangustin (**2**). Conditions and reagents: (**a**) anhydride, Et_3_N, DMAP, rt, for **1a**, **1b**, **1h** and **2a**, **2b**; benzoyl chloride, pyridine, 0 °C for **1c**, **1e**, **1d** and **2c**; cinnamic acid, DMAP, DCC, 60 °C for **1f**, **1g** and **2d**; 30 min–8 h, 45–90%; (**b**) Dess-Martin periodinane (DMP), CH_2_Cl_2_, rt, 85% for **1i**^34^, 76% for **2d**.

**Figure 3:**
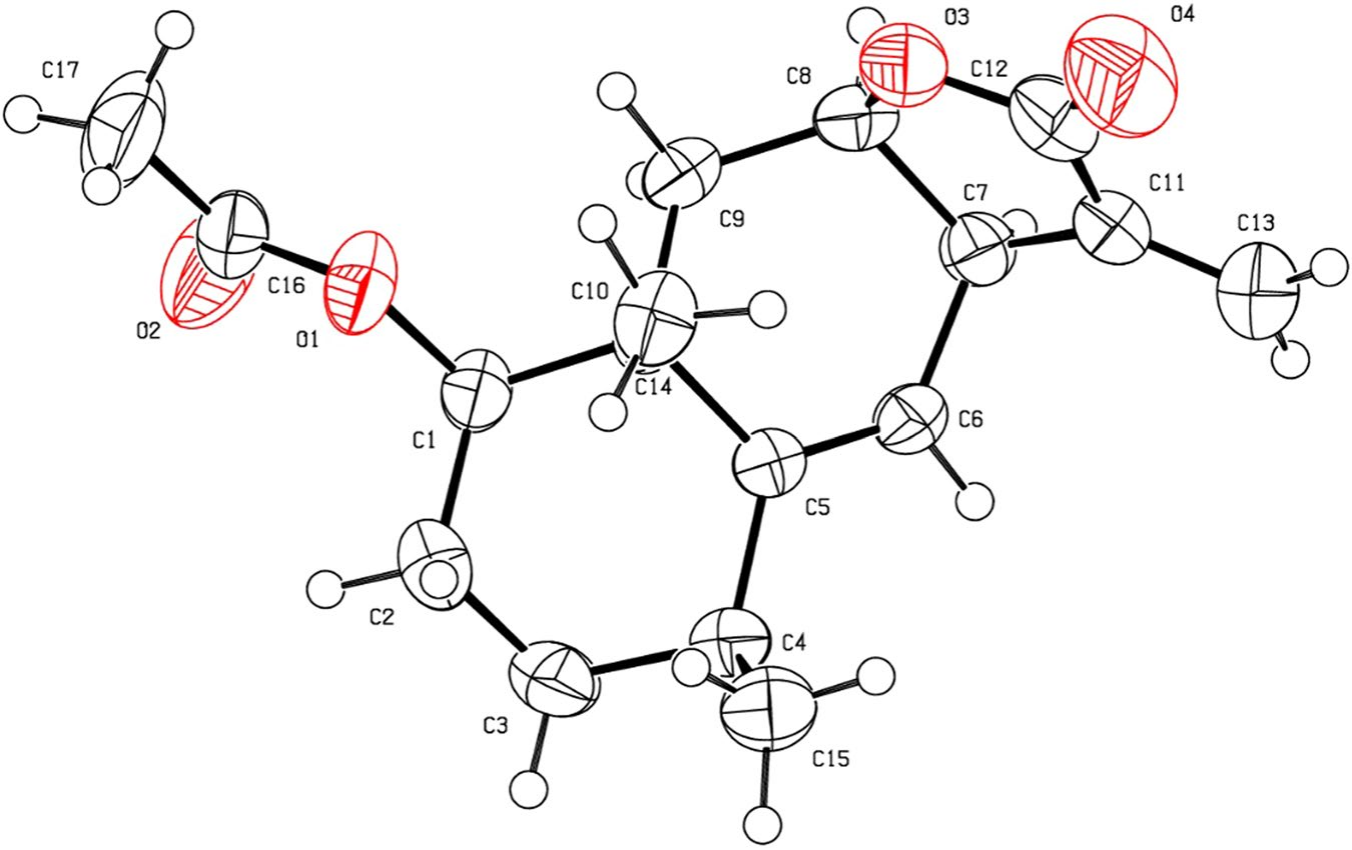
The X-ray crystal structure of **1a** (CCDC: 1504780).

The EtOAc-soluble fraction of the ethanolic extract of the dried flowers of *I*. *britannica* was repeatedly passed through column chromatography (silica gel), followed by purification by preparative TLC to afford 1β-hydroxy alantolactone (**1**) and ivangustin (**2**) in previous papers by others and us^3,22,35^. As illustrated in Fig. 2, **1** and **2** were converted to the corresponding ester derivatives **1a**–**h** and **2a**–**c** with aliphatic chain and aromatic moieties using different anhydrides or benzoyl chloride or cinnamic acid^34^. Geometric structure of the derivative **1a** was further verified by X-ray crystallography (Fig. 3). Oxidation of C1-OH of compounds **1** and **2** was carried out with mild oxidant Dess-Martin periodinane (DMP) to afford **1i**^34^ and **2d** (Fig. 2).

**Figure 4:**
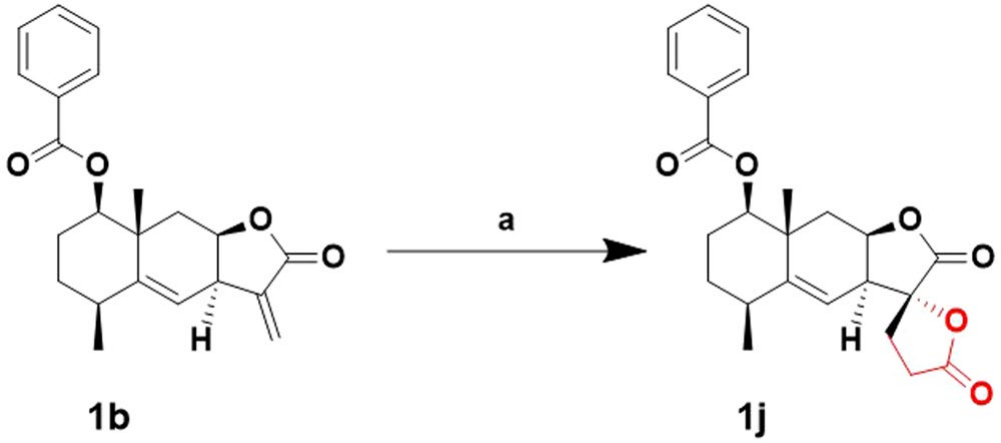
Synthesis of C13-methylene modified spirobislactone **1j**. Conditions and reagents: (**a**) Mn(OAc)_3_.2H_2_O, KOAc, AcOH, reflux, 3 h, 76%.

**Figure 5:**
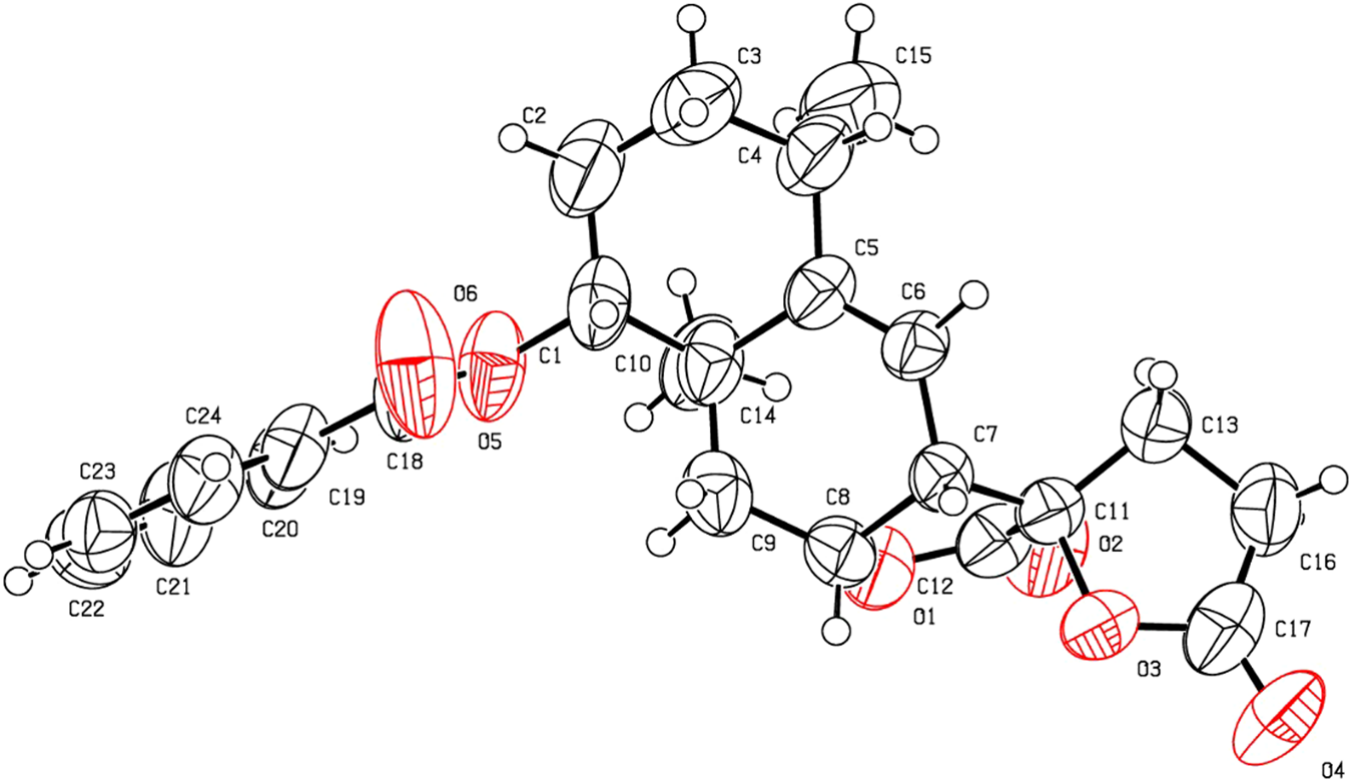
The X-ray crystal structure of **1j** (CCDC: 1504781).

Since the α-methylene-γ-lactone moiety in the STLs skeleton is one of major active sites, modifications of this moiety were being conducted^18,30,36^. It was reported that natural products bearing spirobicyclic moiety usually exhibited high biological activity^17,37–40^, such as inducing autophagy activity by clionamine D^40^. In order to discover interesting spiro compounds of STLs, a spirobislactone skeleton was conducted through a radical [3 + 2] cycloaddition reaction. As shown in Fig. 4, **1b** with aromatic ring possessing UV-detectable TLC profile was carried out the cycloaddition with Mn(OAc)_3_ in acetic acid under reflux for 24 h. However, the reaction did not occurred until addition of KOAc. When 2 equivalent of KOAc was added in the reaction solution, TLC detection for the reaction showed the complete consumption of the starting material, and the spirobislactone **1j** was given in 76% yield. The structure and 11*R*-stereochemistry of **1j** were assigned through its NMR spectroscopic analysis (Table 1) and were further confirmed by the X-ray crystallography (Fig. 5). The mechanism of the addition of acetic acid to alkenes to provide lactones has been studied by Fristad and co-workers^41^. It was disclosed that the rate determinating step in the oxidation of HOAc by Mn(OAc)_3_•2H_2_O is the loss of a proton from complex to give an complexed enolate. Thus, the presence of KOAc can increase the concentration of acetate ion (^–^OAc) and then accelerate the following free radical reaction. The stereoselectivity of free radical cycloaddition was clearly due to the steric effect, which the complexed free radical approached α-methylene-γ-lactone of **1b** on the less hindered face and then underwent lactonization to provide **1j**.

**Table 1:** ^1^H (500 MHz) and ^13^C (125 MHz) NMR spectroscopic data for compound **1j** and **1o** (in CDCl_3_, δ in ppm).

Position	1j		1o	
	δ_H_ (*J* in Hz)	δ_C_	δ_H_ (*J* in Hz)	δ_C_
1	4.71 dd (11.7, 4.1)	81.9		212.5
2	1.96 m; 1.77 m	22.4	2.74 m; 2.24 m	35.5
3	1.71 m; 1.60 m (overlap)	29.2	1.99 m; 1.79 m	28.2
4	2.52 m (overlap)	38.1	2.70 m	36.6
5		152.2		149.9
6	5.23 d (3.1)	113.8	5.34 d (3.3)	116.2
7	3.01 m	43.6	3.08 m	43.0
8	4.99 m	76.6	5.18 m	76.4
9	2.34 m (overlap); 1.60 m (overlap)	38.9	2.53 dd (15.6); 1.90 dd (15.6)	33.8
10		37.8		47.2
11		87.2		89.5
12		173.1		173.1
13	2.34 m (overlap)	25.2	3.67 d (16.9); 3.48 d (16.9)	37.1
14	1.37 s	23.3	1.44 s	28.1
15	1.11 d (7.6)	22.9	1.30 d (7.3)	23.7
16	2.95 m; 2.52 m (overlap)	28.1		156.0
17		174.7		121.0
18		166.0	7.64 d (8.8)	128.6 (2C)
19		130.2	6.94 d (8.8)	114.3 (2C)
20	7.97 d (7.5)	129.6 (2C)		161.6
21	7.39 t (7.7)	128.5 (2C)	3.85 s (O*C*H_3_)	55.4 (O*C*H_3_)
22	7.51 t (7.4)	133.2

**Figure 6:**
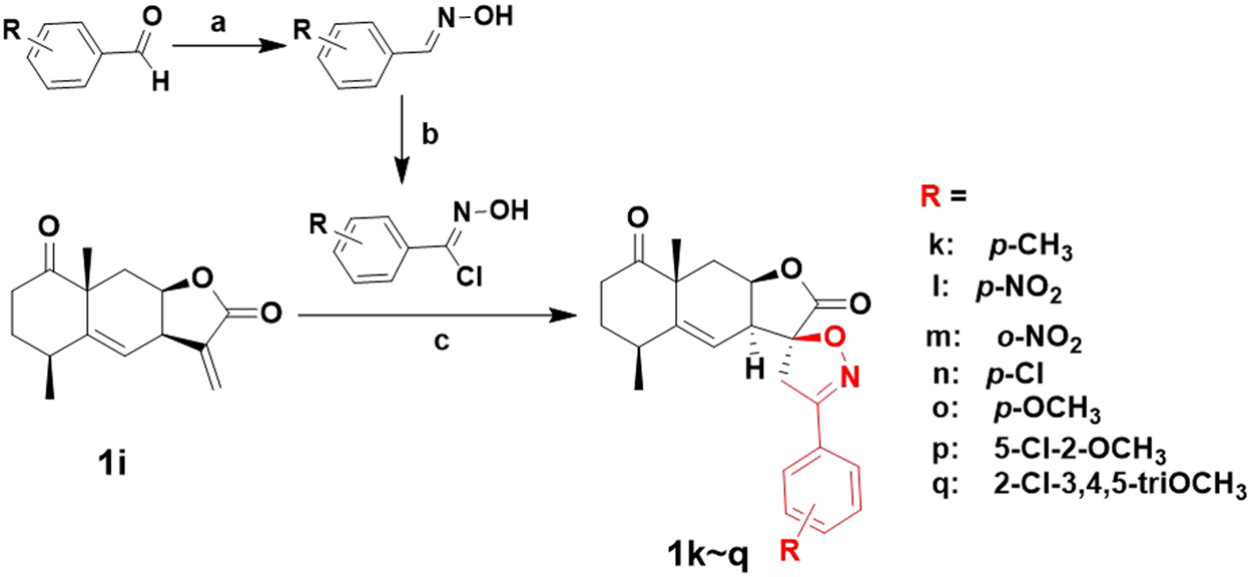
Synthesis of C13-methylene modified spiro[lactone-isoxazol] **1k**–**q**. Conditions and reagents^34^: (**a**) 50% NH_2_OH in H_2_O, Et_2_O, 89%; (**b**) DMF, NCS, rt, 98%; (**c**) CH_2_Cl_2_, Et_3_N, rt, 26–96%.

**Figure 7:**
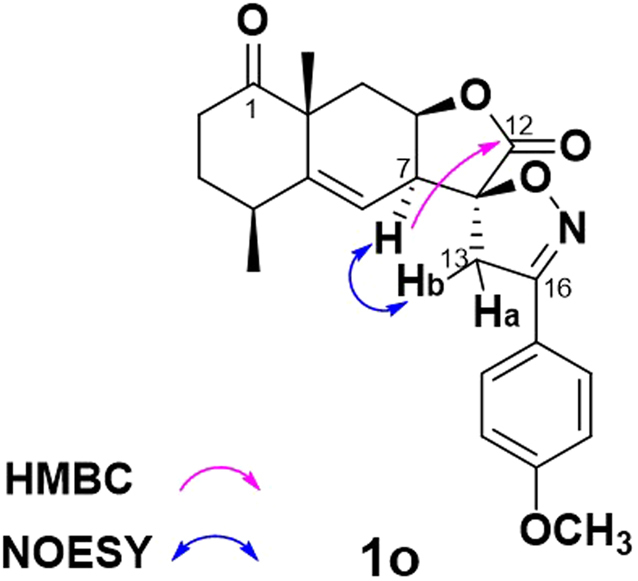
Selected HMBC spectra and key NOE correlation of **1o**.

Meanwhile, another spirobicyclic derivatives **1k**–**q** were prepared via 1,3-dipolar cycloaddition of **1i**^34^. As described in Fig. 6, treatment with new-made aldoxime chlorides with Et_3_N generated the corresponding nitrile oxides and then underwent 1,3-dipolar cycloaddition with **1i** leading to spiro[lactone-isoxazol] derivatives **1k**–**q** exclusively. The regiochemistry of the 1,3-dipolar cycloaddition was confirmed by the appearance of two doublets with respective chemical shift of 3.67 and 3.48 ppm in the ^1^H NMR spectra of spiro[lactone-isoxazol] **1o**, which were assigned to the proton signals of C13-methylene on the isoxazoline ring. Meanwhile, in the HMBC spectrum of **1o**, a typical long-range correlation between H-7 (δ_H_ = 3.08 ppm) and C-12 (δ_C_ = 173.09 ppm) was observed, but another correlation between H-7 and C-16 (δ_C_ = 155.98 ppm) was absent in Fig. 7. Furthermore, the NOE correlation between the two hydrogens of H-7 and H-13b (δ_H_ = 3.48 ppm) was clearly observed in its NOESY spectroscopic analysis, indicating 11*S*-configuration of **1o**. The excellent regioselectivity may be due to the electronic effect of the vinyl moiety under the effect of the carbonyl moiety, and the exclusive 11*S*-stereoselectivity of the 1,3-dipolar cycloaddition was apparently due to the steric repulsion between the lactone ring of **1i** and the newly formed isoxazoline ring, respectively.

**Figure 8:**
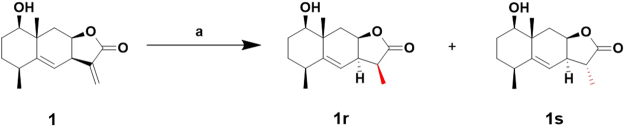
Synthesis of C13-methylene reductive derivatives **1r** and **1s**. Conditions and reagents: (**a**) NaBH_4_, THF, 2 h, rt, **1r** for 67%^34^, **1s** for 20%.

**Figure 9:**
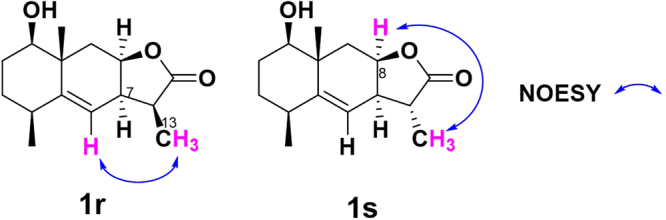
Selected NOE correlations of **1r** and **1s**.

Reductive derivatives of C13-methylene were synthesized as well to compare their activities. As shown in Fig. 8, reduction of **1** with NaBH_4_ in THF at room temperature formed two 1β-hydroxy dihydroalantolactone derivatives **1r**^34^ and **1 s** in 87% overall isolated yield (**1r** for 67%, **1 s** for 20% yield). The relative configurations of the two diastereomers were deduced similarly by their 2D NMR spectroscopic data. The assignment of the C11 spatial configuration of **1r** and **1 s** was determined to be the 11*S* and 11*R*, respectively. NOESY signals between H-6 (5.26 ppm) and H-13 (1.23 ppm) of **1r** indicated the 11*S*-configuration, whereas the NOESY signals between H-8 (4.91 ppm) and H-13 (1.35 ppm) of **1 s** indicated the 11*R*-configuration (Fig. 9). This stereoselective difference using NaBH_4_ as reducing agent was also rightly due to the steric effects.

These all derivatives of **1** and **2** were confirmed by 1D or 2D NMR and (HR)ESI-MS. The purity of all compounds was higher than 95% verified by HPLC with UV prior to their evaluation of biological efficacies. It was noted that the [3 + 2] reactions in constructing spirobislactone and spiro[lactone-isoxazol] derivatives would provide new skeletons for this kind of natural products with methylene motif.

### Biological evaluation

#### Cytotoxic activity

All the synthesized derivatives of **1** and **2** were screened for their anticancer activities against four human cancer cell lines (HeLa, PC-3, HEp-2 and HepG2) and two normal cell lines (CHO and HUVEC) using the sulforhodamine B (SRB) assay^42^. Parthenolide (**P**) and a well-known anticancer drug etoposide (VP-16) were used as positive controls and IC_50_ values (50% inhibition concentration of cell viability) of the tested compounds were summarized in Tables 2 and 3.

**Table 2:** Cytotoxic activities (IC_50_) of 1β-hydroxy alantolactone (1) and ivangustin (2) and their C1-OH modified derivatives.

No.	IC_50_^*a*^ (μM)					
	HeLa	PC-3	HEp-2	HepG2	CHO	HUVEC
**1**	3.2 ± 0.8	4.5 ± 0.7	3.3 ± 0.2^*b*^	5.2 ± 0.1	6.4 ± 0.1^*b*^	9.2 ± 0.1
**1a**	8.2 ± 1.0	8.1 ± 0.3	10.3 ± 1.8	6.3 ± 1.3	10.3 ± 1.8	12.3 ± 1.6
**1b**	25.5 ± 3.2	>40	>40	>40	ND^*d*^	ND
**1c**	>40	>40	>40	>40	ND	ND
**1d**	>40	>40	>40	23.5 ± 3.6	ND	ND
**1e**	>40	>40	>40	34.1 ± 4.8	ND	ND
**1f**	>40	18.7 ± 5.2	>40	12.5 ± 2.2	ND	ND
**1g**	10.0 ± 2.1	11.8 ± 2.8	>40	15.2 ± 3.8	ND	ND
**1h**	>40	>40	>40	>40	ND	39.5 ± 2.5
**1i**	2.7 ± 0.1	2.5 ± 0.8	3.5 ± 0.9	5.1 ± 0.2	2.5 ± 0.2	8.8 ± 0.7
**2**	7.7 ± 1.3	10.8 ± 2.9	4.8 ± 0.4^*b*^	8.9 ± 2.2	8.5 ± 0.1^*b*^	16.6 ± 1.8
**2a**	32.3 ± 2.0	28.9 ± 4.5	>40	>40	ND	ND
**2b**	27.9 ± 2.5	>40	>40	>40	ND	ND
**2c**	>40	>40	>40	>40	ND	ND
**2d**	8.6 ± 1.4	7.0 ± 2.1	14.1 ± 2.9	7.7 ± 1.5	12.1 ± 5.2	10.5 ± 0.5
VP-16	3.0 ± 0.3^*c*^	0.6 ± 0.1	4.8 ± 0.5^*c*^	7.6 ± 0.8	2.6 ± 0.2^*c*^	1.9 ± 0.2
**P**	7.6 ± 0.6	3.4 ± 0.6	6.8 ± 0.3	—	5.3 ± 0.7	—

^*a*^The IC_50_ values represent the concentration that causes 50% inhibition of cell viability. Cells were treated with compounds for 72 h. All data (mean ± SD) are the average of three or four independent experiments in triplicate. Cancer cell lines: HeLa (human cervix cancer), PC-3 (human prostate cancer), HEp-2 (human larynx epidermal cancer), HepG2 (human liver cancer); Normal cell lines: CHO (Chinese hamster ovary), HUVEC (human umbilical vein endothelial cell). VP-16 represents etoposide, and **P** represents parthenolide. ^*b*^These values derive from Xiang *et al*.^22^. ^*c*^Derive from Dong *et al*.^19^. ^*d*^ND: not detected.

**Table 3:** Cytotoxic activities (IC_50_) of C13-methylene modified derivatives.

No.	IC_50_^*a*^ (μM)	
	PC-3	HepG2
**1j**	>40	>40
**1k**	>40	>40
**1l**	>40	>40
**1m**	>40	>40
**1n**	>40	>40
**1o**	>40	>40
**1p**	>40	>40
**1q**	>40	>40
**1r**	>50	>50
**1s**	>50	>50

^a^Cells were treated with compounds for 72 h. All data (mean ± SD) are the average of three or four independent experiments in triplicate.

As seen from IC_50_ values of Table 2, natural products **1** showed slightly better potential than **2** against HeLa, PC-3, HEp-2 and HepG2 cells with the IC_50_ ranges for **1** with 3.2–6.4 μM and for **2** with 4.8–6.4 μM. Their esterified derivatives **1a**–**h** and **2a**–**c** containing introduction of aliphatic chain (acetyl and carboxypropionyl) or aromatic groups (benzoyl and cinnamoyl) at C1-OH position exhibited weaker cytotoxic activity than **1** and **2** against these cancer cell lines. It is different from previous reports about another STLs 1-*O*-acetylbritannilactone (ABL)^18,19^ by us and others^31,32^, where ABL esterified derivatives have shown stronger suppression against cancer cells than ABL. These results indicated that C1-OH is important for activity in the two natural molecules **1** and **2**. Meanwhile, C1-OH oxidized derivatives **1i** and **2d** displayed the similar or slightly better potency than that of the parent **1** and **2**, which was also different from anticancer SAR of ABL^19^ and anti-inflammatory SAR of **1**^34^. These differences may be due to their different molecular geometry or lipophilicity or chemical environment. In addition, by comparing IC_50_ data of compounds **1a** and **2a**, **1b** and **2b**, **1c** and **2c**, **1i** and **2d**, a similar tendency with **1** and **2** was observed that location of the double bond in C5 and C6 position seems to be more favorable for cytotoxic activity.

Spirobislactone and spiroisoxazol were introduced into the methylene motif of **1** to enrich the chemical diversity. From Table 3, it could be seen that spirobislactone **1i** and spiro[lactone-isoxazol] derivatives **1k**–**q** resulted in decreased potency (IC_50_ >40 μM on PC-3 and HepG2 cells) compared with that of the parent **1** and **1i**. Furthermore, reductive diastereomers **1r** and **1 s** also showed loss of cytotoxic activity, similar to their anti-inflammatory activity^34^. The results indicated the importance of the α-methylene functionality. Moreover, these active derivatives were assessed whether there was any sensitivity to normal versus cancer cells. Their cytotoxicity was measured against CHO and HUVEC. As shown in Table 2, the sensitivity of these compounds was weak for CHO cells with approximate IC_50_ data and was medium for HUVEC cells with 2–3 selectivity index (comparison with the IC_50_ of **1i** on between four cancer cells and HUVEC cells), implying that derivatives of **1** and **2** may have low selectivity toward cancer cells.

Among these tested derivatives, **1i** displayed the highest effect with IC_50_ values of 2.7, 2.5, 3.5 and 5.1 μM toward HeLa, PC-3, HEp-2 and HepG2 cells, respectively, which is comparable to VP-16 and parthenolide (**P**), the positve controls. Thus, **1i** was selected as a representative compound for detailed mechanistic investigations in PC-3 cells.

#### Apoptosis

**Figure 10:**
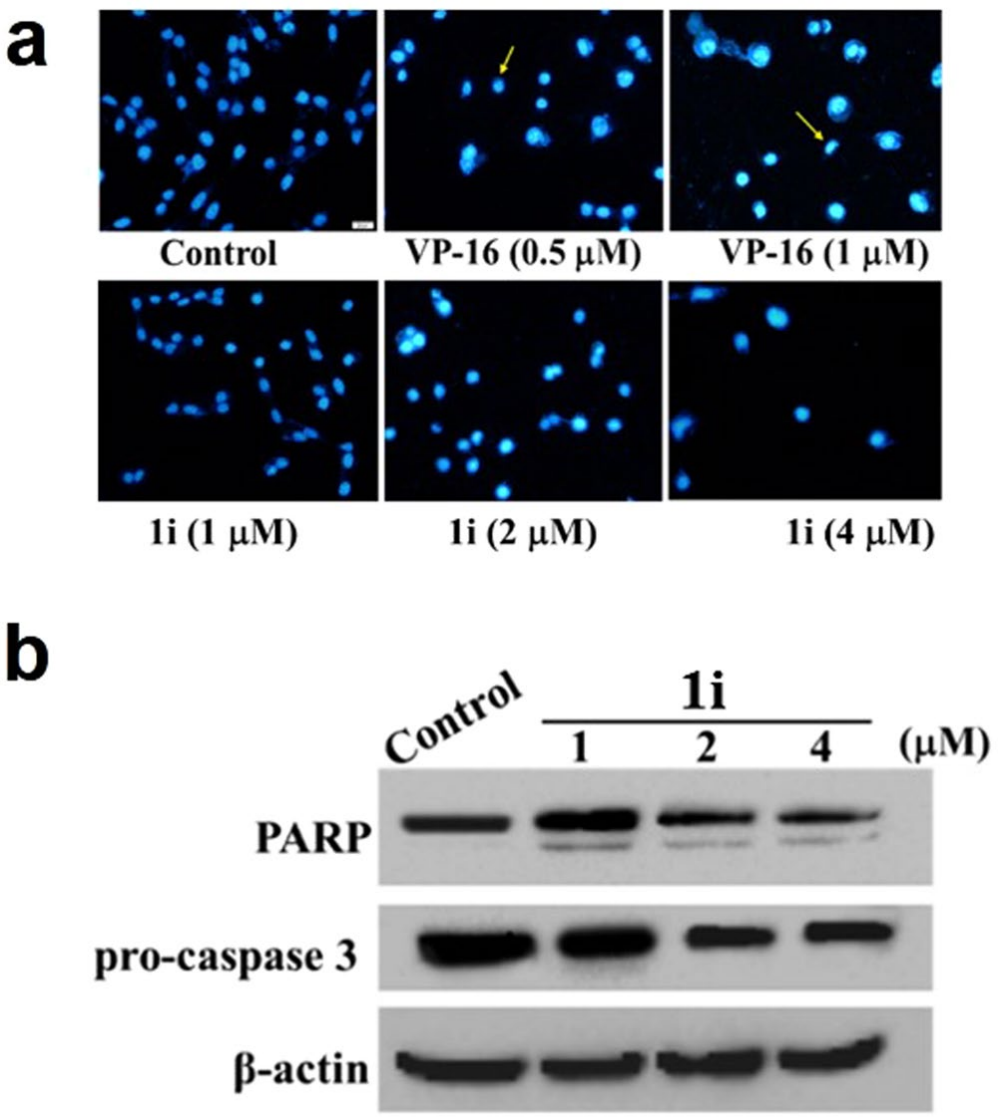
Induction of apoptosis by active compound **1i** and VP-16 at the indicated concentrations on PC-3 cells. (**a**) 72 h after the treatment of these compounds at the indicated concentrations, cells were fixed, washed with PBS, stained with Hoechst 33258, and analyzed for morphological characteristics associated with apoptosis by fluorescence microscopic analysis (×20). (**b**) Compound **1i** triggered changes of pro-caspase 3 and PARP by Western blotting analysis. Cells were lysed after 72 h treatment with **1i** at the concentration (1, 2 and 4 μM). The lysates were resolved on a 10% SDS-PAGE, transferred on to a nitrocellulose membrane and probed for cleaved caspase 3 and PARP. β-actin was used as a loading control.

Apoptosis is an important mechanism involved in the anticancer potency and apoptotic cells can be characterized with changes of nuclear morphology^43^. The active derivative **1i** was chosen to be investigated regarding its mechanism of action on PC-3 cells. After staining with Hoechst 33258, treated PC-3 cells with 0.5 and 1 μM VP-16 for 72 h showed fragmentation and condensation of chromatin, compared with the untreated control (Fig. 10a). Changes of nuclear morphology in HepG2 cells treated by **1i** were also seen in Figure S1, similar with treatment in PC-3 cells. This apoptotic tendency was apparent in **1i** at various concentrations (1, 2 and 4 μM). To reconfirm apoptotic cell death induced by **1i** in PC-3 cells, the cleavages of pro-caspase 3 and its substrate poly-ADP-ribose polymerase (PARP) were investigated by a western blotting analysis^44^. Shown in Fig. 10b, **1i** induced a significant dose-dependent decrease in pro-caspase 3 and the cleavage of its substrate PARP, demonstrating a proapoptotic activity of **1i**.

#### Cell cycle analysis

**Figure 11:**
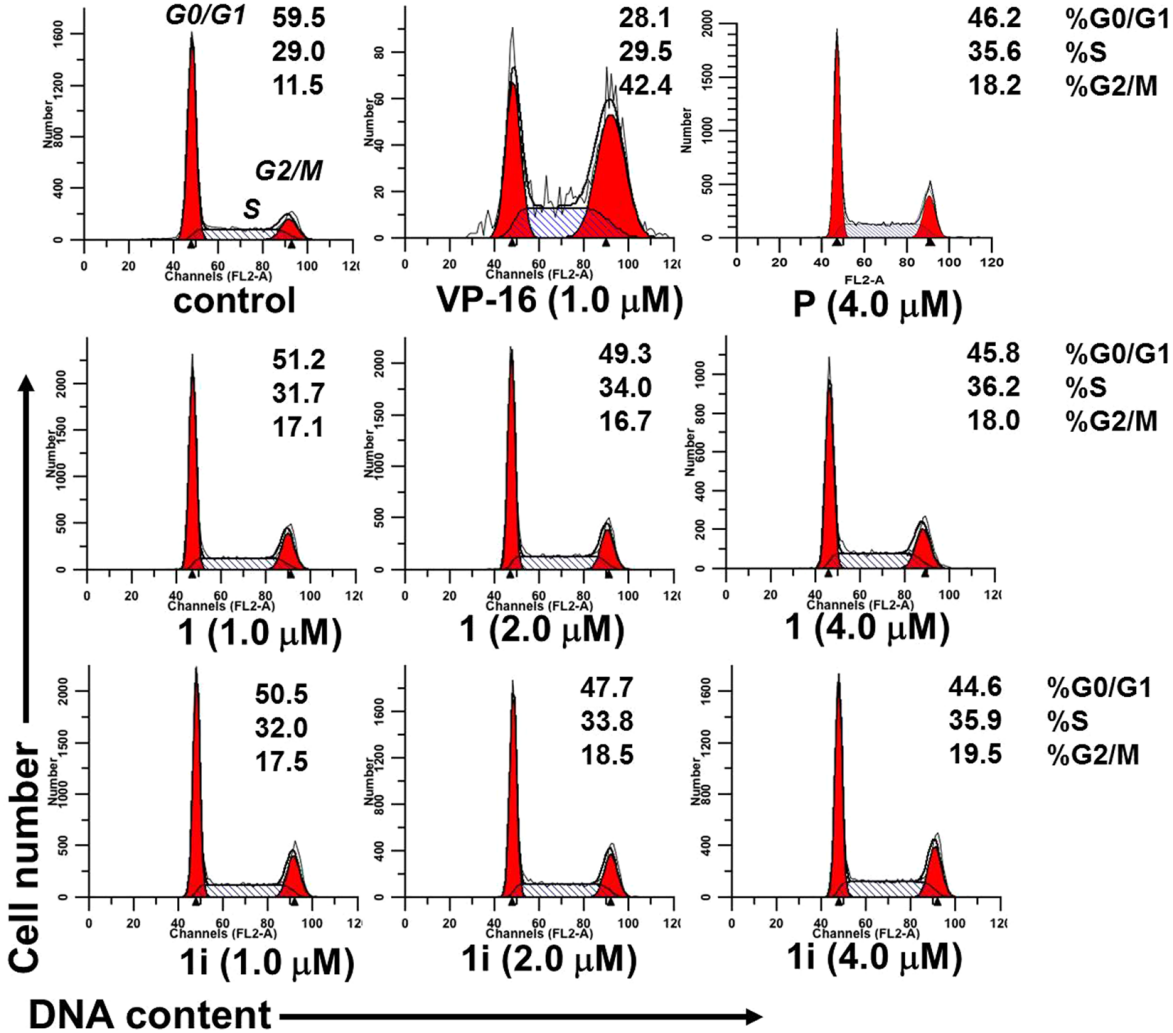
Effects of active compounds **1** and **1i** at the indicated concentrations on the cell cycle of PC-3 cells. The cultured cells were treated with these compounds at the indicated concentrations for 48 h, then harvested, and analyzed by flow cytometry. VP-16 and Parthenolide (**P**) treatments were set as positive groups. Each experiment was performed in triplicate.

To further investigate the effects by which active compound **1i** exerted their cytotoxic potencies, cell cycle distribution was analyzed by treating at various concentrations (1, 2 and 4 μM). Representative FACS measurements from the PC-3 cell line were shown in Fig. 11. Untreated cells were measured as control, and positive groups were treated with VP-16 (1.0 μM) and parthenolide (4.0 μM). When treated with VP-16, a significant increase in the proportion of cells in G2/M phase (42.4%) were detected compared to untreated control groups (11.5%), which is in accordance with the previous report^45^. Parthenolide (**P**) caused a decrease in the proportion of cells in G0/G1 phase (from 59.5% to 46.2%) with a concomitant increase of cells in other phases of the cell cycle. Similar to the action of **P**, compound **1** caused a decrease in the proportion of cells in G0/G1 phase in a concentration-dependent manner. Similar arrests were also observed in **1i** treatment groups (from 59.5% to 44.6%), revealing that the slightly superior cytotoxicity of **1i** over **1** was associated with a similar mechanism with that of parthenolide in cell cycle progression.

#### Inhibition of NF-κB signaling

**Figure 12:**
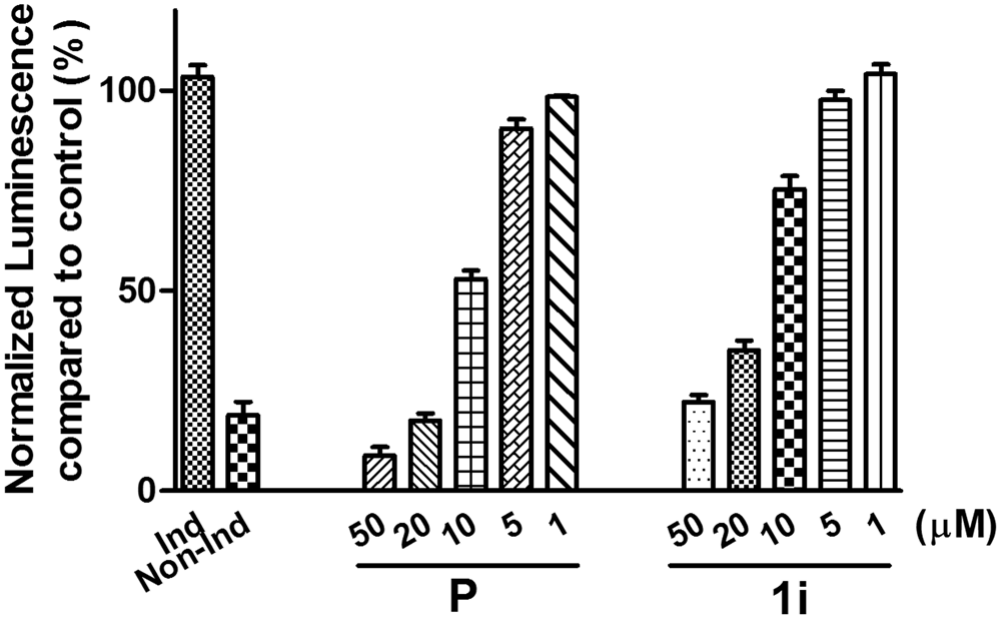
NF-κB-luciferase inhibition assay in PC-3 cells. PC-3 cells with a stably transfected NF-κB luciferase reporter were stimulated with TNF-α (50 ng/mL) (except for noninduced control, Non-Ind) and different dose of parthenolide (**P**) and **1i** for 8 h. Normalized luminescence was plotted as NF-κB luciferase activity compared to control (%).

The canonical NF-κB signaling pathway is a target for developing therapeutics for multiple human diseases, including cancer and chronic inflammatory diseases^46,47^. Many STLs are known modulators of NF-κB signaling^7,48^. In particular, two famous STLs parthenolide and helenalin have been certified to covalently target Cys38 of NF-κB p65 through hetero-Michael addition between exocyclic methylene butyrolactones and biological thiol of Cys38^49,50^. 1β-hydroxy alantolactone (**1**) has displayed dose-dependent inhibition towards the NF-κB pathway, like parthenolide^34^. To test the inhibition of active compound in NF-κB signaling, **1i** and parthenolide (**P**, positive control) were screened for inhibitory activity toward canonical p50/p65 NF-κB signaling with a cellular luciferase assay (Fig. 12)^51^. During this 8 h assay, cellular viability was >80% for all other doses of both compounds shown in Fig. 12. Parthenolide exhibited low micromolar inhibition of induced NF-κB signaling (52.1% NF-κB activity at 10 μM). **1i** also showed the inhibition activity in concentration-dependent manner (such as 22.4%, 35.5%, 75.6%, 99.2% at 50, 20, 10, 5 μM, respectively). The observation that **1i** and **P** are comparably potent in this assay hints at the possibility that **1i** may also have similar properties like 1β-hydroxy alantolactone and parthenolide in cells.

#### Molecular modeling study

**Figure 13:**
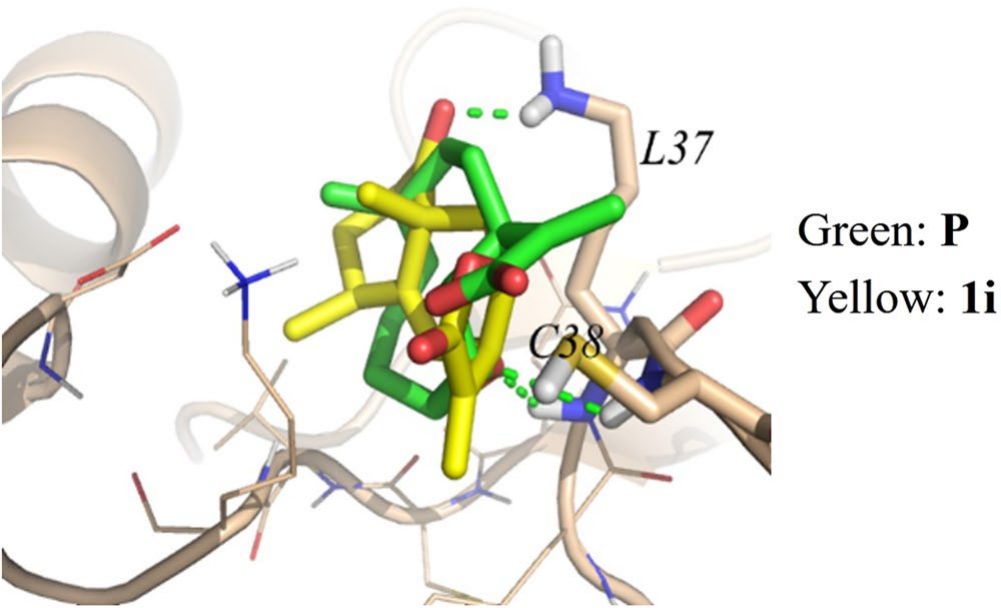
Predicted binding modes of Parthenolide (**P**) and **1i** to NF-κB/p65 (PDB 1VKX) according to the reference^34^. The docking experiments showed hydrogen bond (2.9 Å) interaction between Parthenolide (**P**) or **1i** and the residue Lys37 of p65.

Molecular modeling of 1β-hydroxy alantolactone (**1**) with p65 of NF-κB has showed **1** can superimpose well with parthenolide in a p65 binding site with Surflex-Dock protocol in Sybyl-X software package (PDB: 1VKX)^34^. Furthermore, the possible binding mode of **1i** with the p65 binding site was also performed. The docking study (Fig. 13) showed that **1i** superimposed better with parthenolide, displaying a distorted conformation. The spatial distance between the exocyclic methylene (C13) of **1i** and SH group of Cys38 is about 3.5 Å (<7.7 Å), similar to that of **1**^34^. However, the best binding exhibits a hydrogen bond (2.9 Å) interaction between C1 carbonyl oxygen atom of **1i** and the residue Lys37, while in the docking model of **1**, there is a longer spatial distance (3.8 Å) between C1-OH oxygen atom and Lys37, indicating that **1i** may more easily form covalent protein adducts with Cys38 on p65 than **1**.

## Conclusion

In the current work, we prepared a series of C1-OH and C13-methylene derivatives of 1β-hydroxy alantolactone (**1**) and ivangustin (**2**), in which the [3 + 2] cycloaddition reactions to convert the α-methylene lactone motif into spirobicyclic might be fit for structure-diversity modification of this kind natural products. Cytotoxic SAR results showed that the oxidized ketone of C1-OH shows slight stronger activity; the double bond in C5-C6 helps to improve activity; the retaining C13-methylene is crucial for activity. Besides, the representative derivative **1i** induced apoptosis characterized by morphological analysis and activation of caspase-3 against PC-3 cells. Subsequent flow cytometric analysis showed that **1** and **1i** are capable of decreasing the G0/G1 cells on PC-3 cells, similar to the action mechanism of parthenolide (**P**). Moreover, **1i** had inhibitory activity toward the canonical NF-κB signaling, and predicted binding modes showed that **1i** could more easily form covalent protein adducts with Cys38 on p65. The results indicated that the oxidized ketone **1i** could act as an anticancer potential hit and be further investigated for new anticancer leads by proper structure optimization.

## Methods

### Chemistry

#### General

NMR spectra were recorded on a 500 MHz Bruker NMR spectrometer in CDCl_3_ with TMS as internal standard for protons and solvent signals as internal standard for carbon spectra. Chemical shift values are mentioned in δ (ppm) and coupling constants (*J*) are given in Hz. ESI-MS spectra were recorded on an ESI-Thermo Fisher LTQ Fleet instrument spectrometer. HR-ESI-MS spectra were obtained on a Thermo Scientific LTQ Orbitrap (Thermo Scientific). Analytical HPLC was performed on a Waters 1525 series with an Agilent TC-C18 column and UV (PDA) detection at the max wavelength of compounds. Column chromatography (CC) was performed over silica gel (200–300 mesh, Qingdao Marine Chemical Ltd.). The progress of all reactions was monitored by TLC on 2 cm × 5 cm precoated silica gel GF_254_ plates of thickness of 0.25 mm (Qingdao Marine Chemical Group, Co.). Spots were visualized UV light (254, 365 nm) and/or by staining with 5% phosphomolybdic acid followed by heating. All commercially available solvents and reagents were freshly purified and dried by standard techniques prior to use.

#### Extraction and isolation

1β-hydroxy alantolactone (**1**) and ivangustin (**2**) were obtained from air-dried flower heads of *I*. *britannica* by repeated silica gel column chromatography and preparative TLC refered to a previous method^22^.

1β*-hydroxyalantolactone* (**1**)^52^. White powder; ${[{\rm{\alpha }}]}_{{\rm{D}}}^{30}$ = + 85.0° (*c* 0.030 in CHCl_3_); HPLC: t_R_ = 11.9 min, purity = 96.1% @ 215 nm, 50% methanol in water.

*ivangustin* (**2**)^52^. Cubic crystal; ${[{\rm{\alpha }}]}_{{\rm{D}}}^{30}$ = + 81.4° (*c* 0.037 in CHCl_3_); HPLC: t_R_ = 15.7 min, purity = 96.9% @ 260 nm, 50% methanol in water.

#### General procedure for the synthesis of 1a, 1b, 1h and 2a, 2b

To a suspension of anhydride (0.2 mmol), Et_3_N (0.3 mmol) and DMAP (0.01 mmol) in anhydrous CH_2_Cl_2_ (1 mL) in an ice-bath stirred for 30 min was added compound **1** or **2** (0.1 mmol) in anhydrous CH_2_Cl_2_ (1 mL) solution^34^. After completion of the reaction for 30 min at room temperature, ice water (2 mL) was added to the solvent and stirred for 20 min, then extracted with CH_2_Cl_2_, dried and filtered. After removal of the solvent, the crude product was purified by silica gel chromatography (EtOAc/PE) to afford compound **1a**, **1b**, **1 h** and **2a**, **2b**.

*1β-ethanoyl alantolactone* (**1a**)^34^. White powder; HPLC: t_R_ = 12.8 min, purity = 95.2% @ 210 nm, 60% methanol in water.

*1β-benzoyl alantolactone* (**1b**). White powder. Yield: 84% (25 mg); ${[{\rm{\alpha }}]}_{{\rm{D}}}^{30}$ = +164.8° (*c* 0.033 in CHCl_3_); ^1^H NMR (500 MHz, CDCl_3_): δ 8.05 (m, 2 H, Ph), 7.59 (m, 1 H, Ph), 7.47 (m, 2 H, Ph), 6.23 (d, *J* = 1.8 Hz, 1 H, H-13a), 5.65 (d, *J* = 1.6 Hz, 1 H, H-13b), 5.31 (d, 1 H, *J* = 4.0 Hz, H-6), 4.79 (m, 2 H, H-1, H-8), 3.59 (m, 1 H, H-7), 2.48 (m, 1 H, H-4), 2.43 (dd, *J* = 15.0, 2.9 Hz, 1 H, H-9a), 2.06 (m, 1 H, H-2a), 1.93–1.77 (m, 2 H, H-3a, H-2b), 1.68 (m, 2 H, H-3b, H-9a), 1.30 (s, 3 H, H-14), 1.21 (d, *J* = 7.6 Hz, 3 H, H-15); ^13^C NMR (125 MHz, CDCl_3_): δ 170.2 (C-12), 166.3 (Ph*C*OO-1), 147.1 (C-5), 139.5 (C-11), 133.2 (Ph), 130.31 (Ph), 129.7 (2 C, Ph), 128.6 (2 C, Ph), 122.3 (C-13), 121.33(C-6), 82.1 (C-1), 75.5 (C-8), 39.5 (C-7), 39.2 (C-9), 37.6 (C-10), 37.0 (C-4), 29.6 (C-3), 23.6 (C-14), 22.8 (C-2), 22.5 (C-15); ESI-MS: *m/z* 727.19 [2 M + Na]^+^; HRMS (ESI): *m/z* calcd for C_22_H_25_O_4_ [M + H]^+^ 353.17474, found 353.17480; HPLC: t_R_ = 12.4 min, purity = 97.3% @ 254 nm, 60% methanol in water.

*1β-carboxypropionyl alantolactone* (**1 h**)^34^. Yellow powder; HPLC: t_R_ = 23.1 min, purity = 95.4% @ 210 nm, 0–100% methanol in water for 50 min.

*1β-ethanoyl ivangustin* (**2a**). Cubic powder. Yield: 79% (18 mg); ${[{\rm{\alpha }}]}_{{\rm{D}}}^{30}$ = +83.4° (*c* 0.026 in CHCl_3_); ^1^H NMR (500 MHz, CDCl_3_): δ 6.27 (d, *J* = 2.9 Hz, 1 H, H-13a), 5.62 (d, *J* = 2.6 Hz, 1 H, H-13b), 4.77 (dd, *J* = 11.8, 8.2 Hz, 1 H, H-1), 4.50 (m, 1 H, H-8), 3.07 (m, 2 H, H-7), 2.84 (dd, *J* = 13.6, 7.4 Hz, 1 H, H-6b), 2.18 (dd, *J* = 13.9, 4.4 Hz, 1 H, H-9a), 2.08 (s, 3 H, C*H*_3_CO-1), 1.97–1.77 (m, 5 H, H-2b, H-3, H-6a, H-9b), 1.66 (s, 3 H, H-15), 1.50 (dd, *J* = 14.0, 11.0 Hz, 1 H, H-2a), 1.12 (s, 3 H, H-14); ^13^C NMR (125 MHz, CDCl_3_): δ 171.1 (C-12), 170.1 (CH_3_*C*O-1), 139.7 (C-11), 130.0 (C-5), 127.1 (C-4), 122.2 (C-13), 75.6 (C-1), 74.7 (C-8), 40.4 (C-7), 37.9 (C-10), 37.8 (C-9), 30.6 (C-3), 27.8 (C-2), 23.5 (*C*H_3_CO-1), 21.6 (C-6), 21.4 (C-15), 19.3 (C-14); ESI-MS: m/z 290.9 [M + H]^+^; HRMS (ESI): *m/z* calcd for C_17_H_23_O_4_ [M + H]^+^ 291.15909, found 291.15906; HPLC: t_R_ = 25.5 min, purity = 99.9% @ 320 nm, 50% methanol in water.

*1β-benzoyl ivangustin* (**2b**). White powder. Yield: 87% (22 mg); ${[{\rm{\alpha }}]}_{{\rm{D}}}^{30}$ = +126.9° (*c* 0.035 in CHCl_3_); ^1^H NMR (500 MHz, CDCl_3_): δ 8.12 (m, 1 H, Ph), 8.06 (m, 2 H, Ph), 7.48 (m, 2 H, Ph), 6.27 (d, *J* = 2.9 Hz, 1 H, H-13a), 5.62 (d, *J* = 2.55 Hz, 1 H, H-13b), 4.77 (dd, *J* = 11.8 Hz, 1 H, H-1), 4.50 (m, 1 H, H-8), 3.07 (m, 1 H, H-7), 2.84 (dd, 1 H, *J* *=* 13.6 Hz, H-6a), 2.18 (dd, 1 H, H-9a), 1.97 (t, *J* = 24.3 Hz, 1 H, H-3a), 1.91 (dd, *J* = 14.1 Hz, 1 H, H-9b), 1.89–1.77 (m, 3 H, H-2a, H-3b, H-6b), 1.66 (s, 3 H, H-15), 1.50 (dd, *J* = 14.0 Hz, 1 H, H-2b), 1.12 (s, 3 H, H-14); ^13^C NMR (125 MHz, CDCl_3_): δ 170.6 (C-12), 166.5 (Ph*C*OO-1), 139.8 (C-11), 133.8 (Ph), 133.2 (Ph), 130.3 (2 C, Ph), 129.5 (C-5), 128.6 (2 C, Ph), 127.3 (C-4), 122.2 (C-13), 75.6 (C-1), 75.5 (C-8), 40.4 (C-7), 38.3 (C-10), 37.9 (C-9), 30.7 (C-3), 27.8 (C-2), 23.6 (C-6), 21.8 (C-15), 19.1(C-14); ESI-MS: m/z 727.10 [2 M + Na]^+^; HRMS (ESI): *m/z* calcd for C_22_H_25_O_4_ [M + H]^+^ 353.17474, found 353.17480; HPLC: t_R_ = 34.0 min, purity = 95.3% @ 254 nm, 0–100% methanol in water for 50 min.

#### General procedure for the synthesis of 1c, 1e, 1d and 2c

To a suspension of compound **1** or **2** (0.1 mmol) in anhydrous pyridine (2 mL) in an ice-bath stirring for 30 min, substituted benzoyl chloride (0.2 mmol) was added. After completion of the reaction for 8 h at room temperature, ice water (2 mL) was added to the solvent and stirred for 20 min. Then the resultant solution was added diluted hydrochloric acid extracted with CH_2_Cl_2_, dried and filtered. After removal of the solvent, the crude product was purified by silica gel chromatography (EtOAc/PE) to afford compound **1c**, **1e**, **1d** and **2c**.

*1β-(4-fluorobenzoyl) alantolactone* (**1c**). White powder. Yield: 87% (21 mg); [α] = +158.6° (*c* 0.037 in CHCl_3_); ^1^H NMR (500 MHz, CDCl_3_): δ 8.07–8.04 (m, 2 H, *J* = 5.3 Hz, Ph), 7.15–7.10 (m, 2 H, *J* = 8.6 Hz, Ph), 6.28 (d, *J* = 1.8 Hz, 1 H, H-13a), 5.66 (d, *J* = 1.6 Hz, 1 H, H-13b), 5.32 (d, *J* = 3.9 Hz, 1 H, H-6), 4.79 (m, 2 H, H-1, H-8), 3.60 (m, 1 H, H-7), 2.51 (m, 1 H, H-4), 2.37 (m, *J* = 15 Hz, 1 H, H-9a), 2.04 (s, 1 H, H-2a), 1.82–1.74 (m, 2 H, H-2a, H-3b), 1.67–1.59 (m, 2 H, H-3b, H-9b), 1.40 (dd, *J* = 14.0 Hz, 3 H, H-14), 1.17 (s, 3 H, H-15); ^13^C NMR (125 MHz, CDCl_3_): δ 170.1 (C-12), 166.2 (*J*_C-F_ = 255.3 Hz, Ph), 165.3 (Ph), 147.05 (C-5), 139.5 (C-11), 132.3 (2 C, Ph), 126.7 (Ph), 122.3 (C-13), 121.4 (C-6), 115.7–115.9 (2 C, Ph), 82.3 (C-1), 75.4 (C-8), 39.5 (C-7), 39.3 (C-9), 37.6 (C-10), 37.2 (C-4), 29.6 (C-3), 23.6 (C-14), 22.8 (C-2), 22.5 (C-15); ESI-MS: *m/z* 331.4 [M + Na]^+^; HRMS (ESI): *m/z* calcd for C_22_H_24_FO_4_ [M + H]^+^ 371.16531, found 371.16528; HPLC: t_R_ = 14.2 min, purity = 97.6% @ 235 nm, 60% methanol in water.

*1β-(4-methoxybenzamide) alantolactone* (**1e**). White powder. Yield: 90% (26 mg); [α] = +158.0° (*c* 0.029 in CHCl_3_); ^1^H NMR (500 MHz, CDCl_3_): δ 8.00 (d, *J* = 8.9 Hz, 2 H, Ph), 6.94 (d, *J* = 8.9 Hz, 2 H, Ph), 6.22 (d, *J* = 1.8 Hz, 1 H, H-13a), 5.65 (d, *J* = 1.6 Hz, 1 H, H-13b), 5.30 (d, *J* = 3.8 Hz, 1 H, H-6), 4.80 (s, 1 H, H-8), 4.75 (dd, *J* = 11.7, 4.1 Hz, 1 H, H-1), 3.87 (s, 3 H, Ph-OC*H*_3_), 3.59 (s, 1 H, H-7), 2.57–2.44 (m, 1 H, H-4), 2.37 (dd, *J* = 15.0, 2.9 Hz, 1 H, H-9a), 2.00 (ddd, *J* = 17.2, 13.6, 4.0 Hz, 1 H, H-2a), 1.79 (d, *J* = 26.7 Hz, 2 H, H-2b, H-3a), 1.62 (dd, *J* = 15.0, 3.3 Hz, 2 H, H-9b, H-3b), 1.40 (s, 3 H, H-14), 1.16 (d, *J* = 7.6 Hz, 3 H, H-15); ^13^C NMR (125 MHz, CDCl_3_): δ 170.2 (C-12), 166.0 (Ph*C*OO-1), 163.7 (Ph), 147.3 (C-5), 139.6 (C-11), 131.8 (2 C, Ph), 122.8 (Ph), 122.2 (C-13), 121.3 (C-6), 113.9 (2 C, Ph), 81.7 (C-1), 75.5 (C-8), 55.6 (Ph-O*C*H_3_), 39.5 (C-7), 39.2 (C-9), 37.6 (C-10), 37.3 (C-4), 29.6 (C-3), 23.6 (C-14), 22.7 (C-2), 22.6 (C-15); ESI-MS: *m/z* 787.03 [2 M + Na]^+^; HRMS (ESI): *m/z* calcd for C_23_H_27_O_5_ [M + H]^+^ 383.18530, found 383.18533; HPLC: t_R_ = 14.1 min, purity = 99.0% @ 254 nm, 60% methanol in water.

*1β-(4-nitrobenzamide) alantolactone* (**1d**). White powder. Yield: 55% (12 mg); [α] = +159.7° (*c* 0.028 in CHCl_3_); ^1^H NMR (500 MHz, CDCl_3_): δ 8.31 (d, *J* = 8.9 Hz, 2 H, Ph), 8.21 (d, *J* = 8.9 Hz, 2 H, Ph), 6.24 (d, *J* = 1.8 Hz, 1 H, H-13a), 5.67 (d, *J* = 1.6 Hz, 1 H, H-13b), 5.33 (d, *J* = 4.0 Hz, 1 H, H-6), 4.86–4.76 (m, 2 H, H-1, H-8), 3.60 (d, *J* = 2.4 Hz, 1 H, H-7), 2.52 (s, 1 H, H-4), 2.35 (dd, *J* = 14.9, 2.9 Hz, 1 H, H-9a), 2.05 (dd, *J* = 12.4, 4.0 Hz, 1 H, H-2a), 1.83 (dd, *J* = 20.7, 7.4 Hz, 2 H, H-2b, H-3a), 1.68–1.61 (m, 2 H, H-9b, H-3b), 1.42 (s, 3 H, H-14), 1.17 (d, *J* = 7.6 Hz, 3 H, H-15).^13^C NMR (125 MHz, CDCl_3_): δ 170.3 (C-12), 164.6 (Ph*C*OO-1), 151.0 (Ph), 147.0 (C-5), 139.5 (C-11), 136.1 (Ph), 131.1 (2 C, Ph), 124.0 (2 C, Ph), 122.7 (C-13), 121.9 (C-6), 83.6 (C-1), 75.5 (C-8), 39.7 (C-7), 39.6 (C-9), 37.8 (C-10), 37.4 (C-4), 29.7 (C-3), 23.9 (C-14), 23.0 (C-2), 22.8 (C-15); ESI-MS: *m/z* 397.9 [M-H]^-^; HRMS (ESI): *m/z* calcd for C_22_H_24_NO_6_ [M + H]^+^ 398.15981, found 398.15970; HPLC: t_R_ = 13.4 min, purity = 98.6% @ 225 nm, 60% methanol in water.

*1β-(4-fluorobenzoyl) ivangustin* (**2c**). White powder. Yield: 45% (10 mg); [α] = +134.5° (*c* 0.013 in CHCl_3_); ^1^H NMR (500 MHz, CDCl_3_): 8.05 (m, *J* = 5.5 Hz, 2 H, Ph), 7.13 (m, *J* = 9.3 Hz, 2 H, Ph), 6.28 (d, *J* = 2.9 Hz, 1 H, H-13a), 5.64 (d, *J* = 2.5 Hz, 1 H, H-13b), 5.02 (dd, *J* = 11.8 7.4 Hz, 1 H, H-1), 4.55 (m, 1 H, H-8), 3.11 (m, 1 H, H-7), 2.87 (dd, 1 H, *J* = 13.7 6.2 Hz, H-6a), 2.27 (m, *J* = 2.9 Hz, 1 H, H-9a), 2.16 (t, 1 H, H-3a), 1.98 (m, 3 H, H-9b, H-2a, H-6b), 1.91 (dd, 1 H, H-3b), 1.70 (s, 3 H, H-15), 1.61 (dd, 1 H, H-2b), 1.25 (s, 3 H, H-14); ^13^C NMR (125 MHz, CDCl_3_): 170.49 (C-12), 165.5 (Ph*C*OO-1), 166.0 (*J*_C-F_ = 255.8 Hz, Ph), 139.8 (C-11), 132.3–132.2 (2 C, Ph), 129.9 (C-5), 127.3 (C-4), 126.8, (Ph), 122.2 (C-13), 115.8–115.7 (2 C, Ph), 75.7 (C-8), 75.5 (C-1), 40.4 (C-7), 38.3 (C-10), 37.9 (C-9), 30.7 (C-3), 27.8 (C-2), 23.6 (C-6), 21.8 (C-15), 19.1 (C-14); ESI-MS: *m/z* 370.84 [M + H]^+^; HRMS (ESI): *m/z* calcd for C_22_H_24_FO_4_ [M + H]^+^ 371.16531, found 371.16537; HPLC: t_R_ = 10.3 min, purity = 99.7% @ 230 nm, 60% methanol in water.

#### General procedure for the synthesis of 1f and 1g

To a suspension of derivatives of cinnamic acid (0.3 mmol), dicyclohexylcarbodiimide (DCC, 0.3 mmol) and DMAP (0.01 mmol) in anhydrous CH_2_Cl_2_ (1 mL) stirred for 20 min was added compound **1** or **2** (0.1 mmol) in anhydrous CH_2_Cl_2_ (1 mL) solution. After 4 h at 60 °C, the reaction completed and water (2 mL) was added to the solvent and stirred for 20 min, then extracted with CH_2_Cl_2_, dried and filtered. After removal of the solvent, the crude product was purified by silica gel chromatography (EtOAc/PE) to afford compounds **1 f** and **1 g**.

*1β-(4-bromocinnamoyl) alantolactone* (**1f**). White powder. Yield: 68% (16 mg); [α] = +210.2° (*c* 0.025 in CHCl_3_); ^1^H NMR (500 MHz, CDCl_3_): δ 7.60 (d, *J* = 16.0 Hz, 1 H, Ph-*C*H = CH-), 7.53 (d, *J* = 8.4 Hz, 2 H, Ph), 7.40 (d, *J* = 8.5 Hz, 2 H, Ph), 6.44 (d, *J* = 16.0 Hz, 1 H, Ph-CH = *C*H-), 6.22 (d, *J* = 1.8 Hz, 1 H, H-13a), 5.65 (d, *J* = 1.5 Hz, 1 H, H-13b), 5.29 (d, *J* = 3.9 Hz, 1 H, H-6), 4.81 (dt, *J* = 6.4, 3.1 Hz, 1 H, H-8), 4.67 (dd, *J* = 11.8, 4.0 Hz, 1 H, H-1), 3.62–3.54 (m, 1 H, H-7), 2.52–2.43 (m, 1 H, H-4), 2.34 (dd, *J* = 15.0, 2.9 Hz, 1 H, H-9a), 1.96 (dd, *J* = 11.5, 3.6 Hz, 1 H, H-2a), 1.74 (dd, *J* = 12.3, 5.0 Hz, 2 H, H-3a, H-2b), 1.60 (dd, *J* = 14.9, 3.2 Hz, 2 H, H-3b, H-9b), 1.33 (s, 3 H, H-15), 1.14 (d, *J* = 7.6 Hz, 3 H, H-14); ^13^C NMR (125 MHz, CDCl_3_): δ 170.2 (C-12), 166.4 (Ph*C*OO-1), 147.1 (C-5), 143.7 (Ph-*C*H = CH-), 139.5 (C-11), 133.3 (Ph), 132.3 (2 C, Ph), 129.6 (2 C, Ph), 124.8 (Ph), 122.3 (C-13), 121.3 (C-6), 119.0 (Ph-CH = *C*H-), 81.7 (C-1), 75.5 (C-8), 39.5 (C-7), 39.1 (C-9), 37.5 (C-10), 37.2 (C-4), 29.6 (C-3), 23.4 (C-14), 22.7 (C-2), 22.53 (C-15); ESI-MS: *m/z* 936.85 [2 M + Na]^+^; HRMS (ESI): *m/z* calcd for C_24_H_26_BrO_4_ [M + H]^+^ 457.10090, found 457.10117; HPLC: t_R_ = 35.4 min, purity = 97.5% @ 285 nm, 0–100% methanol in water for 50 min.

*1β-(4-trifluoromethyl) alantolactone* (**1g**). White powder. Yield: 53% (14 mg); [α] = +166.1° (*c* 0.035 in CHCl_3_); ^1^H NMR (500 MHz, CDCl_3_) δ 7.69–7.63 (m, 5 H, H-20, H-21, H-18), 6.53 (d, *J* = 16.0 Hz, 1 H, H-17), 6.23 (d, *J* = 1.4 Hz, 1 H, H-13a), 5.66 (d, *J* = 1.1 Hz, 1 H, H-13b), 5.30 (d, *J* = 3.9 Hz, 1 H, H-6a), 4.82 (dt, *J* = 6.3, 3.0 Hz, 1 H, H-1), 4.69 (dd, *J* = 11.8, 3.9 Hz, 1 H, H-8), 3.62–3.56 (m, 1 H, H-7), 2.54–2.45 (m, 1 H, H-4), 2.35 (dd, *J* = 15.0, 2.8 Hz, 1 H, H-9a), 1.98 (dd, *J* = 11.7, 3.4 Hz, 1 H, H-2a), 1.76 (dd, *J* = 5.4, 3.6 Hz, 2 H, H-6b, H-2b), 1.61 (dd, *J* = 14.9, 3.0 Hz, 2 H, H-9b, H-3b), 1.34 (s, 3 H, H-15), 1.14 (d, *J* = 7.6 Hz, 3 H, H-14); ^13^C NMR (125 MHz, CDCl_3_) δ 170.20 (C-12), 166.14 (Ph*C*OO-1), 147.02 (C-5), 143.24 (Ph-*C*H = CH-), 139.42 (C-11), 137.78 (Ph), 128.39 (2 C, Ph), 128.15 (Ph), 126.05–126.02 (2 C, Ph), 122.46 (Ph-*C*F_3_), 122.33 (C-13), 121.35 (C-6), 120.90 (Ph-CH = *C*H-), 81.91 (C-1), 75.45 (C-8), 39.46 (C-7), 39.10 (C-9), 37.46 (C-10), 37.19 (C-3), 23.46 (C-14), 22.74 (C-2), 22.53 (C-15); ESI-MS: *m/z* 447.3 [M + H]^+^; HRMS (ESI): *m/z* calcd for C_25_H_25_F_3_O_4_ [M + H]^+^ 447.17777, found 447.17770; HPLC: t_R_ = 36.5 min, purity = 95.8% @ 285 nm, 0–100% methanol in water for 50 min.

#### General procedure for the synthesis of 1i and 2d

To a suspension of Dess-Martin periodinane (0.4 mmol) in anhydrous CH_2_Cl_2_ (1 mL) was added compound **1** or **2** (0.2 mmol) and water (0.1 mmol) in anhydrous CH_2_Cl_2_ (1 mL) solution^34^. The resultant solution was added saturated aqueous NaHCO_3_ and extracted with CH_2_Cl_2_. After removal of the solvent, the crude product was purified by silica gel chromatography (EtOAc/PE) to afford compound **1i** and **2d**.

*1-carbonyl-alantolactone* (**1i**)^34^. White powder; HPLC: t_R_ = 10.2 min, purity = 97.0% @ 230 nm, 50% methanol in water.

*1-carbonyl-ivangustin* (**2d**). White powder. Yield: 76% (18 mg); [α] = +79.1° (*c* 0.028 in CHCl_3_); ^1^H NMR (500 MHz, CDCl_3_): δ 6.29 (d, *J* = 2.8 Hz, 1 H, H-13a), 5.63 (d, *J* = 2.5 Hz, 1 H, H-13b), 4.41 (m, 1 H, H-8), 2.99 (m, 1 H, H-7), 2.87 (dd, *J* = 13.7, 6.5 Hz, 1 H, H-6a), 2.69 (dd, 1 H, H-6b), 2.60 (t, *J* = 12.1 Hz, 2 H, H-3), 2.33 (m, 2 H, H-2), 2.05 (dd, *J* = 12.2 Hz, 1 H, H-9a), 1.80 (s, 3 H, H-15), 1.54 (m, *J* = 13.8, 3.3 Hz, 1 H, H-9b), 1.22 (s, 3 H, H-14); ^13^C NMR (125 MHz, CDCl_3_): δ 212.6 (C-1), 170.3 (C-12), 139.5 (C-11), 130.2 (C-5), 129.1 (C-4), 122.5 (C-13), 75.6 (C-8), 47.2 (C-10), 39.4 (C-9), 34.8 (C-7), 30.1 (C-2), 29.8 (C-3), 28.4 (C-6), 24.1 (C-15), 19.6 (C-14); ESI-MS: *m/z* 247.01 [M + H]^+^; HRMS (ESI): *m/z* calcd for C_15_H_19_O_3_ [M + H]^+^ 247.13287, found 247.13275; HPLC: t_R_ = 5.8 min, purity = 98.6% @ 254 nm, 60% methanol in water.

#### General procedure for the synthesis of 1j

To a solution of compound **1b** (0.01 mmol) in acetic acid was added Mn(OAc)_3_·2H_2_O (0.35 mmol) and KOAc (2.9 mmol). The resultant solution was stirred and refluxed for 3 h. TLC analysis of the reaction shows a complete disappearance of the starting material. Ethyl acetate (5 mL) and water (5 mL) were added to the solution, then saturated aqueous NaHCO_3_ was used to adjust the solution to pH = 7 and extracted with CH_2_Cl_2_. After removal of the solvent, the crude product was purified by silica gel chromatography (EtOAc/PE) to afford compound **1j**.

*1β-benzoyl alanto-spirobislactone* (**1j**). Cubic crystal. Yield: 76% (18 mg); ${[{\rm{\alpha }}]}_{{\rm{D}}}^{30}$ = +74.1° (c = 0.026 in CHCl_3_); The data of ^1^H and ^13^C NMR were shown in Table 1; ESI-MS: *m/z* 247.01 [M + H]^+^; HRMS (ESI): *m/z* calcd for C_24_H_27_O_6_ [M + H]^+^ 411.18022, found 411.18024; HPLC: t_R_ = 32.8 min, purity = 99.0% @ 230 nm, 0–100% methanol in water for 50 min.

#### General procedure for the synthesis of C13-methylene modified spiro[lactone-isoxazol] (1k–q)

To a stirred solution of benzaldehyde derivatives (0.01 mol) in diethyl ether (10 mL) was added 50% hydroxylamine in H_2_O (0.01 mol) in one portion^34^. The reaction mixture immediately became warm and was stirred for a further 10 min, then dried (MgSO_4_) and filtered, and the ether was removed under reduced pressure to yield benzaldoxime derivatives. To a colorless, homogeneous solution of benzaldoxime derivatives (5 mmol) in *N*,*N*-dimethylformamide (DMF, 8 mL) at room temperature was added *N*-chlorosuccinimide (NCS, 5 mmol) portion-wise over 30 min. After the addition was complete, the homogeneous reaction mixture was stirred overnight at room temperature. The reaction mixture was diluted with 50 mL of water and extracted with ether (3 × 18 mL). The organic layers were combined, washed with water (2 × 15 mL), washed with a 10 percent aqueous solution of sodium chloride (2 × 15 mL), washed with brine (15 mL), and dried over anhydrous sodium sulfate. Concentration under reduced pressure afforded benzaldoxime chloride (4.9 mmol, 98 percent yield) as a fluffy, pale yellow solid.

To a solution of benzaldoxime chloride (0.105 mmol) and **1i** (0.1 mmol) in CH_2_Cl_2_ (3 mL) was added Et_3_N (0.125 mmol) at 0 °C. The resulting mixture was stirred at room temperature for 12 h. The solvent was evaporated in vacuo and the residue was purified via silica column chromatography with (EtOAc/PE) as eluent to provide compounds **1k**–**q**.

*(11* *S)-16-(p-methylphenyl)-spiroisoxazoline-1-carbonyl-alantolactone* (**1k**)^34^. White powder; HPLC: t_R_ = 39.0 min, purity = 95.1% @ 270 nm, 0–100% methanol in water for 50 min.

*(11* *S)-16-(p-nitrophenyl)-spiroisoxazoline-1-carbonyl-alantolactone* (**1l**). Yellow powder. Yield: 96% (28 mg); ${[{\rm{\alpha }}]}_{{\rm{D}}}^{30}$ = +285.2° (*c* 0.33 in CHCl_3_); ^1^H NMR (500 MHz, CDCl_3_) δ 8.31–8.24 (m, 2 H, H-18, H-18′), 7.92–7.84 (m, 2 H, H-19, H-19′), 5.34 (d, *J* = 3.3 Hz, 1 H, H-6), 5.22 (m, *J* = 5.3, 2.8 Hz, 1 H, H-8), 3.71 (d, *J* = 17.0 Hz, 1 H, H-13a), 3.55 (d, *J* = 17.0 Hz, 1 H, H-13b), 3.16–3.10 (m, 1 H, H-7), 2.78–2.67 (m, 2 H, H-2a, H-4), 2.54 (dd, *J* = 15.6, 3.6 Hz, 1 H, H-9a), 2.25 (m, *J* = 16.2, 9.3, 4.9 Hz, 1 H, H-2b), 2.05–1.95 (m, 1 H, H-3a), 1.93 (dd, *J* = 15.7, 2.6 Hz, 1 H, H-9b), 1.84–1.75 (m, 1 H, H-3b), 1.44 (s, 3 H, H-14), 1.30 (d, *J* = 7.3 Hz, 3 H, H-15); ^13^C NMR (125 MHz, CDCl_3_): δ 212.2 (C-1), 172.5 (C-12), 155.0 (C-16), 150.6 (C-5), 148.9 (C-20), 134.5 (C-17), 127.8 (C-18, C-18′), 124.1 (C-19, C-19′), 115.5 (C-6), 90.8 (C-11), 76.6 (C-8), 47.2 (C-10), 42.7 (C-7), 36.6 (C-13), 36.3 (C-4), 35.5 (C-2), 33.7 (C-9), 28.1 (C-3), 28.1 (C-14), 23.7 (C-15); HRMS (ESI): *m/z* calcd for C_22_H_23_N_2_O_6_ [M + H]^+^ 411.15506, found 411.15515; HPLC: t_R_ = 38.6 min, purity = 97.0% @ 299 nm, 0–100% methanol in water for 50 min.

*(11* *S)-16-(o-nitrophenyl)-spiroisoxazoline-1-carbonyl-alantolactone* (**1m**). Yellow powder. Yield: 59% (14 mg); ${[{\rm{\alpha }}]}_{{\rm{D}}}^{30}$ = + 88.5° (*c* 0.22 in MeOH); ^1^H NMR (500 MHz, CDCl_3_) δ 8.19 (dd, *J* = 8.2, 1.0 Hz, 1 H, H-20), 7.76 (dd, *J* = 7.5, 1.1 Hz, 1 H, H-19′), 7.69–7.64 (m, 2 H, H-18, H-19), 5.43 (d, *J* = 3.4 Hz, 1 H, H-6), 5.22 (m, *J* = 5.4, 2.9 Hz, 1 H, H-8), 3.60 (d, *J* = 17.1 Hz, 1 H, H-13a), 3.50 (d, *J* = 17.0 Hz, 1 H, H-13b), 3.27–3.21 (m, 1 H, H-7), 2.72 (m, *J* = 12.9, 7.9, 4.6 Hz, 2 H, H-2a, H-4), 2.54 (dd, *J* = 15.7, 3.6 Hz, 1 H, H-9a), 2.28–2.21 (m, 1 H, H-2b), 2.02–1.94 (m, 1 H, H-3a), 1.92 (dd, *J* = 15.7, 2.6 Hz, 1 H, H-9b), 1.80–1.73 (m, 1 H, H-3b), 1.42 (s, 3 H, H-14), 1.24 (d, *J* = 7.3 Hz, 3 H, H-15); ^13^C NMR (125 MHz, CDCl_3_): δ 212.6 (C-1), 172.7 (C-12), 155.9 (C-16), 150.0 (C-5), 147.8 (C-17), 134.2 (C-18′), 131.6 (C-20), 131.2 (C-19′), 125.1 (C-19), 124.9 (C-18), 116.0 (C-6), 90.8 (C-11), 76.8 (C-8), 47.1 (C-10), 43.0 (C-7), 39.8 (C-13), 36.5 (C-4), 35.6 (C-2), 33.9 (C-9), 28.1 (C-3), 28.1 (C-14), 23.6 (C-15); HRMS (ESI): *m/z* calcd for C_22_H_23_N_2_O_6_ [M + H]^+^ 411.15506, found 411.15530; HPLC: t_R_ = 43.0 min, purity = 95.9% @ 212 nm, 0–100% methanol in water for 50 min.

*(11* *S)-16-(p-chlorophenyl)-spiroisoxazoline-1-carbonyl-alantolactone* (**1n**). White powder. Yield: 61% (15 mg); ${[{\rm{\alpha }}]}_{{\rm{D}}}^{30}$ = +260.8° (*c* 0.22 in MeOH); ^1^H NMR (500 MHz, CDCl_3_) δ 7.67–7.61 (m, 2 H, H-19, H-19′), 7.45–7.36 (m, 2 H, H-18, H-18′), 5.33 (d, *J* = 3.3 Hz, 1 H, H-6), 5.20 (m, *J* = 5.4, 2.9 Hz, 1 H, H-8), 3.66 (d, *J* = 17.0 Hz, 1 H, H-13a), 3.48 (d, *J* = 17.0 Hz, 1 H, H-13b), 3.11–3.08 (m, 1 H, H-7), 2.73 (m, *J* = 15.5, 9.7, 5.9 Hz, 2 H, H-2a, H-4), 2.54 (dd, *J* = 15.6, 3.6 Hz, 1 H, H-9a), 2.29–2.20 (m, 1 H, H-2b), 2.05–1.94 (m, 1 H, H-3a), 1.91 (dd, *J* = 15.7, 2.6 Hz, 1 H, H-9b), 1.83–1.74 (m, 1 H, H-3b), 1.44 (s, 3 H, H-14), 1.30 (d, *J* = 7.3 Hz, 3 H, H-15); ^13^C NMR (125 MHz, CDCl_3_): δ 212.4 (C-1), 172.8 (C-12), 155.5 (C-16), 150.2 (C-5), 136.9 (C-20), 129.2 (C-17), 128.2 (C-19, C-19′), 127.0 (C-18, C-18′), 115.9 (C-6), 90.0 (C-11), 76.5 (C-8), 47.2 (C-10), 42.9 (C-7), 36.7 (C-13), 36.6 (C-4), 35.5 (C-2), 33.8 (C-9), 28.2 (C-3), 28.1 (C-14), 23.7 (C-15); HRMS (ESI): *m/z* calcd for C_22_H_23_ClNO_4_ [M + H]^+^ 400.13101, found 400.13110; HPLC: t_R_ = 39.8 min, purity = 95.3% @ 269 nm, 0–100% methanol in water for 50 min.

*(11* *S)-16-(p-methoxyphenyl)-spiroisoxazoline-1-carbonyl-alantolactone* (**1o**). White powder. Yield: 61% (15 mg); ${[{\rm{\alpha }}]}_{{\rm{D}}}^{30}$ = +86.3° (*c* 0.31 in MeOH); The data of ^1^H and ^13^C NMR were shown in Table 1; HRMS (ESI): *m/z* calcd for C_23_H_26_NO_5_ [M + H]^+^ 396.18055, found 396.18055; calcd for C_46_H_51_N_2_O_10_ [2 M + H]^+^ 791.35382, found 791.35345; HPLC: t_R_ = 39.7 min, purity = 95.5% @ 278 nm, 0–100% methanol in water for 50 min.

*(11* *S)-16-(5-chloro-2-methoxylphenyl)-spiroisoxazoline-1-carbonyl-alantolactone* (**1p**). White powder. Yield: 48% (13 mg); ${[{\rm{\alpha }}]}_{{\rm{D}}}^{30}$ = +92.1° (*c* 0.23 in MeOH); ^1^H NMR (500 MHz, CDCl_3_) δ 7.74 (d, *J* = 2.6 Hz, 1 H, H-18), 7.36 (dd, *J* = 8.9, 2.7 Hz, 1 H, H-20), 6.90 (d, *J* = 8.9 Hz, 1 H, H-19′), 5.33 (d, *J* = 3.4 Hz, 1 H, H-6), 5.18 (m, *J* = 5.4, 2.9 Hz, 1 H, H-8), 3.89 (s, 3 H, H-18′-OMe), 3.77 (d, *J* = 17.8 Hz, 1 H, H-13a), 3.60 (d, *J* = 17.8 Hz, 1 H, H-13b), 3.10–3.04 (m, 1 H, H-7), 2.77–2.66 (m, 2 H, H-2a, H-4), 2.52 (dd, *J* = 15.6, 3.6 Hz, 1 H, H-9a), 2.27–2.19 (m, 1 H, H-2b), 2.02–1.95 (m, 1 H, H-3a), 1.91 (dd, *J* = 15.7, 2.7 Hz, 1 H, H-9b), 1.79 (m, *J* = 13.9, 9.4, 7.3, 4.6 Hz, 1 H, H-3b), 1.43 (s, 3 H, H-14), 1.29 (d, *J* = 7.3 Hz, 3 H, H-15); ^13^C NMR (125 MHz, CDCl_3_): δ 212.4 (C-1), 173.0 (C-12), 156.2 (C-16), 155.0 (C-18′), 149.8 (C-5), 131.5 (C-20), 129.3 (C-18), 126.2 (C-19), 119.0 (C-17), 116.3 (C-6), 112.9 (C-19′), 89.9 (C-11), 76.3 (C-8), 56.1 (C-18′-OMe), 47.2 (C-10), 43.0 (C-7), 39.1 (C-13), 36.6 (C-4), 35.6 (C-2), 33.8 (C-9), 29.7 (C-3), 28.1 (C-14), 23.6 (C-15); HRMS (ESI): *m/z* calcd for C_23_H_25_ClNO_5_ [M + H]^+^ 430.14158, found 430.14139; HPLC: t_R_ = 40.0 min, purity = 96.0% @ 230 nm, 0–100% methanol in water for 50 min.

*(11* *S)-16-(2-chloro-3,4,5-trimethoxylphenyl)-spiroisoxazoline-1-carbonyl-alantolactone* (**1q**). White powder. Yield: 26% (10 mg); ${[{\rm{\alpha }}]}_{{\rm{D}}}^{30}$ = +89.8° (*c* 0.25 in MeOH); ^1^H NMR (500 MHz, CDCl_3_) δ 7.01 (s, 1 H, H-18), 5.37 (d, *J* = 3.4 Hz, 1 H, H-6), 5.22–5.16 (m, 1 H, H-8), 3.93 (d, *J* = 4.4 Hz, 6 H, H-19-OMe, H-19′-OMe), 3.89 (s, 3 H, H-20-OMe), 3.81 (d, *J* = 17.5 Hz, 1 H, H-13a), 3.72 (d, *J* = 17.5 Hz, 1 H, H-13b), 3.16–3.12 (m, 1 H, H-7), 2.72 (m, *J* = 12.4, 8.1, 3.8 Hz, 2 H, H-2a, H-4), 2.52 (dd, *J* = 15.6, 3.7 Hz, 1 H, H-9a), 2.28–2.21 (m, 1 H, H-2b), 1.99 (d, *J* = 5.3 Hz, 1 H, H-3a), 1.93 (dd, *J* = 15.6, 2.7 Hz, 1 H, H-9b), 1.82–1.75 (m, 1 H, H-3b), 1.43 (s, 3 H, H-14), 1.28 (d, *J* = 7.3 Hz, 3 H, H-15); ^13^C NMR (125 MHz, CDCl_3_): δ 212.4 (C-1), 172.8 (C-12), 156.8 (C-16), 152.4 (C-19′), 150.5 (C-19), 150.0 (C-5), 145.2 (C-20), 123.1 (C-17), 116.2 (C-6), 119.6 (C-18′), 108.9 (C-18), 90.5 (C-11), 76.4 (C-8), 61.3 (C-19′-OMe), 61.2 (C-20-OMe), 56.3 (C-19-OMe), 47.2 (C-10), 42.9 (C-7), 39.4 (C-13), 36.5 (C-4), 35.6 (C-2), 33.9 (C-9), 29.7 (C-3), 28.1 (C-14), 23.6 (C-15); HRMS (ESI): *m/z* calcd for C_25_H_29_ClNO_7_ [M + H]^+^ 490.16271, found 490.16278; HPLC: t_R_ = 39.7 min, purity = 95.2% @ 226 nm, 0–100% methanol in water for 50 min.

#### General procedure for the synthesis of C13-methylene reductive derivatives 1r and 1s

NaBH_4_ (1.2 mmol) was added to a solution of 1β-hydroxy alantolactone (**1**) (0.3 mmol) in anhydrous THF (5 mL)^34^. The solution was stirred vigorously. The reaction was completed after ~2 h using TLC detection, 1 M HCl (2 mL) solution was added to quench the reaction (*caution, gas evolved*). The mixture was extracted with CH_2_Cl_2_, washed with brine, dried over anhydrous Na_2_SO_4_ and concentrated under reduced pressure. The residue was purified via silica column chromatography with EtOAc/PE (3:1) as eluent to provide compounds **1r** and **1 s** (87%; **1r**/**1 s** = 3.35/1, determined by isolation) as white solid.

*(11* *S)-1*β*-hydroxy alantolactone derivative* (**1r**)^34^. White powder; HPLC: t_R_ = 34.4 min, purity = 98.5% @ 210 nm, 0–100% methanol in water for 50 min.

*(11* *R)-1*β*-hydroxy alantolactone derivative* (**1 s**). White powder. Yield: 20% (5 mg); ${[{\rm{\alpha }}]}_{{\rm{D}}}^{30}$ = +2.8° (*c* 0.21 in MeOH); ^1^H NMR (500 MHz, CDCl_3_) δ 5.26 (d, *J* = 3.4 Hz, 1 H, H-6), 4.91 (dt, *J* = 6.3, 3.2 Hz, 1 H, H-8), 3.28 (dd, *J* = 11.6, 4.0 Hz, 1 H, H-1), 2.65–2.59 (m, 1 H, H-11), 2.50 (dd, *J* = 14.9, 3.8 Hz, 1 H, H-9b), 2.43 (m, *J* = 17.3, 8.9, 7.5 Hz, 2 H, H-4, H-7), 1.87–1.81 (m, 1 H, H-2b), 1.60–1.53 (m, 4 H, H-2a, H-3, H-9a), 1.35 (d, *J* = 7.6 Hz, 3 H, H-13), 1.22 (s, 3 H, H-14), 1.12 (d, *J* = 7.6 Hz, 3 H, H-15); ^13^C NMR (125 MHz, CDCl_3_) δ 179.8 (C-12), 147.8 (C-5), 121.5 (C-6), 80.4 (C-1), 76.0 (C-8), 43.6 (C-11), 42.4 (C-7), 39.4 (C-9), 38.6 (C-10), 37.8 (C-4), 30.0 (C-3), 26.2 (C-2), 22.5 (C-14), 22.0 (C-15), 16.1 (C-13); ESI-MS: *m/z* 522.97 [2 M + Na]^+^; HPLC: t_R_ = 34.4 min, purity = 98.3% @ 210 nm, 0–100% methanol in water for 50 min.

#### X-ray experimental

Crystals of derivatives **1a** and **1j** were obtained by solvent volatilization in C_2_H_5_OH/CH_2_Cl_2_. A suitable single crystal was selected and analysed on a SuperNova, Dual, Cu at zero, Eos diffractometer. The crystal was kept at 293(2) K during data collection. Using Olex2^53^, the structure was solved with the Superflip^54^ structure solution program using Charge Flipping and refined with the ShelXL^55^ refinement package using Least Squares minimisation.

### Biological activities

#### Cell Culture

PC-3 (human prostate cancer), HepG2 (human liver cancer) and CHO (Chinese hamster ovary) cell lines were obtained from Shanghai Institute of Biochemistry and Cell Biology, Chinese Academy of Sciences. HeLa (human cervix cancer), HUVEC (human umbilical vein endothelial cells) and HEp-2 (human laryngeal cancer) cell lines were granted by Prof. Lei of college of life sciences, Northwest A&F University. The six cell lines were grown in RPMI-1640 (Gibco) containing 10% (v/v) thermally inactivated fetal bovine serum (FBS), penicillin (100 KU/L) and streptomycin (100 KU/L) at 37 °C in a 5% CO_2_ humidified incubator.

#### Cytotoxic activity (SRB)

*In vitro* cytotoxicity was assessed by using the SRB colorimetric assay refer to previous methods^19,42^.

#### Nuclear chromatin condensation

Condensation of nuclear chromatin is usually the late apoptotic event and is detected by staining Hoechst 33258^43^. PC-3 cells containing 2.0 × 10^5^ cells/well were cultured on coverslips, and kept in six-well plates for 12 h. After 72 h treatment with **1i** and VP-16, Hoechst 33258 staining was carried out according to the kit’s procedure (Beyotime Institute Biotechnology, China). The cells were viewed under a fluorescence microscopy (Olympus BX53 + DP72) with a ×20 objective lens.

#### Cell cycle analysis

The cell cycle arrest on PC-3 and HepG2 cells was detected with PI staining assay (Sigma) as described previously^19^. After treatment with samples at the set concentrations for 48 h, cells were centrifuged and fixed in 70% ethanol at 4 °C refrigerator 12 h and then resuspended in PBS (100 μL RNase A and 400 μL PI). Cellular DNA content was measured using a FACSCalibur flow cytometer with Modfit LT 3.0 software. Twenty thousand cells were collected per sample. Mean values are presented from three independent experiments.

#### Cell transfection and NF-κB luciferase activity assay

PC-3 cells (8000 cells/well) were placed in a 96-well plate and the cells were then transfected with pNF-κB-Luc expression plasmid reference to the method^34^.

#### Western blotting analysis

PC-3 cells were treated with the indicated concentrations (1, 2 and 4 µM) of **1i** for 72 h, cell were collected and lysed. The protein concentration was measured by BCA method, and equal amount proteins were electrophoresed on 10% SDS-PAGE gel, electrotransferred onto NC membrane, and incubated with appropriate primary and secondary antibodies, protein blots were tested by ECL solution and ChemiDoc XRS+ imaging system (Bio-Rad, USA).

#### Molecular modeling

Docking and scoring studies of the interaction of compounds with p65/NF-κB were performed with Surflex-Dock protocol in Sybyl-X 2.1.1 software^34,56^. The structure of NF-κB p50−p65 heterodimer bound to DNA (1VKX) was obtained from the Protein Data Bank.

#### Statistics analysis

All the data reported were the arithmetic mean of data of independent experiments performed in triplicate where each group was three in number. Results shown were the mean ± standard deviation (SD). The 2-tailed Student t-test using GraphPad software performed statistical analysis.

## Electronic supplementary material

Supplementary information
