# Supplementary material for: Diversity Modification and Structure-Activity Relationships of Two Natural Products 1β-hydroxy Alantolactone and Ivangustin as Potent Cytotoxic Agents
Source: Sci Rep. 2018 Jan 29;8:1722. doi: 10.1038/s41598-018-20192-9 (PMC5789092; doi:10.1038/s41598-018-20192-9)
Supplement: Supplementary file 1 — Supplementary information [file 41598_2018_20192_MOESM1_ESM.docx]

***Supporting Information***

Diversity Modification and Structure-Activity Relationships of Two Natural Products 1β-hydroxy Alantolactone and Ivangustin as Potent Cytotoxic Agents

Jiang-Jiang Tang^1,*^, Qiu-Rui He^1^, Shuai Dong^1^, Xin Guo^1^, Yu-Gong Wang^1^, Bei-Lei Lei^2^, Jun-Mian Tian^1^, Jin-Ming Gao^1,*^

^1^Shaanxi Key Laboratory of Natural Products & Chemical Biology, College of Chemistry & Pharmacy, Northwest A&F University, Yangling, 712100, China

^2^College of Life Sciences, Northwest A&F University, Yangling, 712100, China

^*^Corresponding authors.

E-mail address: tangjiang11@nwafu.edu.cn (JJT); jinminggao@nwsuaf.edu.cn (JMG).

**List of Contents:**

Page S2. **Table S1**. Crystallographic parameters for **1a**.

Page S3. **Table S2**. Crystallographic parameters for **1j**.

Page S4. **Figure S1**. Synthetic mechanism of the desired spirobislactone **1j**.

Pages S4. **Figure S2**. Induction of apoptosis by active compounds **1** and **1i** on HepG2 cells.

Pages S5-S27. 1D or 2D NMR spectra.

Pages S28-S36. (HR)-ESI-MS spectra.

Pages S37-S43. HPLC purity determination.

**Table S1. Crystal data and structure refinement for 1a.**

| Identification code | **1a** |
| --- | --- |
| Empirical formula | C_17_H_22_O_4_ |
| Formula weight | 290.35 |
| Temperature/K | 293.25(10) |
| Crystal system | orthorhombic |
| Space group | P2_1_2_1_2_1_ |
| a/Å | 7.4266(7) |
| b/Å | 12.6128(12) |
| c/Å | 16.8409(16) |
| α/° | 90.00 |
| β/° | 90.00 |
| γ/° | 90.00 |
| Volume/Å^3^ | 1577.5(3) |
| Z | 8 |
| ρ_calc_mg/mm^3^ | 2.445 |
| m/mm^‑1^ | 1.399 |
| F(000) | 1248.0 |
| Crystal size/mm^3^ | 0.27 × 0.24 × 0.12 |
| Radiation | ? (λ = 1.54000) |
| 2Θ range for data collection | 10.5 to 141.04° |
| Index ranges | -9 ≤ h ≤ 8, -15 ≤ k ≤ 15, -20 ≤ l ≤ 15 |
| Reflections collected | 7190 |
| Independent reflections | 2975 [R_int_ = 0.0353, R_sigma_ = 0.0404] |
| Data/restraints/parameters | 2975/0/194 |
| Goodness-of-fit on F^2^ | 1.054 |
| Final R indexes [I>=2σ (I)] | R_1_ = 0.0449, wR_2_ = 0.1215 |
| Final R indexes [all data] | R_1_ = 0.0472, wR_2_ = 0.1254 |
| Largest diff. peak/hole / e Å^-3^ | 0.17/-0.15 |
| Flack parameter | -0.1(3) |

**Table S2. Crystal data and structure refinement for 1j.**

| Identification code | K1-17 |
| --- | --- |
| Empirical formula | C_24_H_26_O_6_ |
| Formula weight | 410.45 |
| Temperature/K | 293 (2) |
| Crystal system | tetragonal |
| Space group | P4(1) |
| a/Å | 13.1463(8) |
| b/Å | 13.1463(8) |
| c/Å | 12.732(7) |
| α/° | 90.00 |
| β/° | 90.00 |
| γ/° | 90.00 |
| Volume/Å^3^ | 2200.5(12) |
| Z | 4 |
| m/mm^‑1^ | 1.239 |
| F(000) | 872 |
| Crystal size/mm^3^ | 0.34 × 0.15 × 0.14 |
| Radiation | ? (λ = 1.54178) |
| 2Θ range for data collection | 3.36 to 66.18° |
| Index ranges | -14 ≤ h ≤ 10, -14 ≤ k ≤ 15, -9 ≤ l ≤ 15 |
| Reflections collected | 3957 |
| Independent reflections | 2575 [R_int_ = 0.0467, R_sigma_ = 0.0845] |
| Data/restraints/parameters | 2575/1/274 |
| Goodness-of-fit on F^2^ | 0.973 |
| Final R indexes [I>=2σ (I)] | R_1_ = 0.0541, wR_2_ = 0.1188 |
| Final R indexes [all data] | R_1_ = 0.0593, wR_2_ = 0.0774 |
| Largest diff. peak/hole / e Å^-3^ | 0.139/-0.137 |
| Flack parameter | -0.1(4) |

**Figure S1. Synthesis of the desired spirobislactone 1j. On the less hindered face, the complexed free-radical A approached α-methylene lactone 1b and the resultant radical B underwent lactonization to provide 1j in a substrate-controlled manner.**


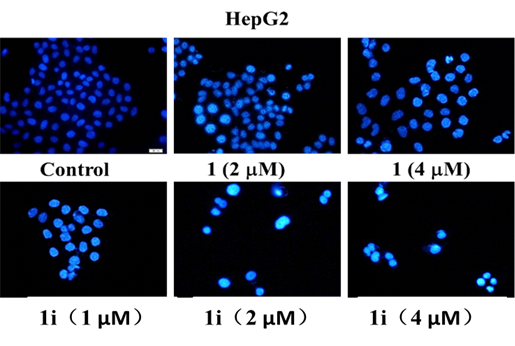


**Figure S2**. **Induction of apoptosis by active compounds 1 and** **1i at the indicated concentrations on HepG2 cells. 72 h after the treatment of these compounds at the indicated concentrations, cells were fixed, washed with PBS, stained with Hoechst 33258, and analyzed for morphological characteristics associated with apoptosis by fluorescence microscopic analysis (20×).** **NMR spectra**


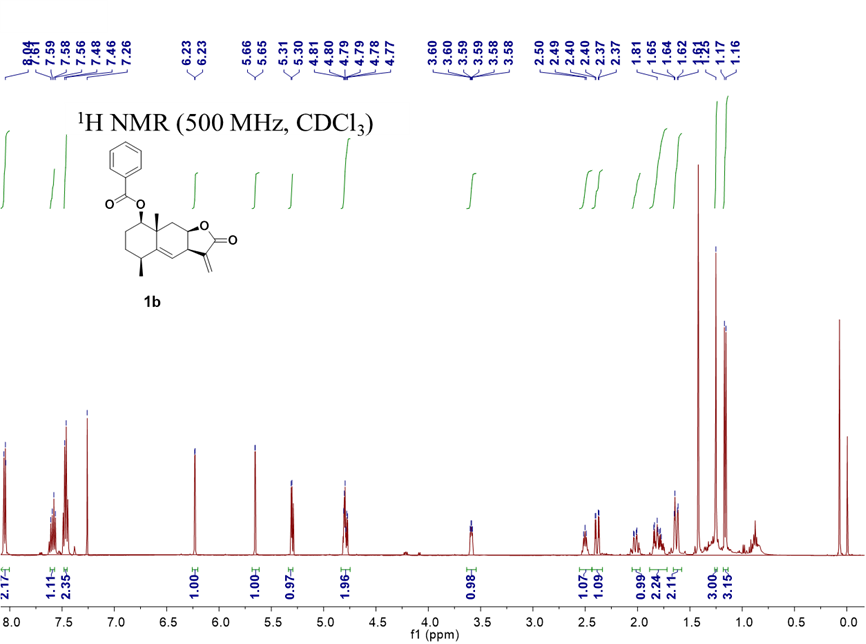

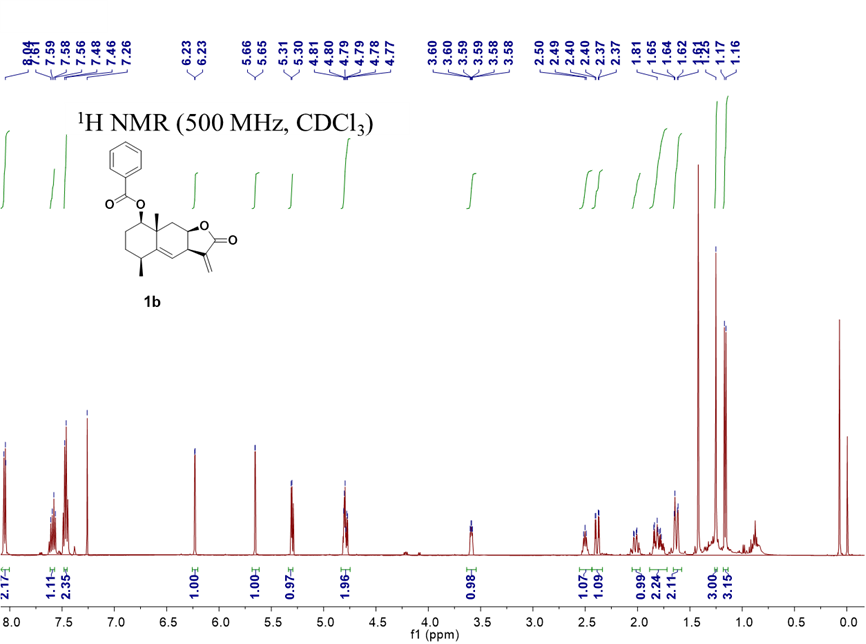

^1^H NMR (125 Hz, CDCl_3_)


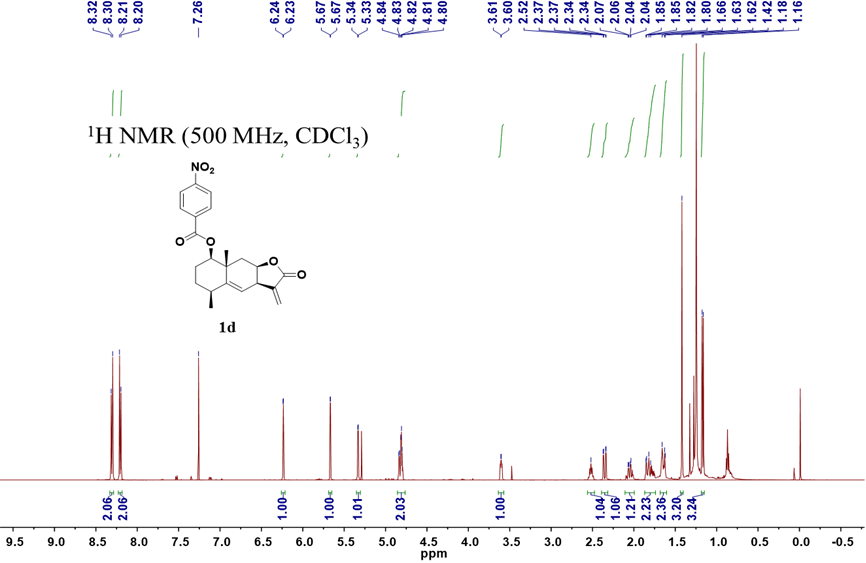


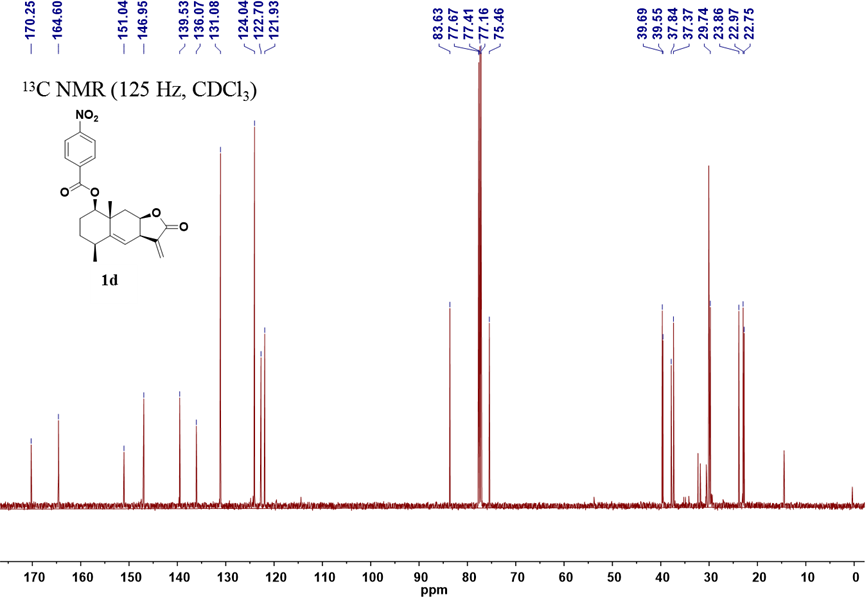


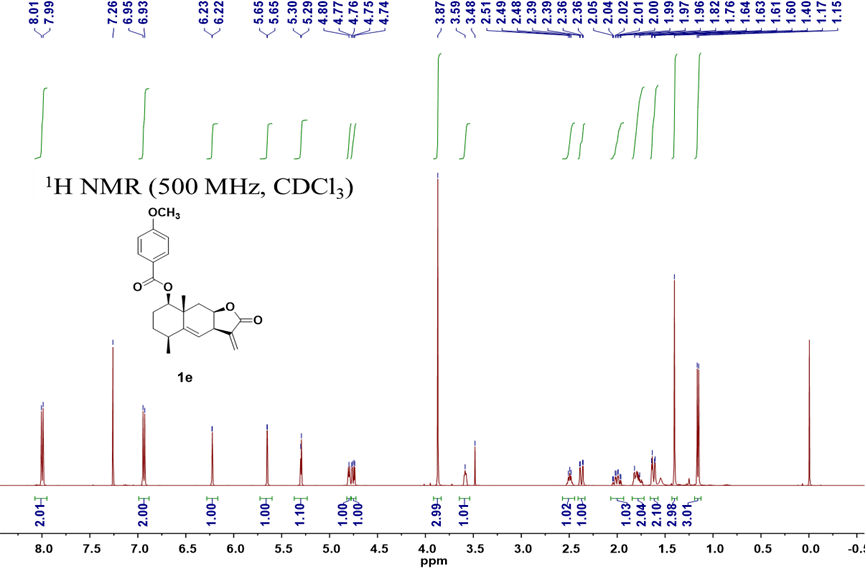

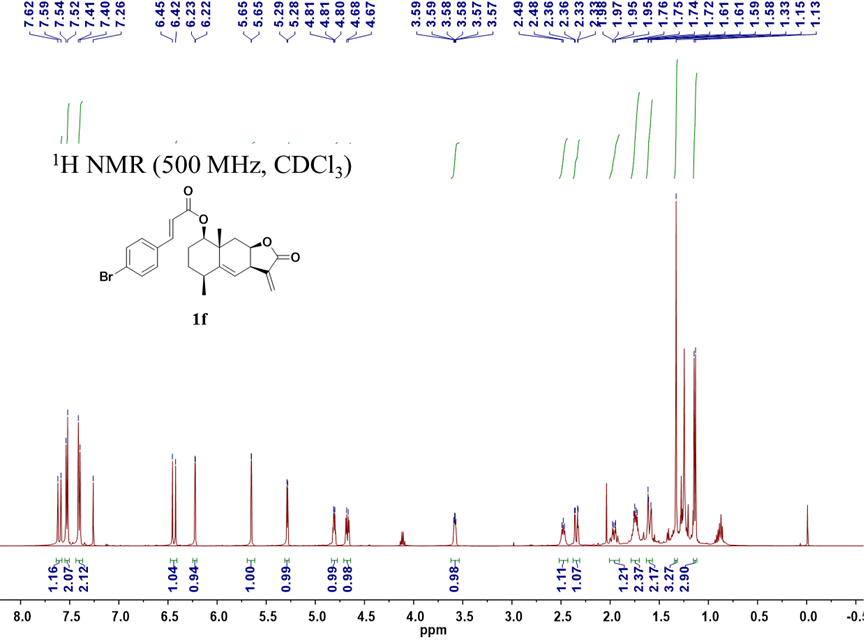


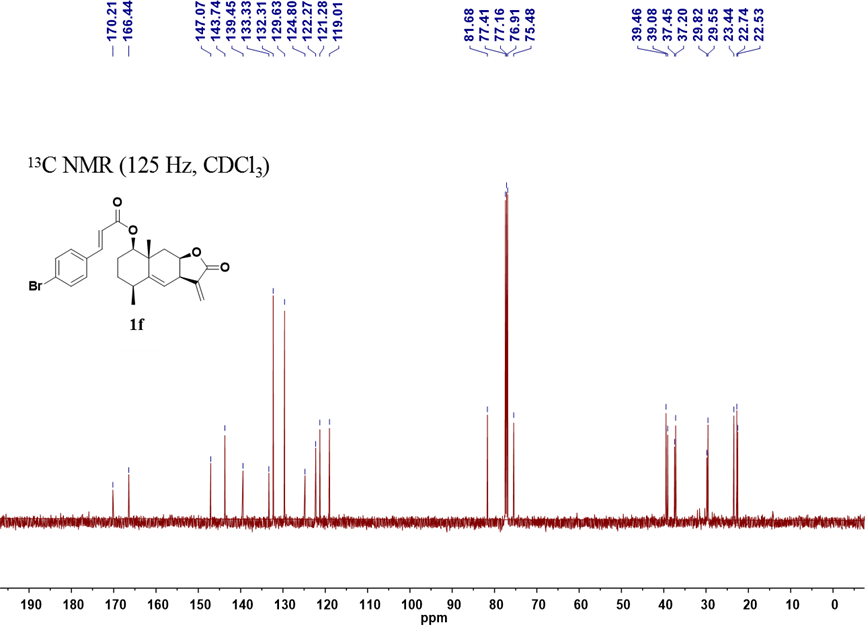


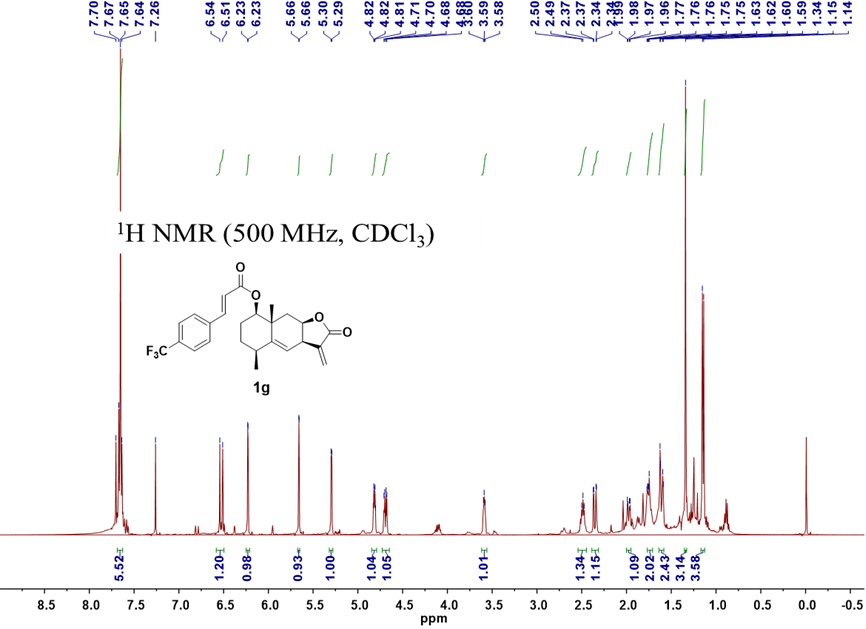

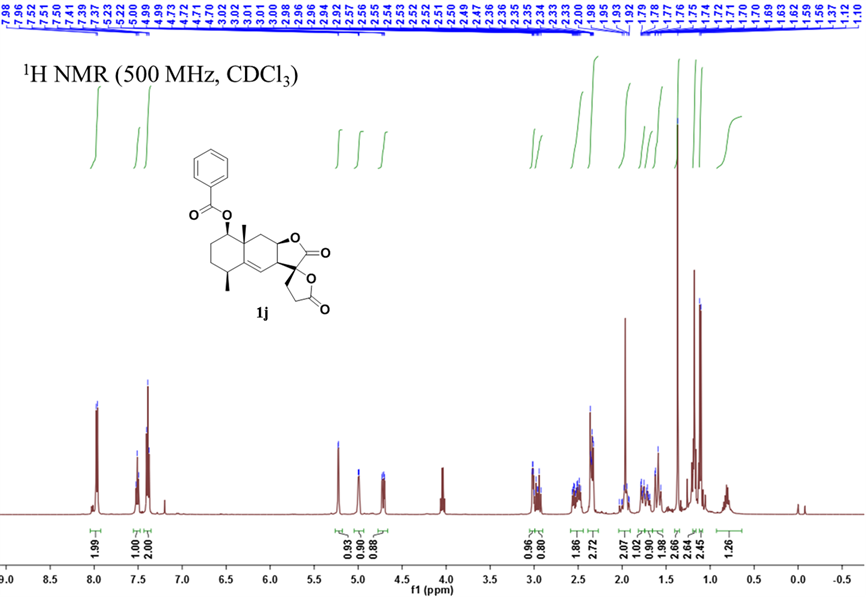


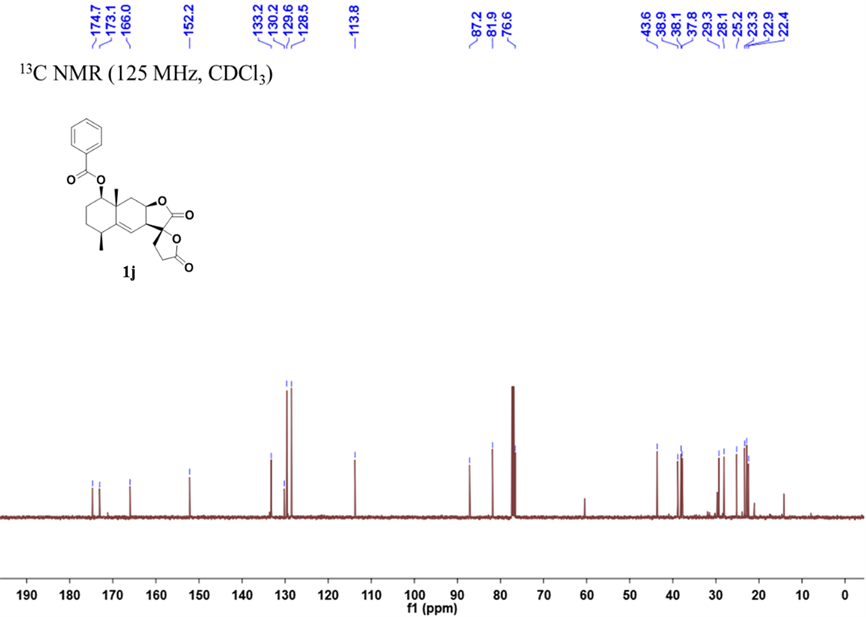


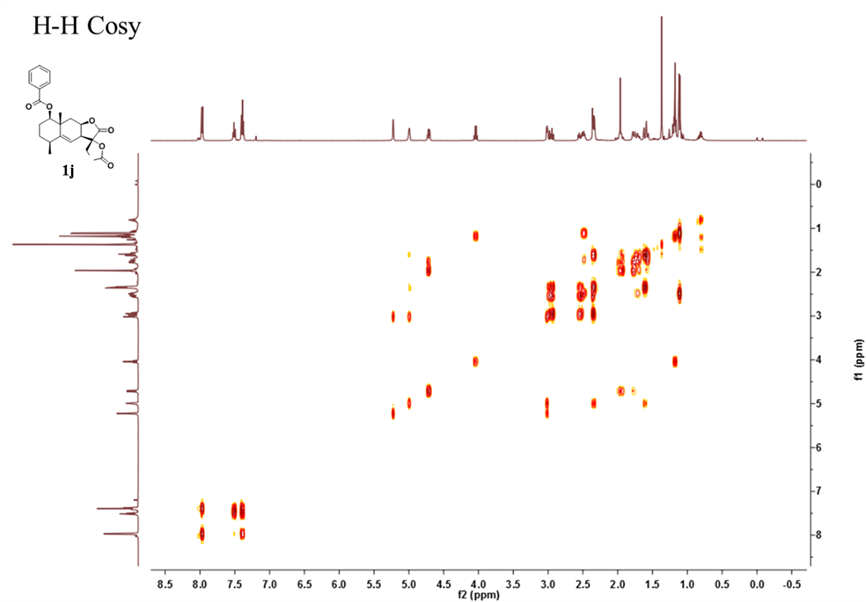


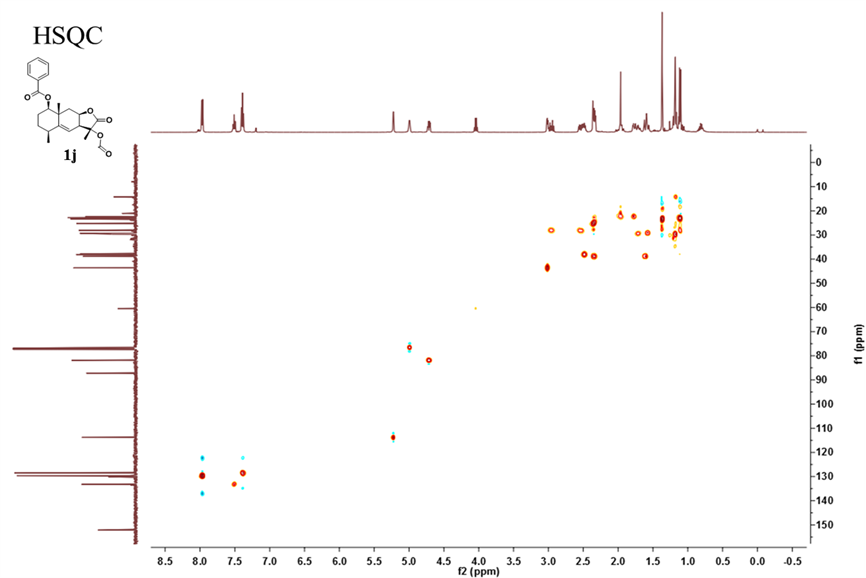


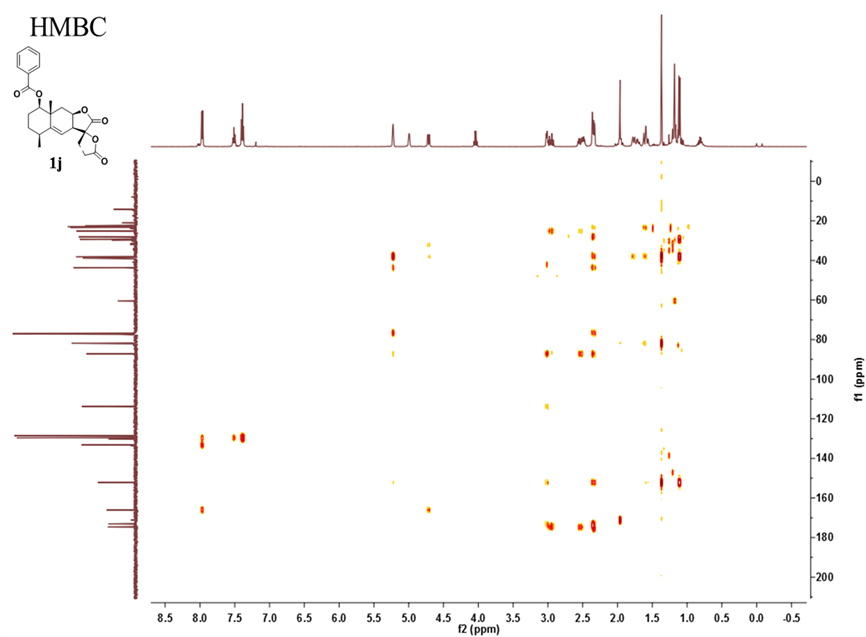


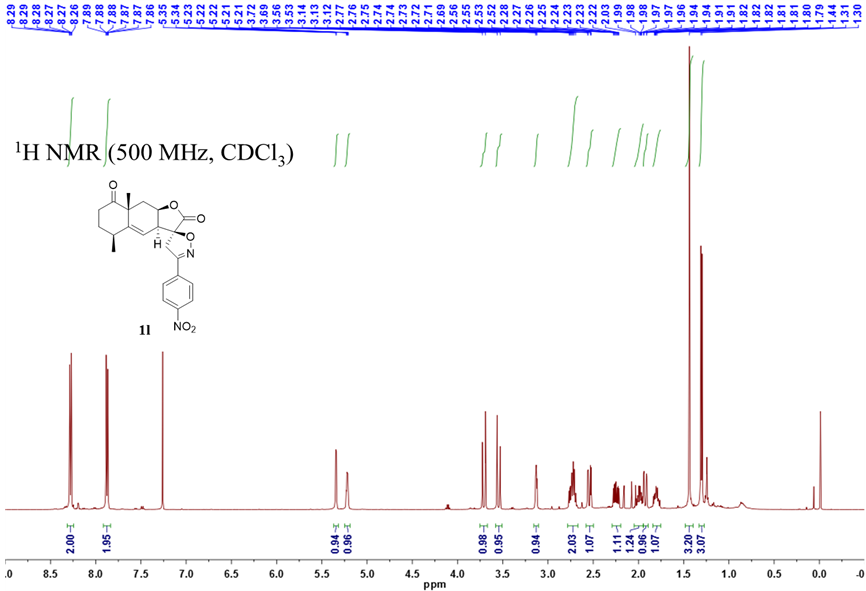


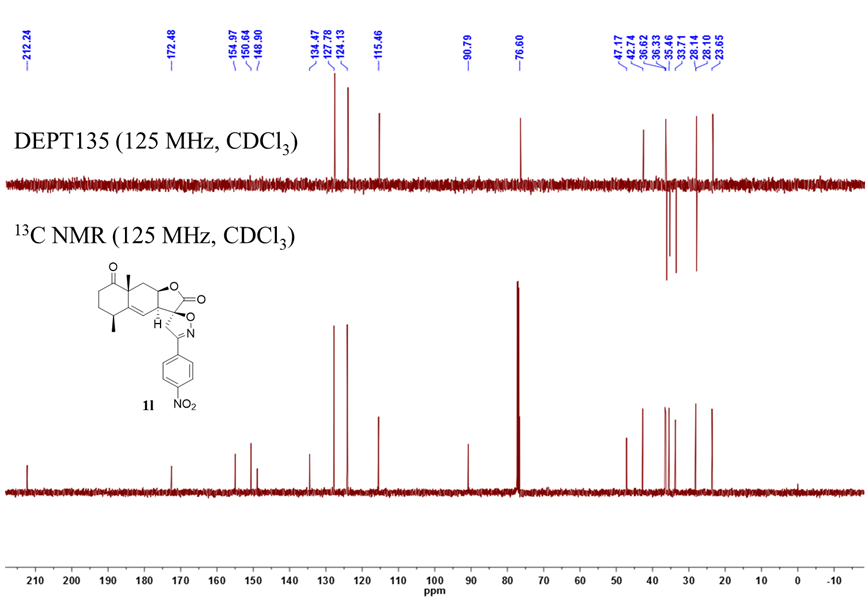


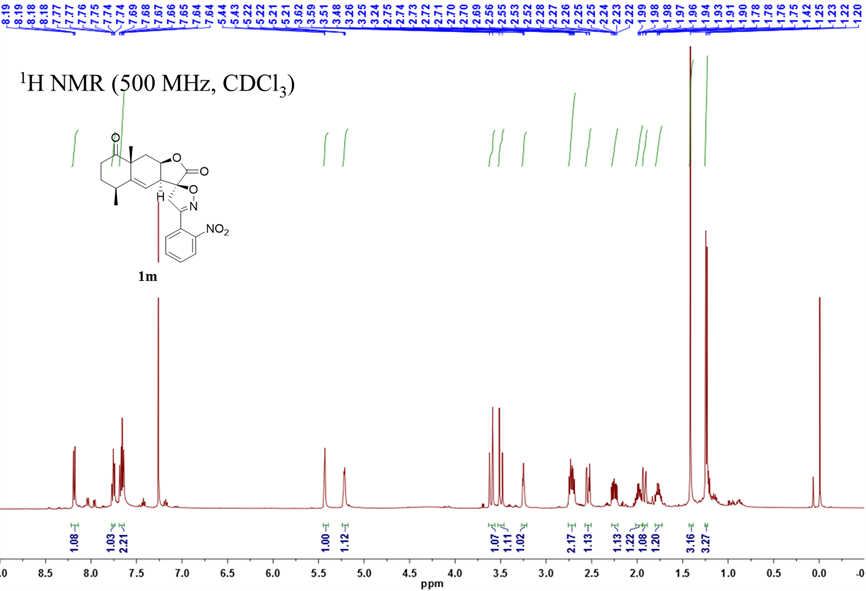


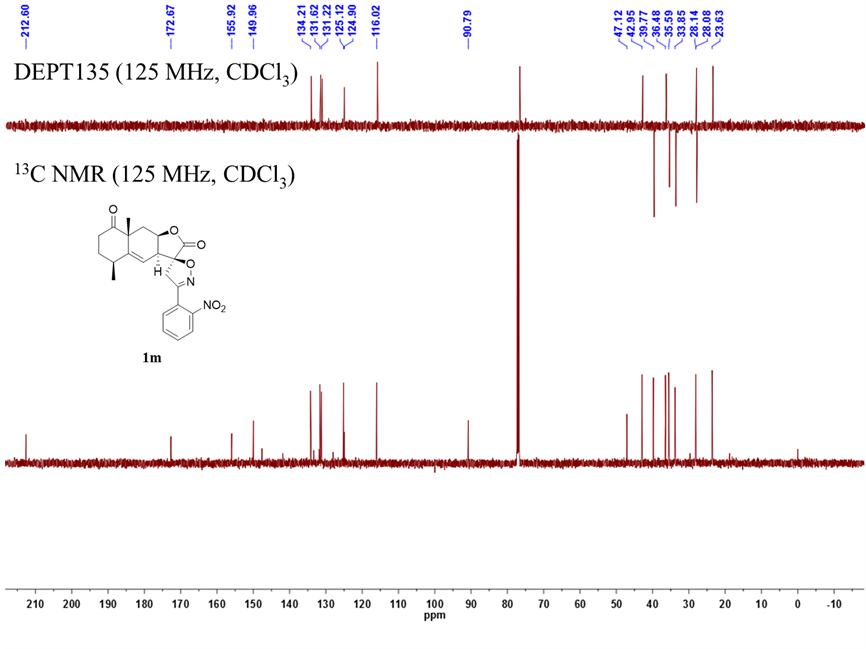


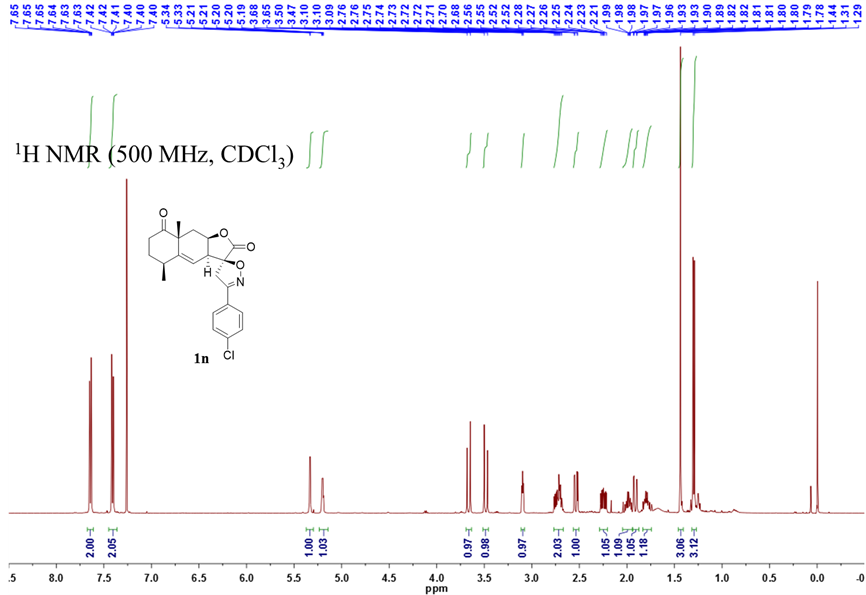


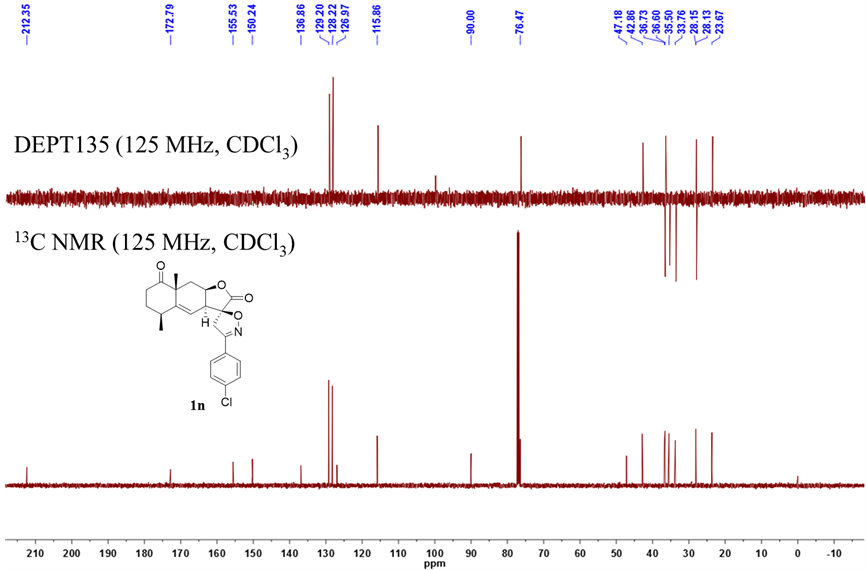


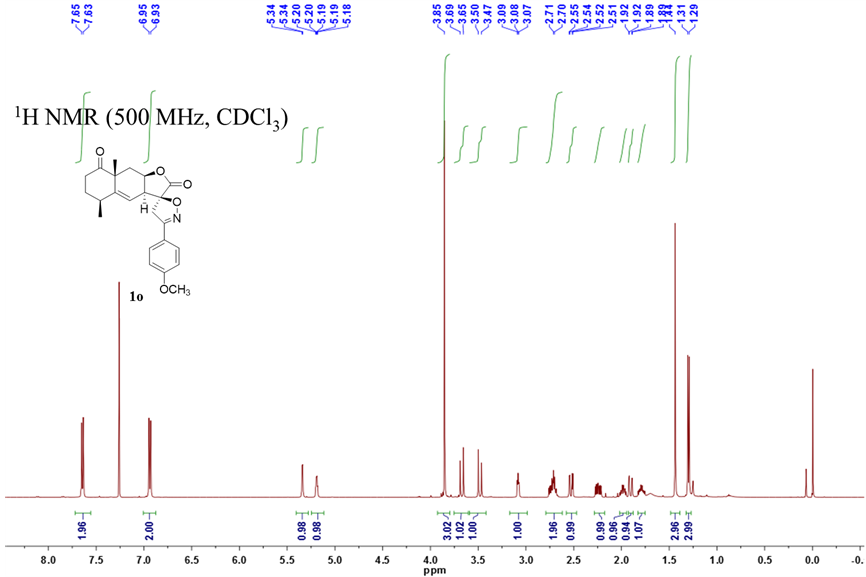

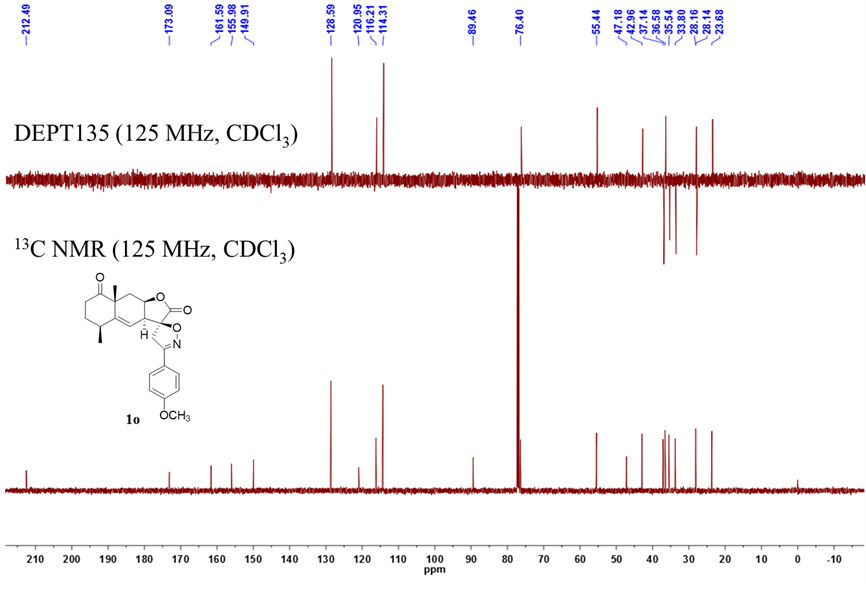


^1^H NMR (125 Hz, CDCl_3_)


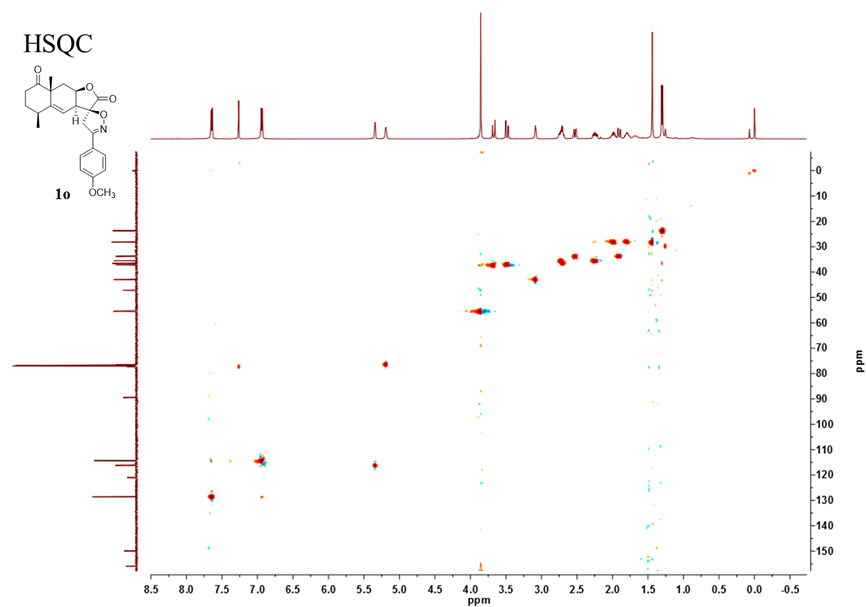


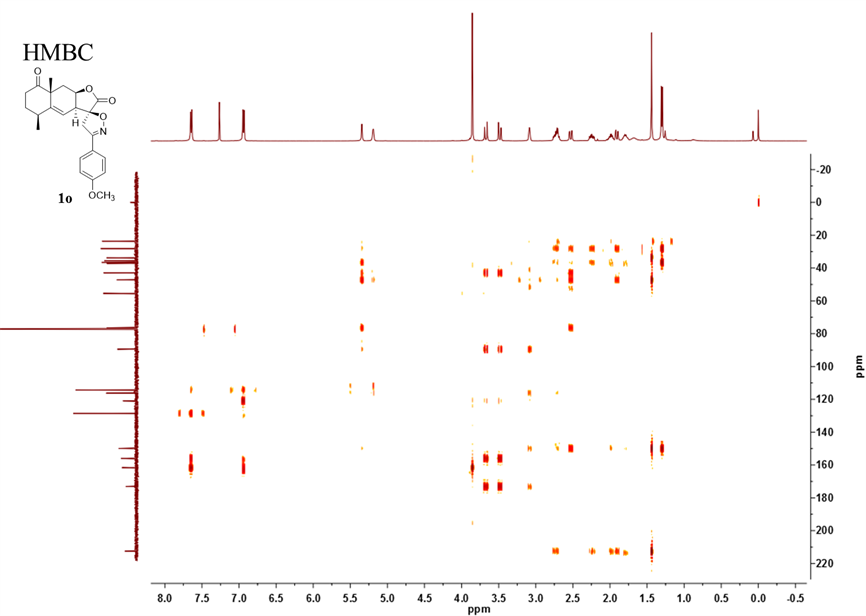


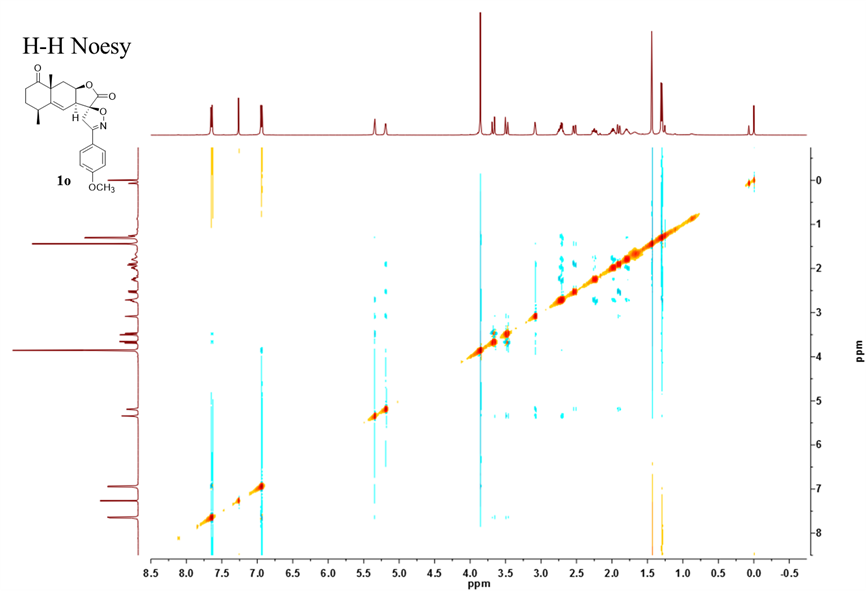


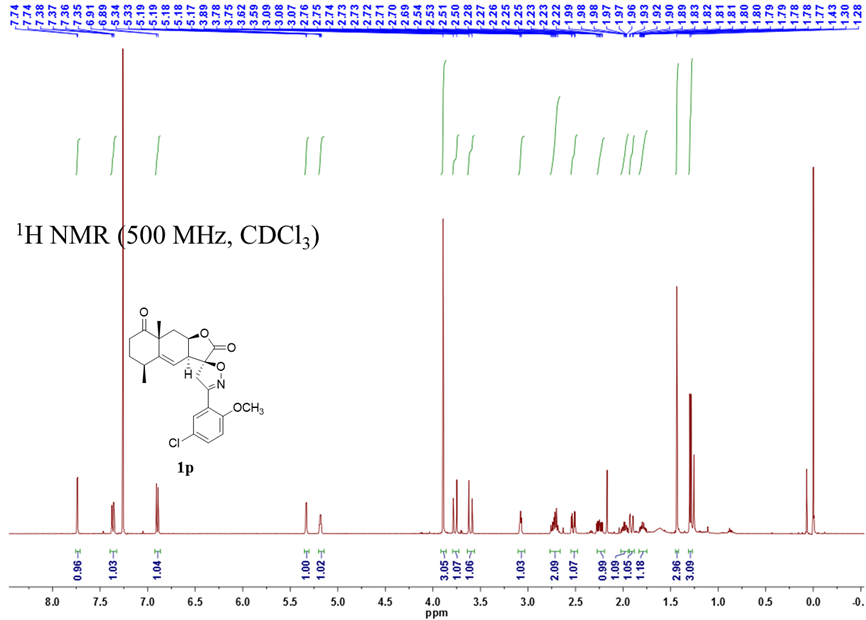


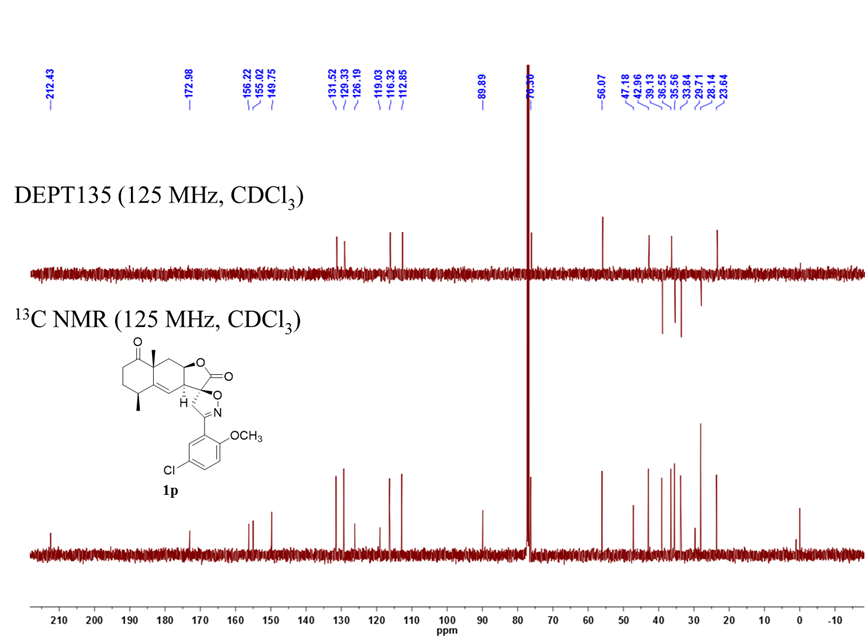


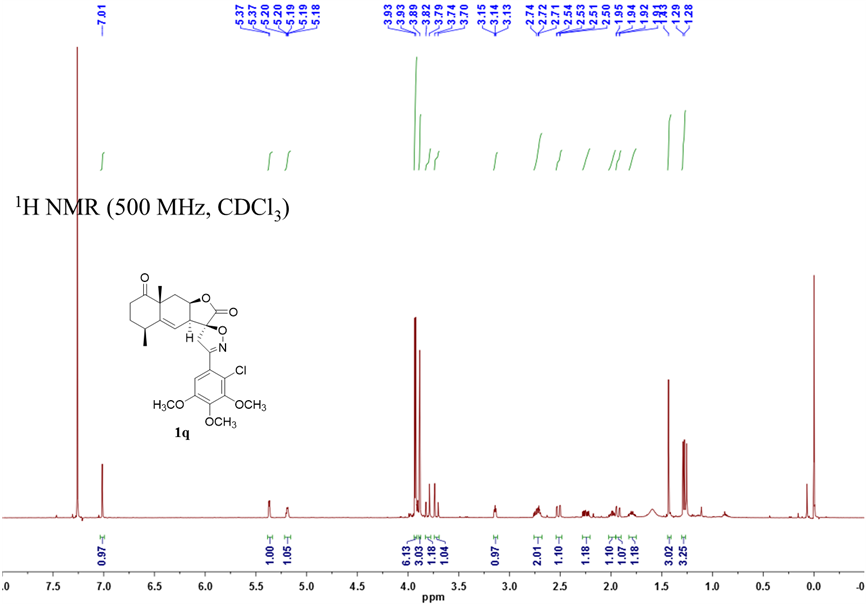


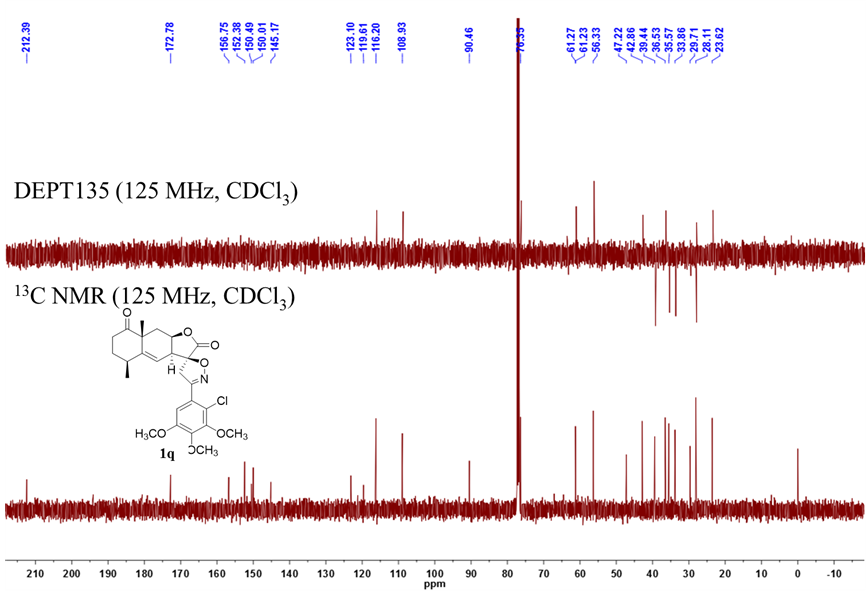


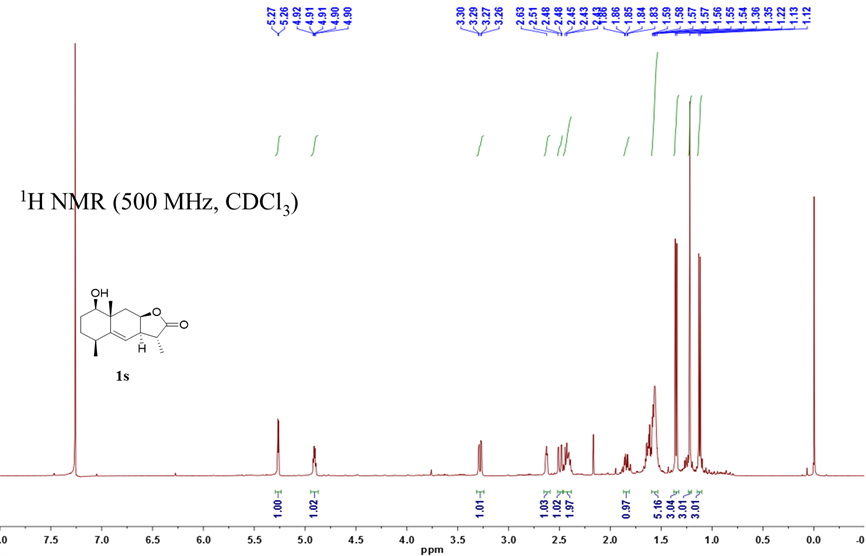


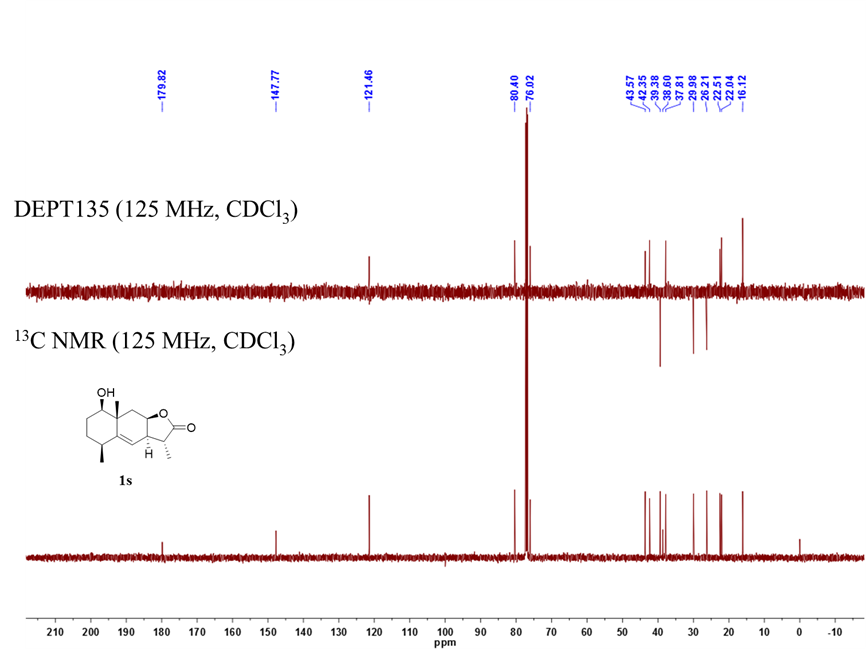


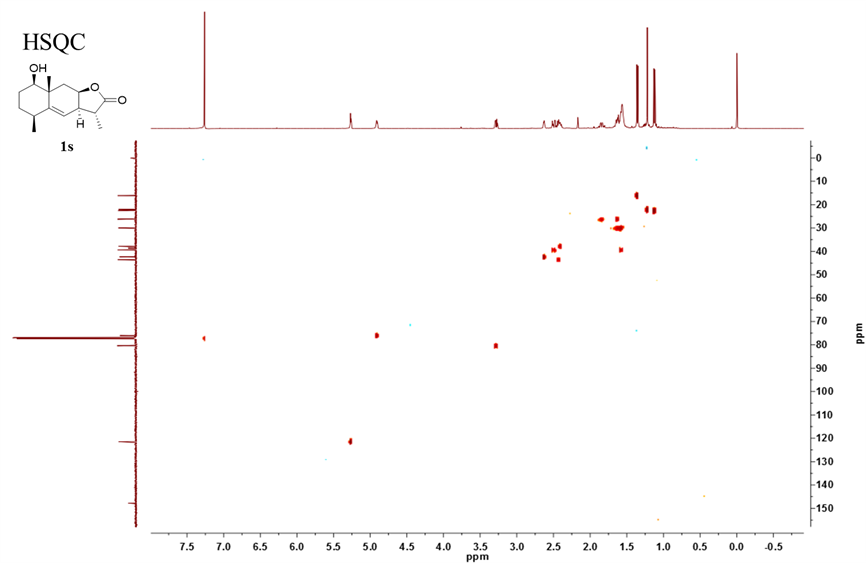


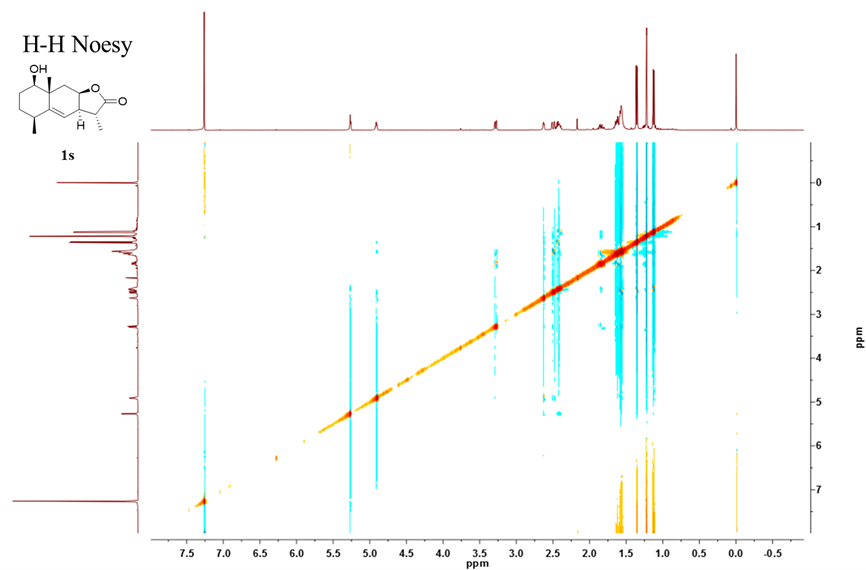


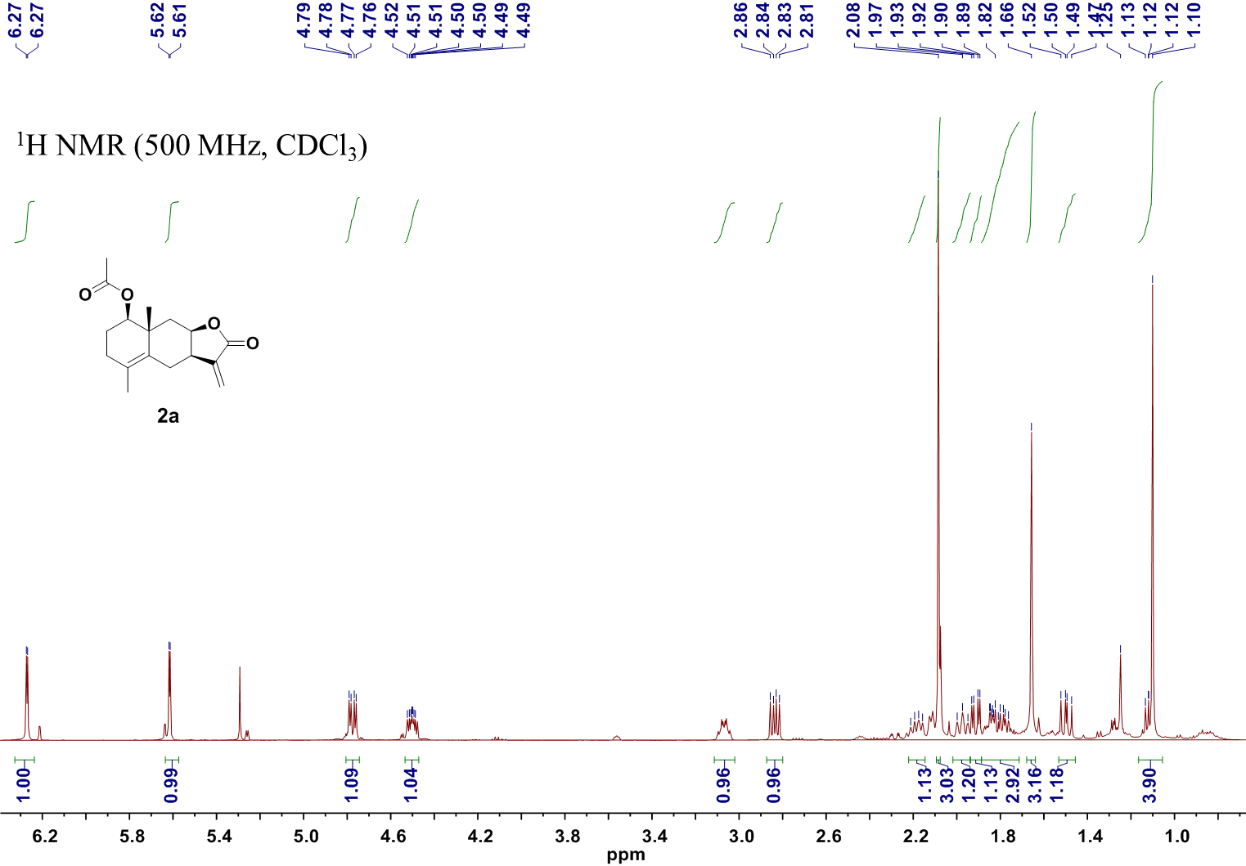


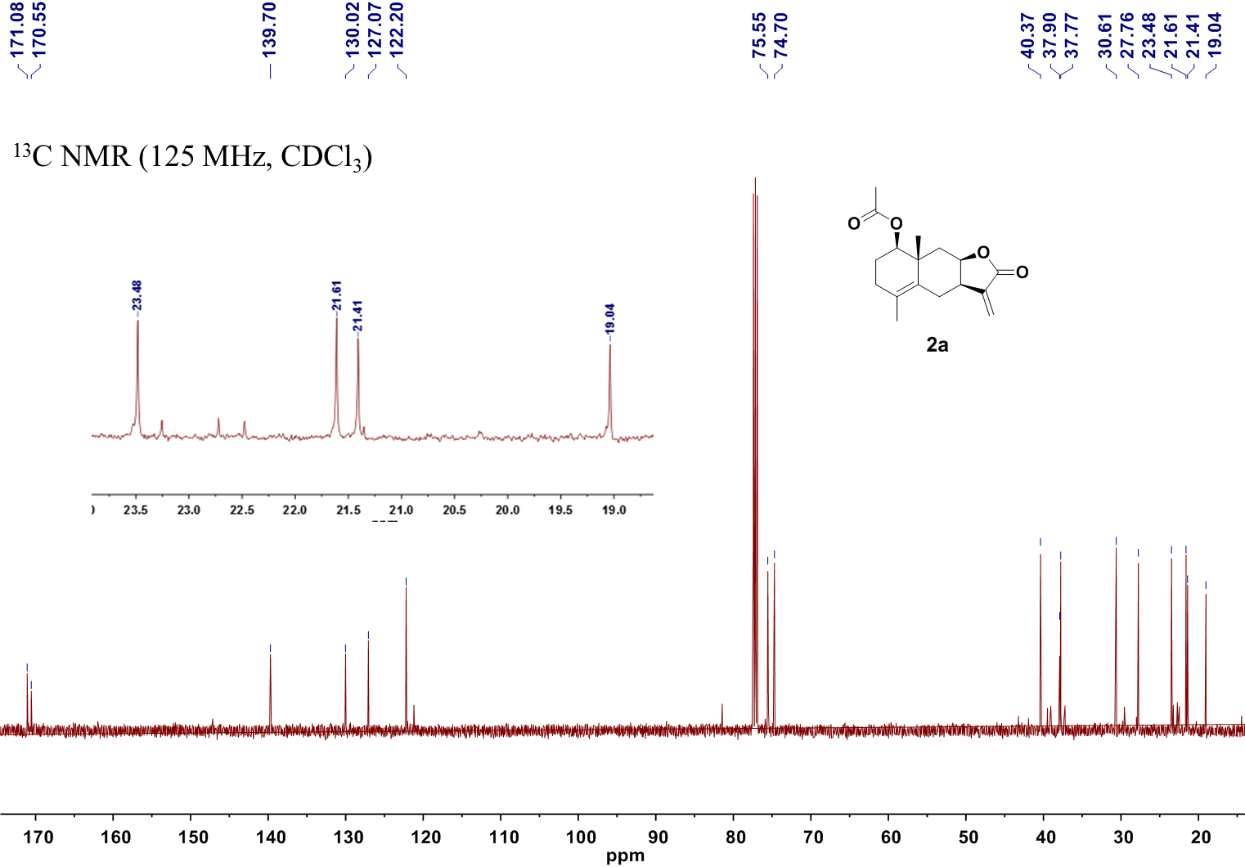


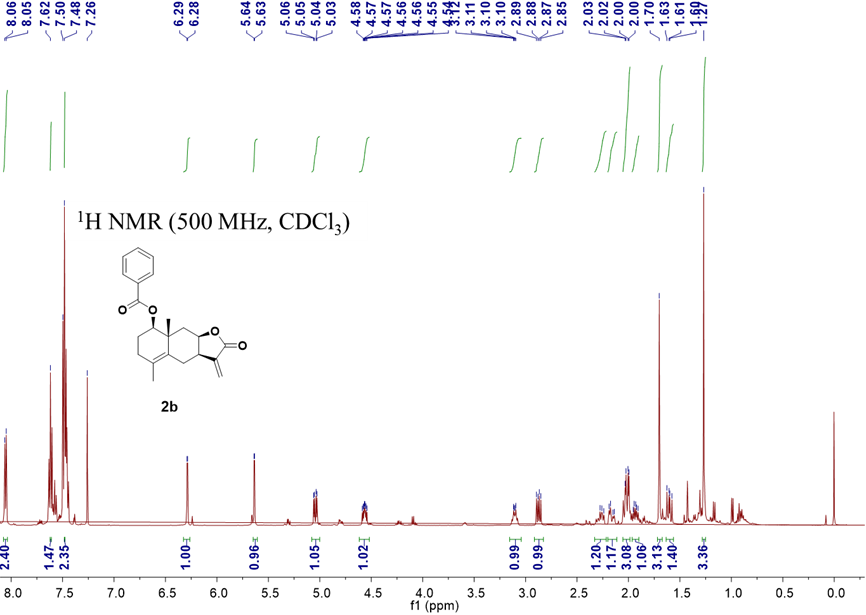

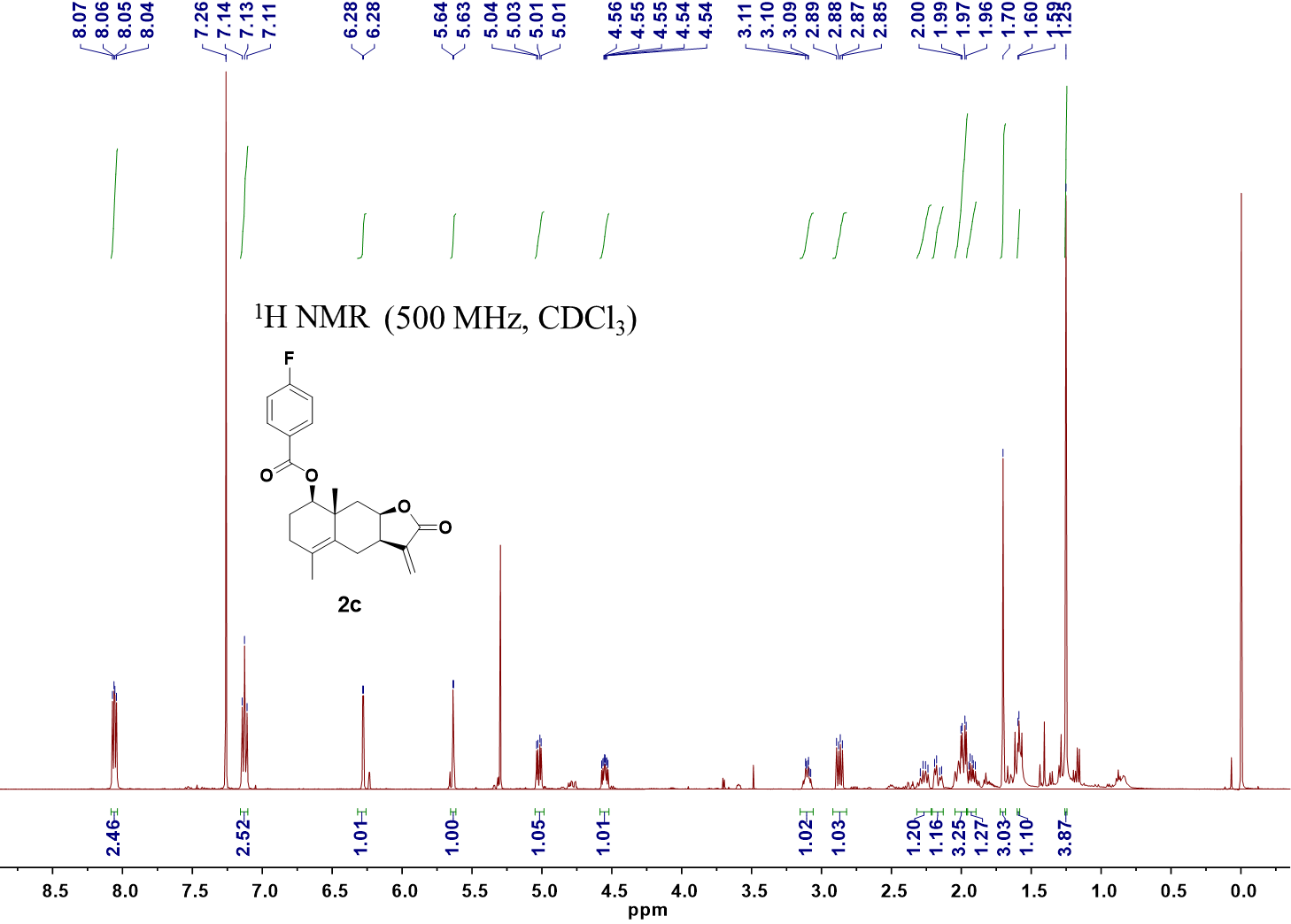

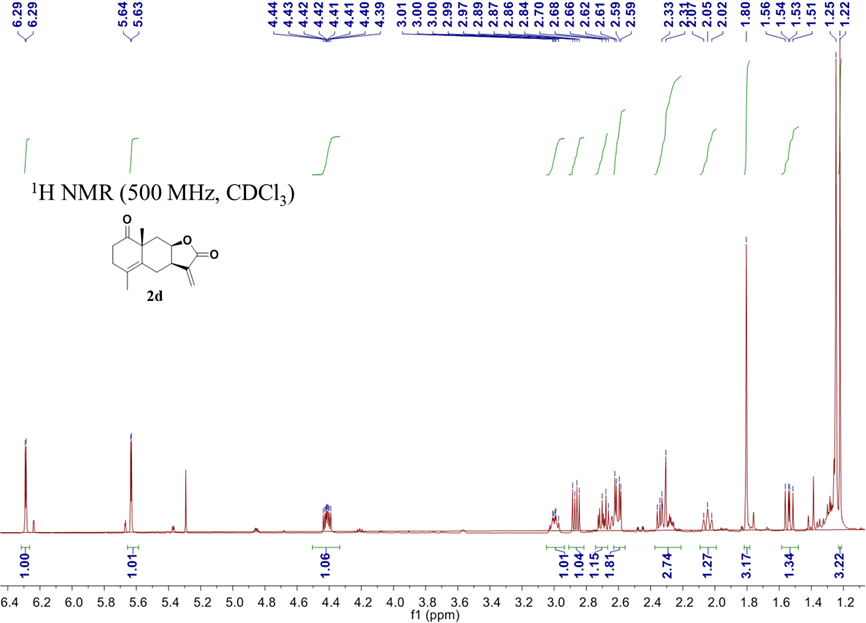


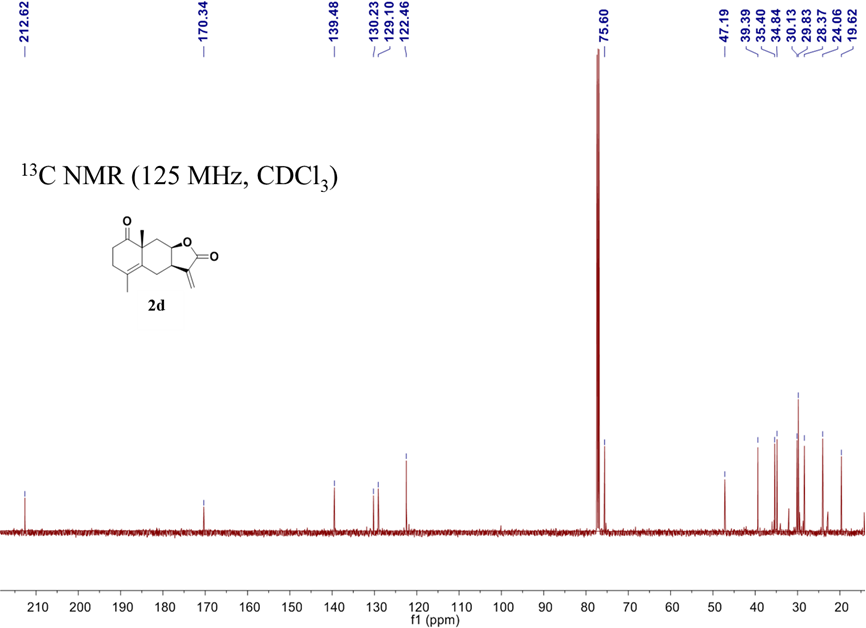


(**HR)-ESI-MS spectra**


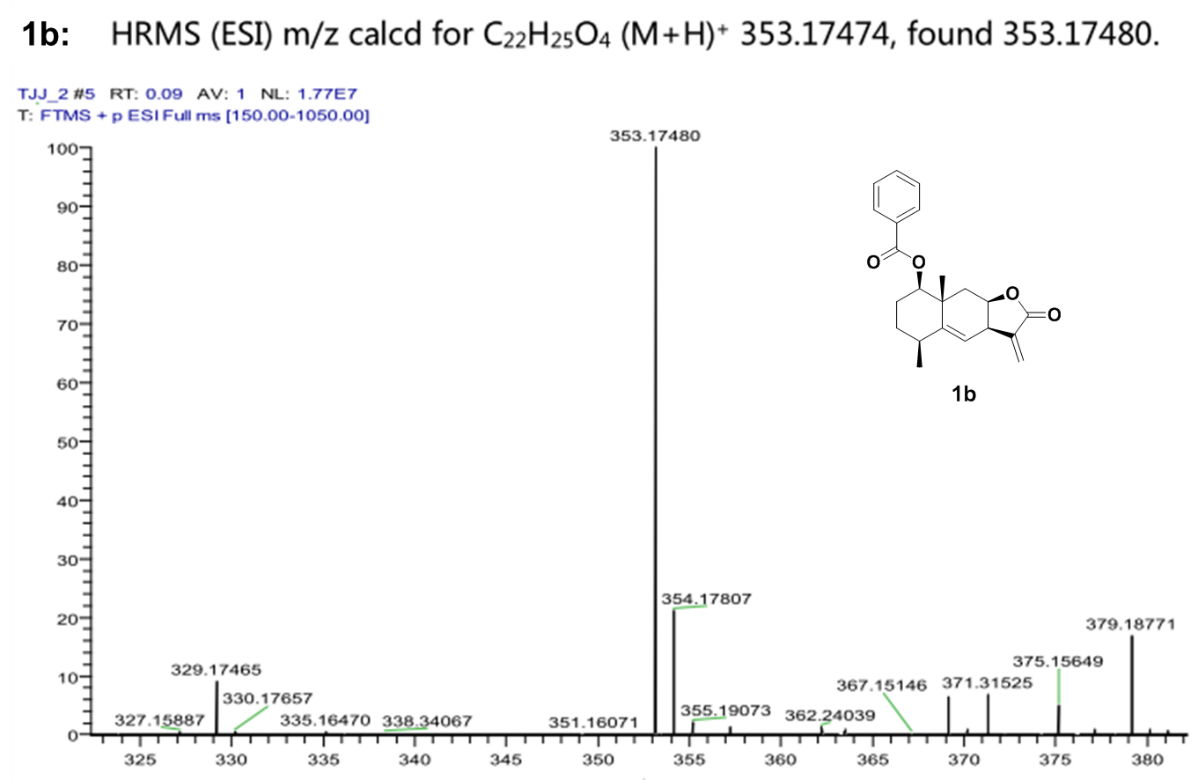


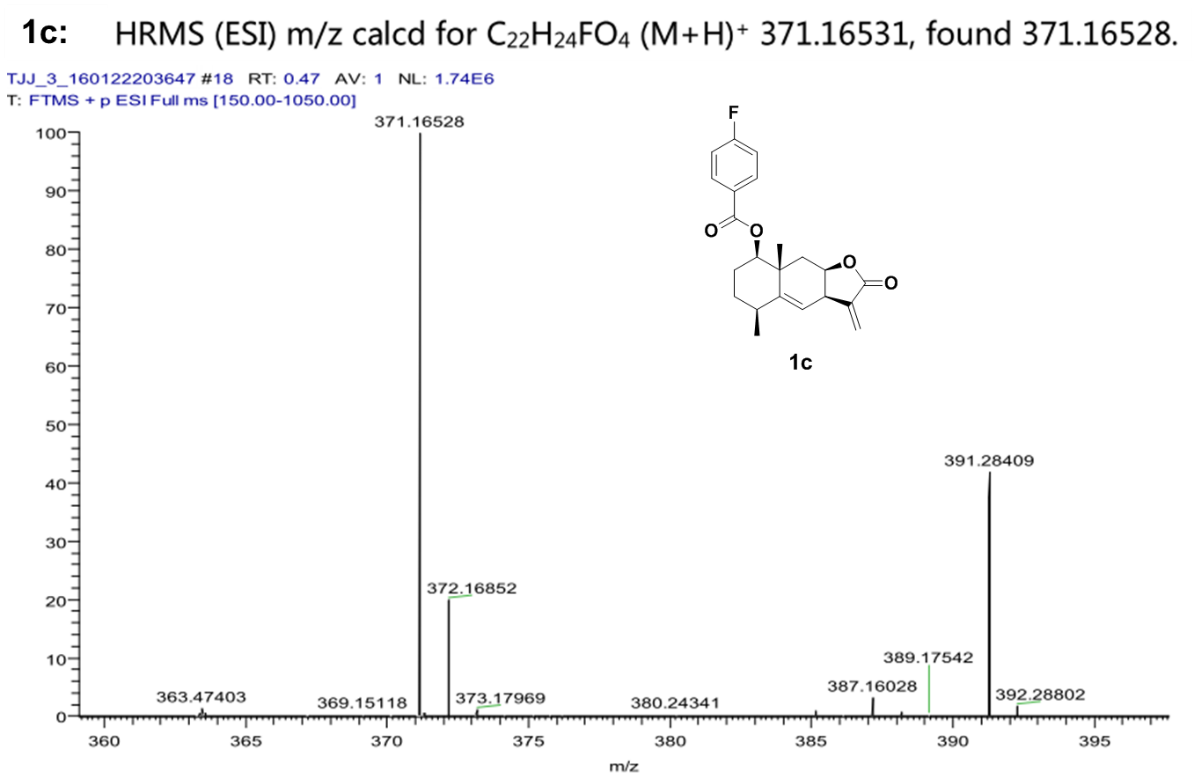


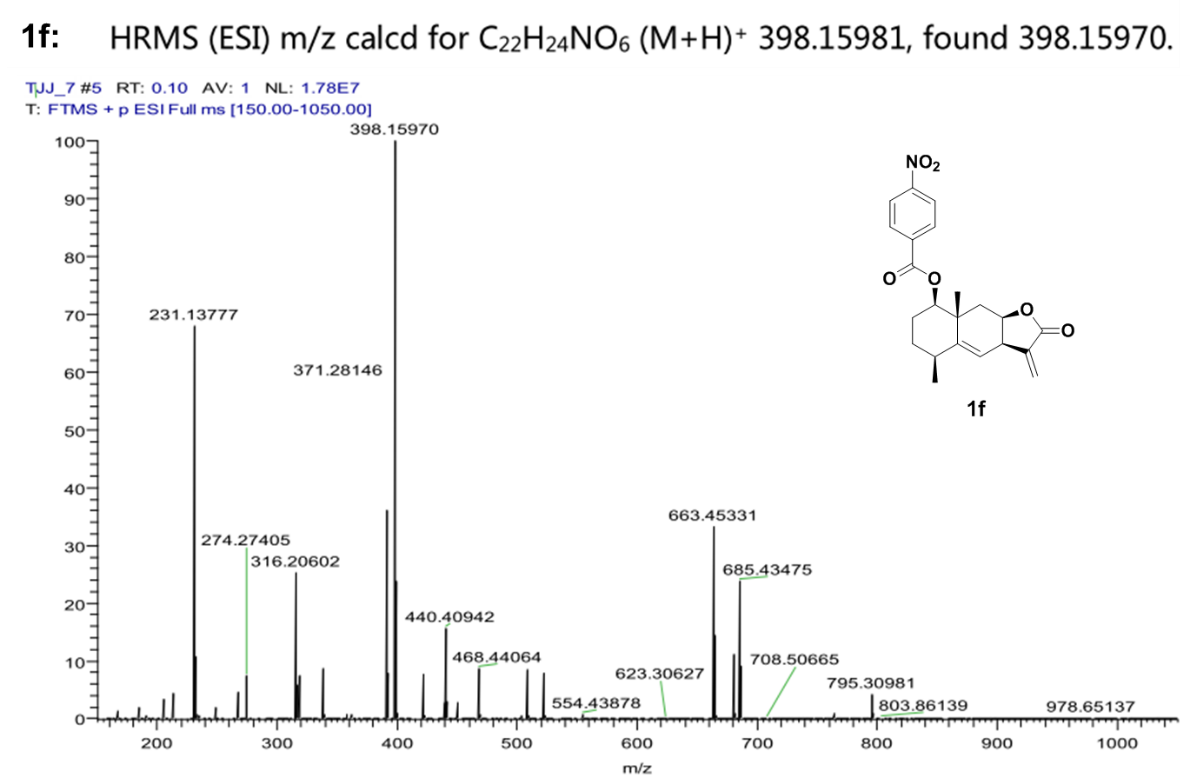


**1d：**

**1d**


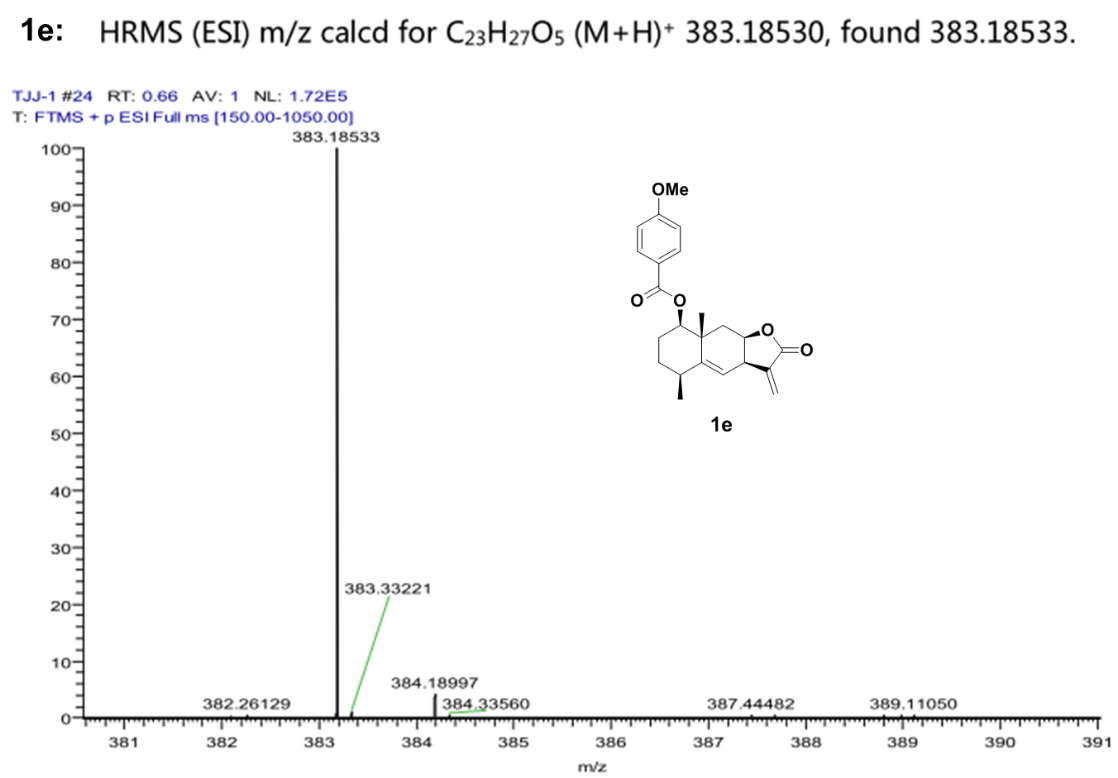


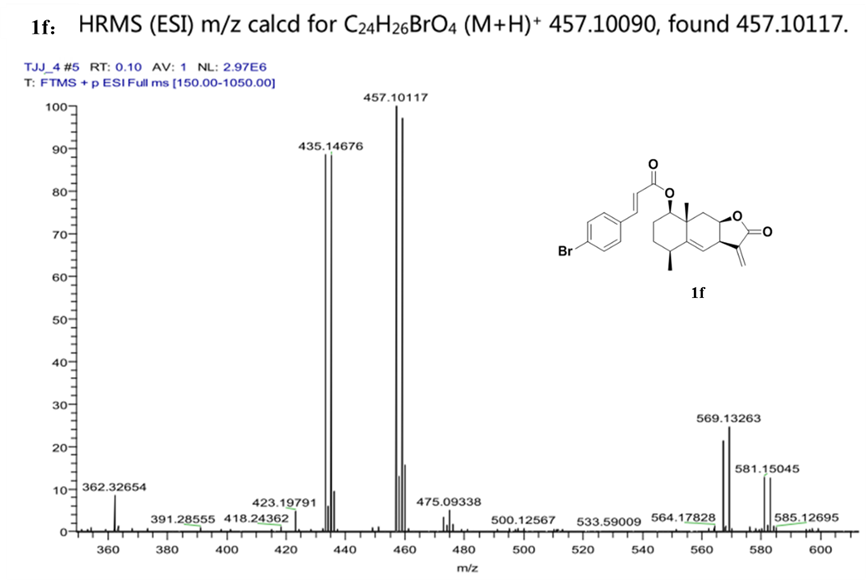


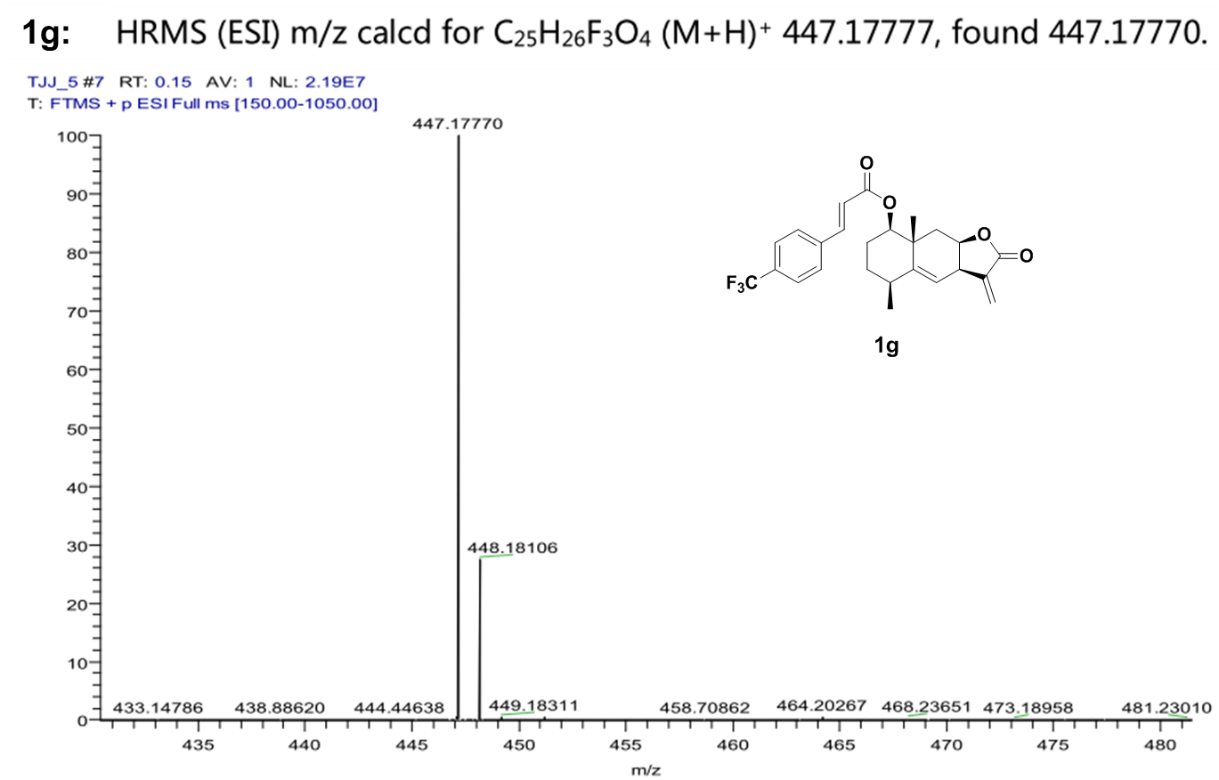


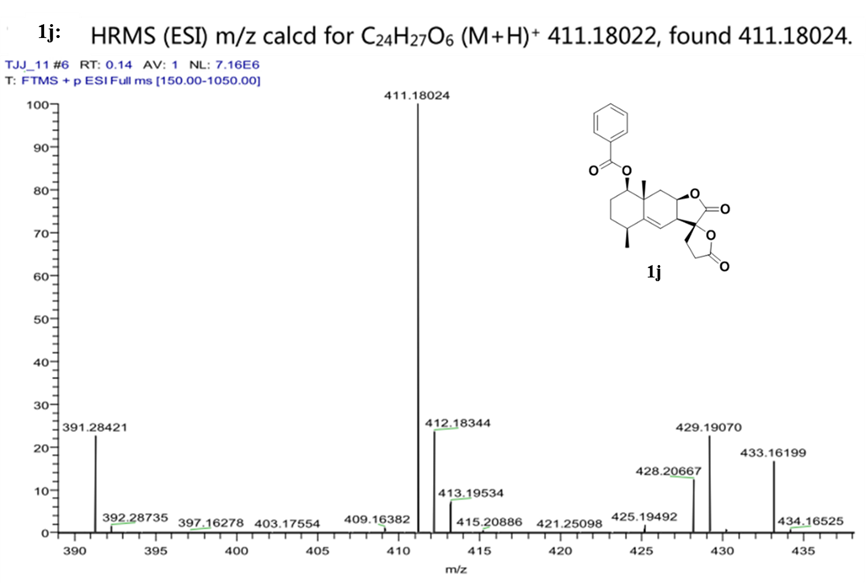


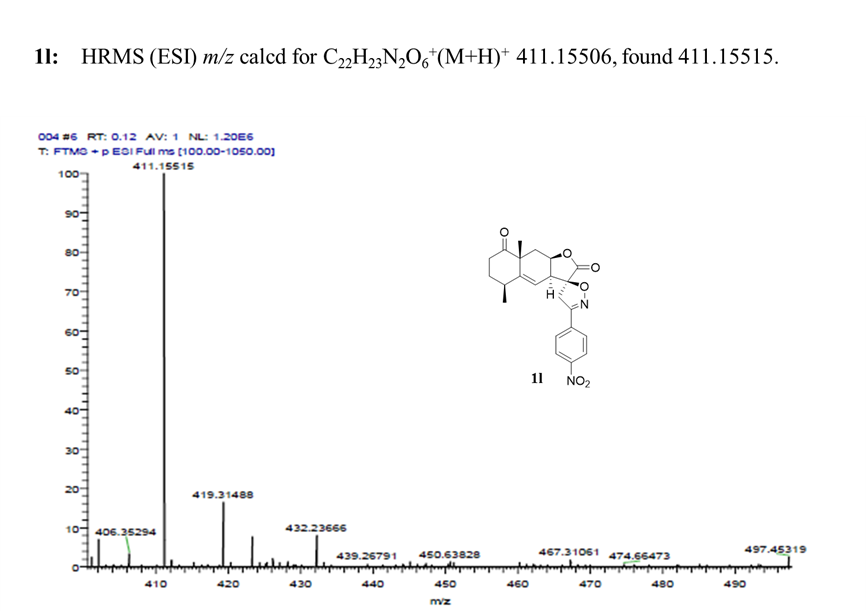


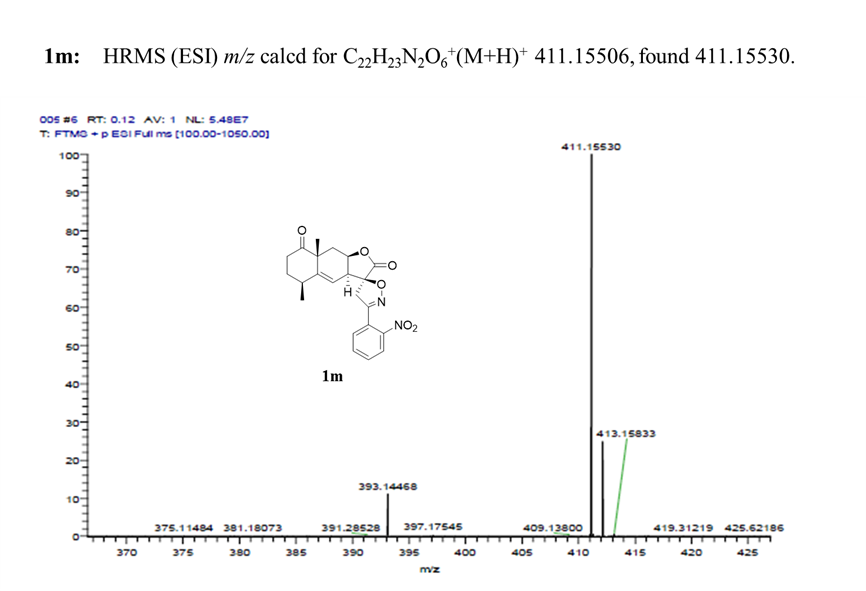


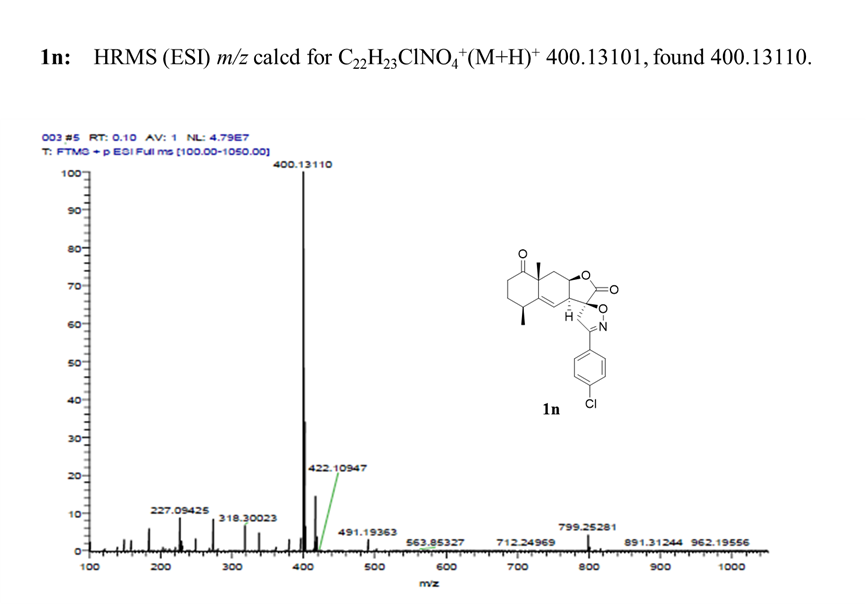


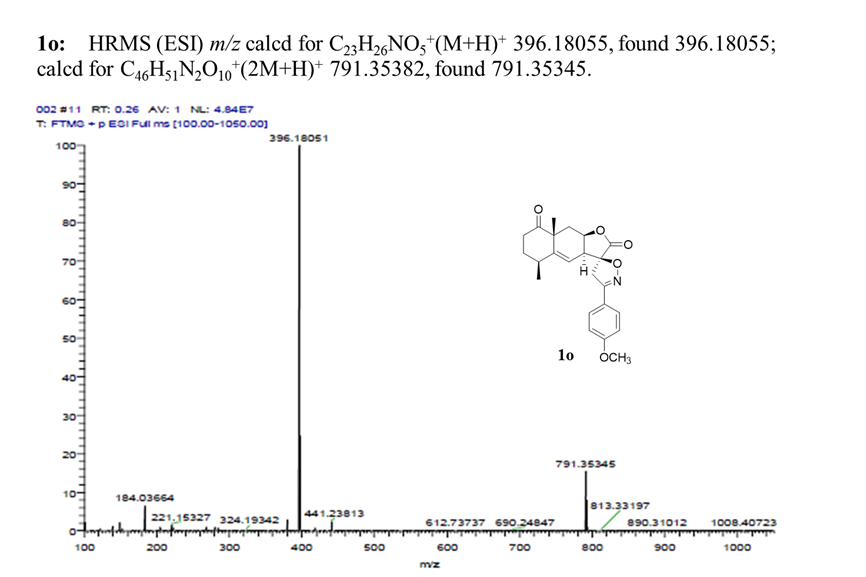


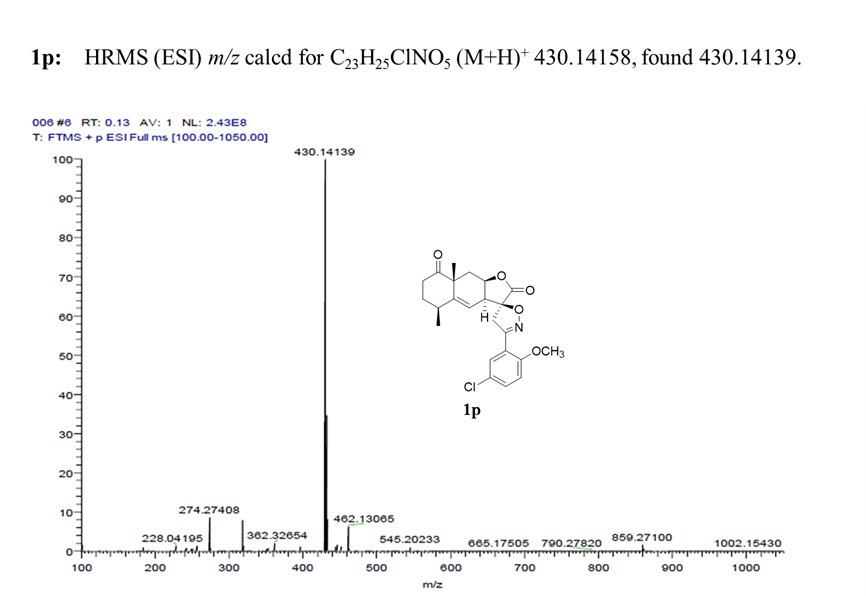


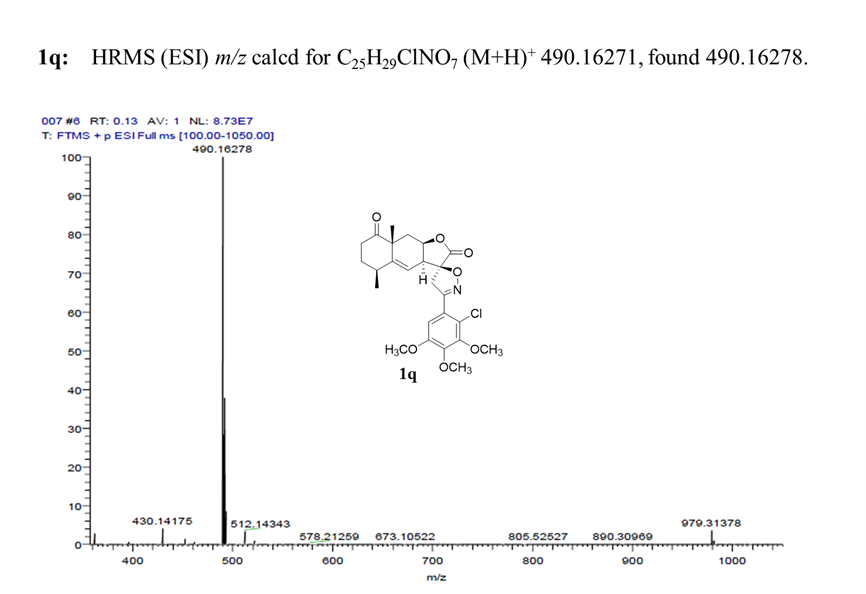


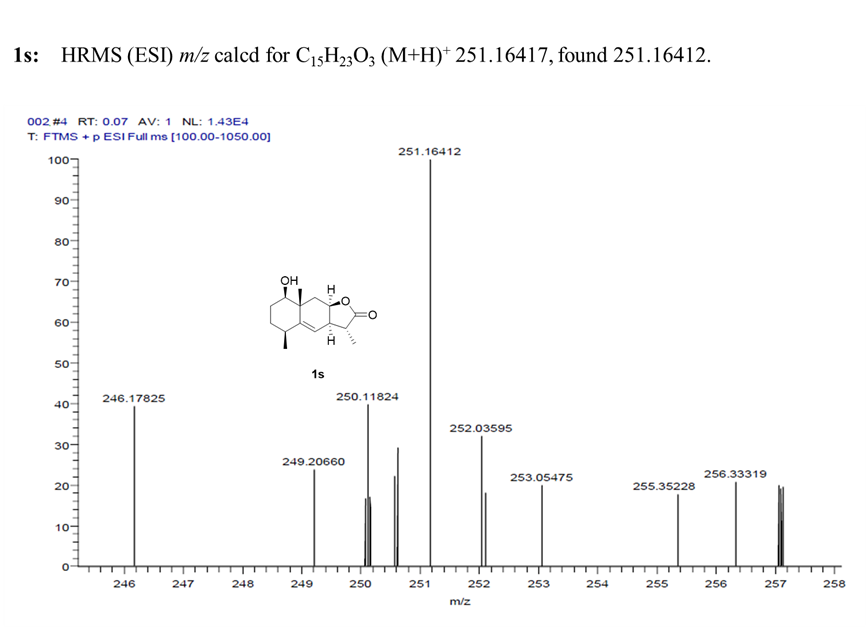


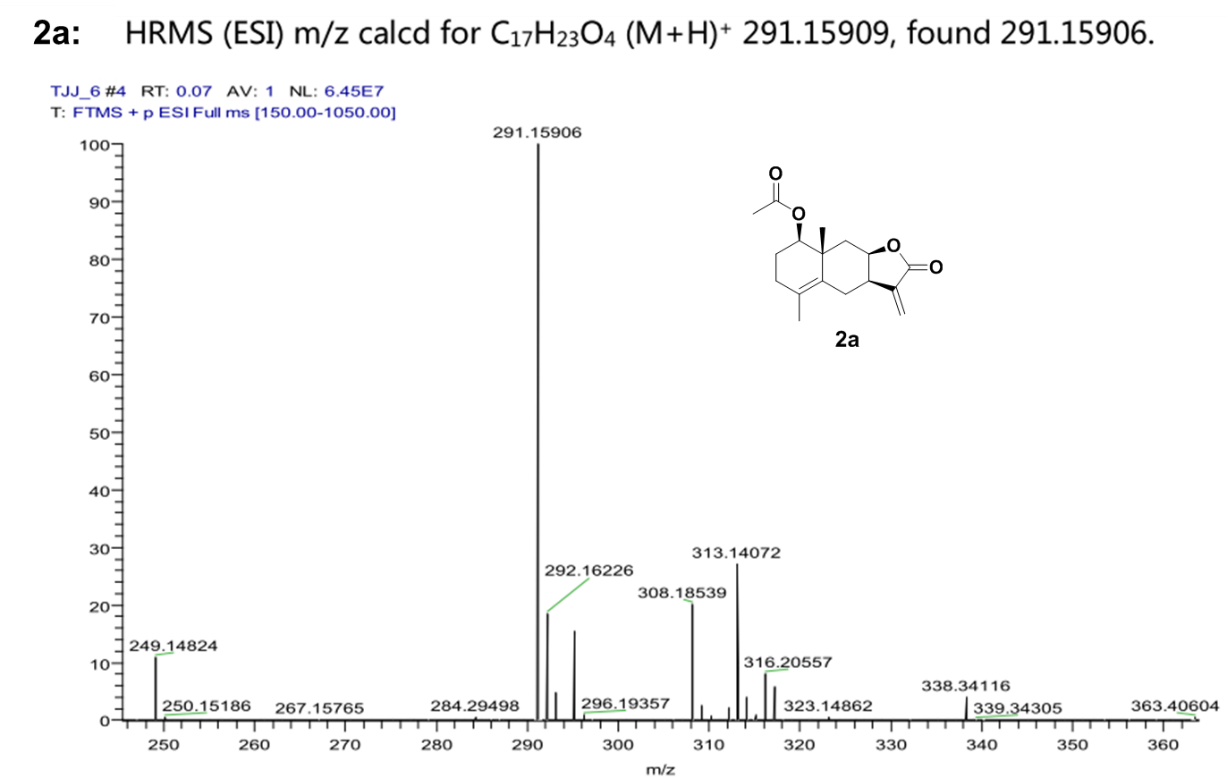


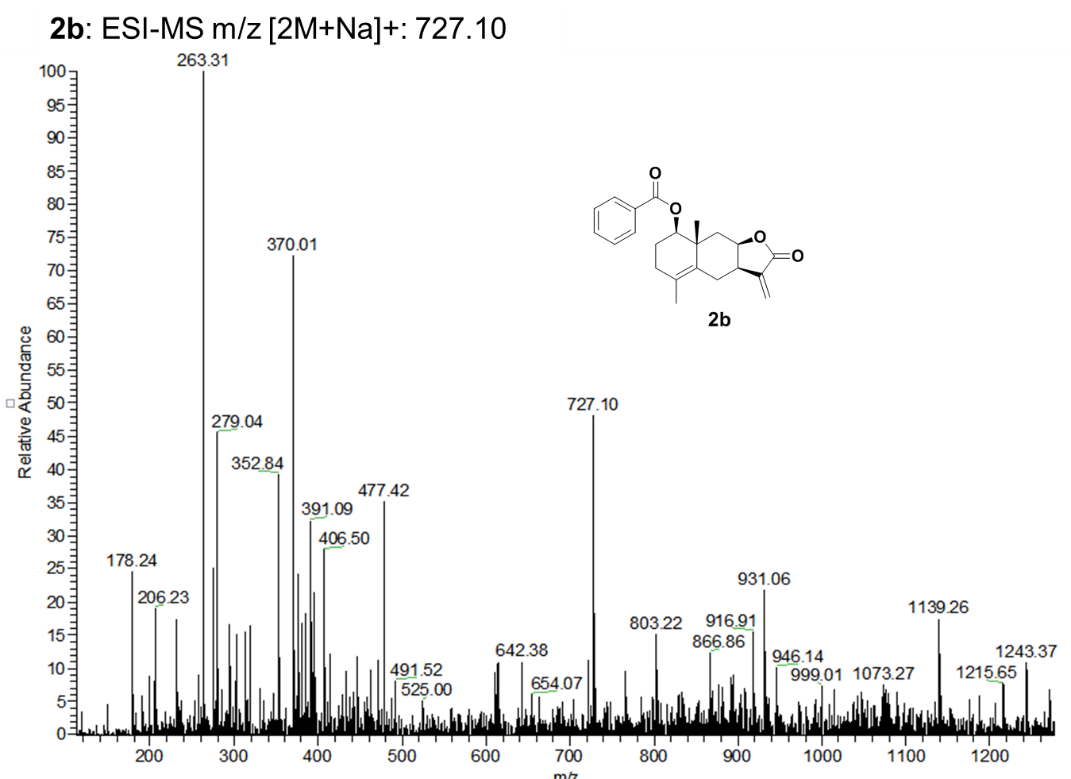


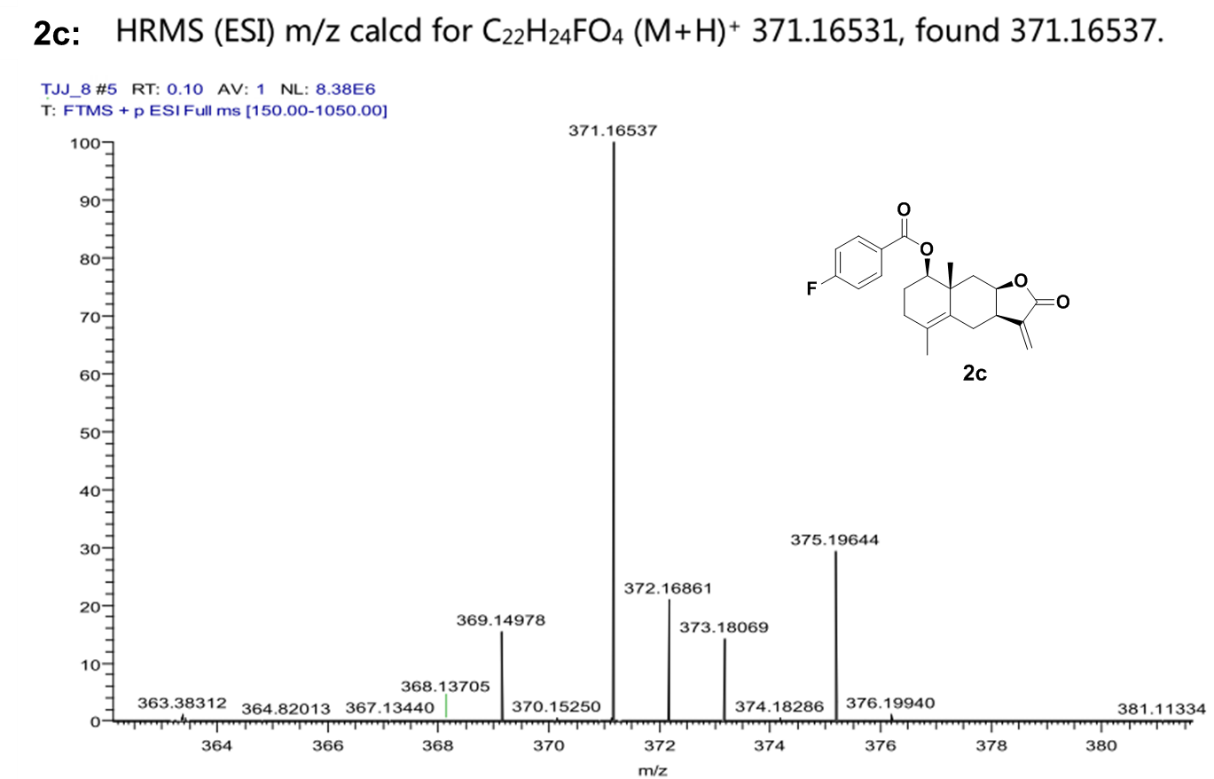


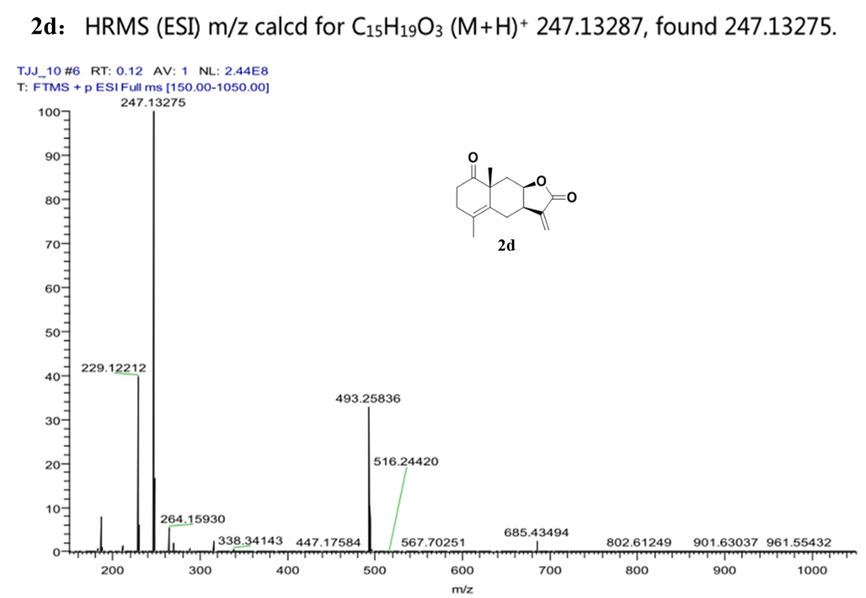


**HPLC purity determination**

**1:** Purity 96.1%, t_R_ = 11.9 min at 215 nm by RP-HPLC using 50% methanol in water.

**2:** Purity 96.9%, t_R_ = 15.7 min at 260 nm by RP-HPLC using 50% methanol in water.

1

**1b:** Purity 97.3%, t_R_ = 12.4 min at 254 nm by RP-HPLC using 60% methanol in water.

**1c:** Purity 97.6%, t_R_ = 14.2 min at 235 nm by RP-HPLC using 60% methanol in water.

**1d:** Purity 98.6%, t_R_ = 13.4 min at 225 nm by RP-HPLC using 60% methanol in water.

**1e:** Purity 99.0%, t_R_ = 14.1 min at 254 nm by RP-HPLC using 60% methanol in water.

**1f:** Purity 97.5%, t_R_ = 35.4 min at 285 nm by RP-HPLC using 50 min gradient from 0% to 100% methanol in water.

**1g:** Purity 95.8%, t_R_ = 36.5 min at 285 nm by RP-HPLC using 50 min gradient from 0% to 100% methanol in water.

**1h:** Purity 95.4%, t_R_ = 23.1 min at 210 nm by RP-HPLC using 50 min gradient from 0% to 100% methanol in water.

**1i:** Purity 97.0%, t_R_ = 10.2 min at 230 nm by RP-HPLC using 50% methanol in water.

**1j:** Purity 99.0%, t_R_ = 32.8 min at 230 nm by RP-HPLC using 50 min gradient from 0% to 100% methanol in water.

**1k:** Purity 95.1%, t_R_ = 39.0 min at 270 nm by RP-HPLC using 50 min gradient from 0% to 100% methanol in water.

**1l:** Purity 97.0%, t_R_ = 38.6 min at 299 nm by RP-HPLC using 50 min gradient from 0% to 100% methanol in water.

**1m:** Purity 95.9%, t_R_ = 43.0 min at 212 nm by RP-HPLC using 50 min gradient from 0% to 100% methanol in water.

**1n:** Purity 95.3%, t_R_ = 39.8 min at 269 nm by RP-HPLC using 50 min gradient from 0% to 100% methanol in water.

**1o:** Purity 95.5%, t_R_ = 39.7 min at 278 nm by RP-HPLC using 50 min gradient from 0% to 100% methanol in water.

**1p:** Purity 96.0%, t_R_ = 40.0 min at 230 nm by RP-HPLC using 50 min gradient from 0% to 100% methanol in water.

**1q:** Purity 95.2%, t_R_ = 39.7 min at 226 nm by RP-HPLC using 50 min gradient from 0% to 100% methanol in water.

**1r:** Purity 98.5%, t_R_ = 34.4 min at 210 nm by RP-HPLC using 50 min gradient from 0% to 100% methanol in water.

**1s:** Purity 98.3%, t_R_ = 34.4 min at 210 nm by RP-HPLC using 50 min gradient from 0% to 100% methanol in water.

**2a:** Purity 99.9%, t_R_ = 25.5 min at 320 nm by RP-HPLC using 50% methanol in water.

**2b:** Purity 95.3%, t_R_ = 34.0 min at 254 nm by RP-HPLC using 50 min gradient from 0% to 100% methanol in water.

**2c:** Purity 99.7%, t_R_ = 10.3 min at 230 nm by RP-HPLC using 60% methanol in water.

**2d:** Purity 98.6%, t_R_ = 5.8 min at 254 nm by RP-HPLC using 60% methanol in water.
